# Identification of a differentiation stall in epithelial mesenchymal transition in histone H3–mutant diffuse midline glioma

**DOI:** 10.1093/gigascience/giaa136

**Published:** 2020-12-15

**Authors:** Lauren M Sanders, Allison Cheney, Lucas Seninge, Anouk van den Bout, Marissa Chen, Holly C Beale, Ellen Towle Kephart, Jacob Pfeil, Katrina Learned, A Geoffrey Lyle, Isabel Bjork, David Haussler, Sofie R Salama, Olena M Vaske

**Affiliations:** Department of Biomolecular Engineering, University of California Santa Cruz, 1156 High Street, Santa Cruz, CA 95064, USA; University of California Santa Cruz Genomics Institute, University of California Santa Cruz, 1156 High Street, Santa Cruz, CA 95064, USA; Department of Molecular, Cell and Developmental Biology, University of California Santa Cruz, 1156 High Street, Santa Cruz, CA 95064, USA; Department of Biomolecular Engineering, University of California Santa Cruz, 1156 High Street, Santa Cruz, CA 95064, USA; University of California Santa Cruz Genomics Institute, University of California Santa Cruz, 1156 High Street, Santa Cruz, CA 95064, USA; University of California Santa Cruz Genomics Institute, University of California Santa Cruz, 1156 High Street, Santa Cruz, CA 95064, USA; Department of Molecular, Cell and Developmental Biology, University of California Santa Cruz, 1156 High Street, Santa Cruz, CA 95064, USA; University of California Santa Cruz Genomics Institute, University of California Santa Cruz, 1156 High Street, Santa Cruz, CA 95064, USA; Department of Molecular, Cell and Developmental Biology, University of California Santa Cruz, 1156 High Street, Santa Cruz, CA 95064, USA; University of California Santa Cruz Genomics Institute, University of California Santa Cruz, 1156 High Street, Santa Cruz, CA 95064, USA; Department of Molecular, Cell and Developmental Biology, University of California Santa Cruz, 1156 High Street, Santa Cruz, CA 95064, USA; University of California Santa Cruz Genomics Institute, University of California Santa Cruz, 1156 High Street, Santa Cruz, CA 95064, USA; Department of Biomolecular Engineering, University of California Santa Cruz, 1156 High Street, Santa Cruz, CA 95064, USA; University of California Santa Cruz Genomics Institute, University of California Santa Cruz, 1156 High Street, Santa Cruz, CA 95064, USA; University of California Santa Cruz Genomics Institute, University of California Santa Cruz, 1156 High Street, Santa Cruz, CA 95064, USA; University of California Santa Cruz Genomics Institute, University of California Santa Cruz, 1156 High Street, Santa Cruz, CA 95064, USA; Department of Molecular, Cell and Developmental Biology, University of California Santa Cruz, 1156 High Street, Santa Cruz, CA 95064, USA; University of California Santa Cruz Genomics Institute, University of California Santa Cruz, 1156 High Street, Santa Cruz, CA 95064, USA; Department of Biomolecular Engineering, University of California Santa Cruz, 1156 High Street, Santa Cruz, CA 95064, USA; University of California Santa Cruz Genomics Institute, University of California Santa Cruz, 1156 High Street, Santa Cruz, CA 95064, USA; Howard Hughes Medical Institute, 1156 High Street, Santa Cruz, CA 95064, USA; Department of Biomolecular Engineering, University of California Santa Cruz, 1156 High Street, Santa Cruz, CA 95064, USA; University of California Santa Cruz Genomics Institute, University of California Santa Cruz, 1156 High Street, Santa Cruz, CA 95064, USA; Howard Hughes Medical Institute, 1156 High Street, Santa Cruz, CA 95064, USA; University of California Santa Cruz Genomics Institute, University of California Santa Cruz, 1156 High Street, Santa Cruz, CA 95064, USA; Department of Molecular, Cell and Developmental Biology, University of California Santa Cruz, 1156 High Street, Santa Cruz, CA 95064, USA

**Keywords:** glioma, H3K27M mutation, epithelial mesenchymal transition

## Abstract

**Background:**

Diffuse midline gliomas with histone H3 K27M (H3K27M) mutations occur in early childhood and are marked by an invasive phenotype and global decrease in H3K27me3, an epigenetic mark that regulates differentiation and development. H3K27M mutation timing and effect on early embryonic brain development are not fully characterized.

**Results:**

We analyzed multiple publicly available RNA sequencing datasets to identify differentially expressed genes between H3K27M and non-K27M pediatric gliomas. We found that genes involved in the epithelial-mesenchymal transition (EMT) were significantly overrepresented among differentially expressed genes. Overall, the expression of pre-EMT genes was increased in the H3K27M tumors as compared to non-K27M tumors, while the expression of post-EMT genes was decreased. We hypothesized that H3K27M may contribute to gliomagenesis by stalling an EMT required for early brain development, and evaluated this hypothesis by using another publicly available dataset of single-cell and bulk RNA sequencing data from developing cerebral organoids. This analysis revealed similarities between H3K27M tumors and pre-EMT normal brain cells. Finally, a previously published single-cell RNA sequencing dataset of H3K27M and non-K27M gliomas revealed subgroups of cells at different stages of EMT. In particular, H3.1K27M tumors resemble a later EMT stage compared to H3.3K27M tumors.

**Conclusions:**

Our data analyses indicate that this mutation may be associated with a differentiation stall evident from the failure to proceed through the EMT-like developmental processes, and that H3K27M cells preferentially exist in a pre-EMT cell phenotype. This study demonstrates how novel biological insights could be derived from combined analysis of several previously published datasets, highlighting the importance of making genomic data available to the community in a timely manner.

## Background

Pediatric high-grade gliomas (pHGGs) are aggressive brain tumors occurring at a median age of 6 years [1]. Sixty percent of pHGGs harbor a histone H3 K27M (H3K27M) mutation, which is associated with an aggressive phenotype and dismal survival rates [2]. H3K27M-mutant pHGG tumors are located along the midline, including in the pons, cerebellum, and brainstem. A diffuse phenotype and delicate location leave them unsuitable for surgery, and their pronounced chemoresistance renders the standard treatments for gliomas ineffective, resulting in a median survival time of only 12 months [3, 4]. The prognostic significance of the H3K27M mutation in these gliomas resulted in a new World Health Organization tumor classification, diffuse midline glioma with H3K27M mutation [5].

The H3K27M mutation results in a global decrease in H3K27me3, an epigenetic repressive mark and posttranslational histone modification [6]. Seventy-five percent of gene loci lose or have reduced H3K27me3, although a few loci gain the mark as a result of the H3K27M mutation [2, 7]. H3K27me3 is deposited predominantly by EZH2, the catalytic subunit of the PRC2 methyltransferase complex. By regulating H3K27me3, EZH2 maintains cell identity and regulates cellular differentiation [8–11]. Silencing EZH2 in neuroepithelial cells before their differentiation alters the distribution of the progeny cell types [12]. EZH2 also maintains neuroepithelial cell integrity and midbrain identity [13, 14].

Because H3K27me3 is globally lost in H3K27M-mutant glioma, the subsequent deregulation of gene expression is thought to lead to tumorigenesis, although the developmental timing of the mutational event is important [15]. H3K27M expression in neural stem cells has led to tumorigenesis in mice when accompanied by *TP53* knockout and/or *PDGFRA* amplification, but this combination of molecular aberrations failed to result in tumorigenesis when introduced in mature astrocytes [16, 17]. However, the precise cell type of origin for H3K27M gliomas is not yet known. Candidate cell types include neuroepithelial cells (also known as neural stem cells), radial glia (also known as neural progenitor cells), and oligodendrocyte precursor cells (OPCs) [16–18].

Many important brain developmental processes are regulated by H3K27me3 deposition and could contribute to gliomagenesis if not well controlled. One of these is the epithelial-mesenchymal transition (EMT) pathway, which is essential for gastrulation, migration of neural crest cells, and neural tube formation [19–22]. The EMT is regulated by SNAI1, a transcription factor master regulator [23–25]. By regulating EMT, SNAI1 plays a critical role in many developmental processes, including gastrulation and differentiation of embryonic stem cells [26–28]. SNAI1 induces EMT through direct recruitment of PRC2, resulting in H3K27 trimethylation of key epithelial genes, as well as concurrently upregulating mesenchymal genes [29, 30].

In the brain, cellular transitions driven by EMT-like transcriptional programs are involved in key developmental steps such as the differentiation of neuroepithelial cells to both neuronal and glial cells [31, 32]. These transitional transcriptional programs, which control cell fate and identity in early neural cell development, are regulated by EZH2 [33].

Given the regulation of EMT-associated gene transcription by H3K27me3 deposition in the brain, and the disruption of this deposition by the H3K27M mutation, we sought to investigate EMT-related gene expression in pHGGs with and without the H3K27M mutation. We analyzed RNA sequencing (RNA-seq) data from 78 pHGGs obtained from several different studies (Supplementary Table S1). First, we performed differential expression analysis using RNA-seq–derived gene expression from bulk tumor samples and found that H3K27M gliomas differentially express pre-EMT genes [34]. Second, we examined previously published cerebral organoid data and observed transcriptional similarities between pretransition neural stem cells and H3K27M gliomas [35]. Finally, we leveraged a recent single-cell RNA-seq dataset to uncover multiple EMT-related transcriptional states in H3K27M tumor cells [18]. Overall, our results suggest that the H3K27M mutation may cause an arrest in development of a neural stem cell type due to lack of H3K27me3 transcriptional control of EMT-related cellular transitions, indicating a developmental window of opportunity for H3K27M mutations to induce gliomagenesis.

Our study highlights the importance of genomic data sharing for rare diseases, such as pHGGs. By combining RNA-seq data from multiple previously published studies, we were able to assemble a cohort of 78 pHGGs, large enough for the differential expression analysis of pHGGs with and without the H3K27M mutation. We used this new cohort of previously published data to derive a novel biological model to describe the molecular pathogenesis of the disease.

## Data Description

The RNA-seq data from bulk clinical pediatric glioma samples used in these analyses were downloaded from the Treehouse cancer compendium v8, which is publicly available at the Treehouse website [36]. All samples passed the RNA-seq quality control analysis used in the curation of the Treehouse cancer compendium [34]. The single-cell glioma RNA-seq data were downloaded from the Gene Expression Omnibus (accession: GSE102130), where they are publicly available. The dataset was log-normalized and filtered for low-expression and low-variability genes. The RNA-seq data from glioma cell lines were accessed with permission from dbGap phs000900.v1.p1, where they are available to other researchers with permission, and all samples passed the RNA-seq quality control analysis used in the curation of the Treehouse cancer compendium [34]. The bulk and single-cell organoid RNA-seq data were downloaded from the Gene Expression Omnibus (accession: GSE106245), which is publicly available. The datasets were log-normalized and filtered for low-expression and low-variability genes.

## Analyses

### Differential expression analysis of pediatric gliomas with and without H3K27M mutation reveals deregulation of genes involved in epithelial-mesenchymal transition

We obtained RNA-seq data from 33 H3K27M pediatric/young adult (ages 0–29 years) high-grade gliomas (pHGGs) and 45 non-K27M pHGGs from the Treehouse Childhood Cancer Initiative public cancer compendium v8 [36] (Supplementary Table S1). These data came from several cohorts including the Pacific Pediatric Neuro-Oncology Consortium (PNOC), Dr. Michelle Monje's studies, and The Cancer Genome Atlas [37–42].

**Figure 1.**
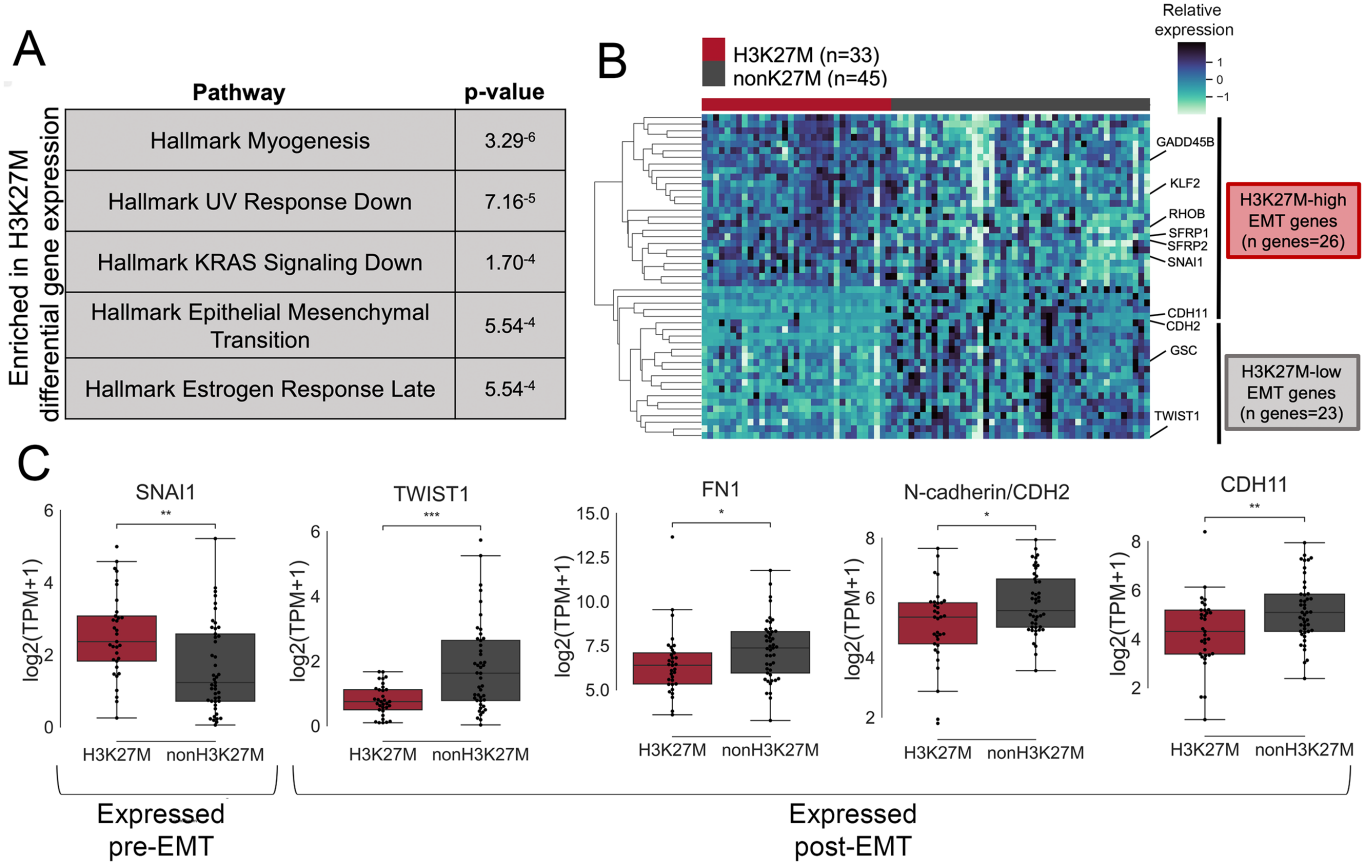
The EMT pathway is differentially expressed in H3K27M gliomas as compared with non-K27M gliomas. A, Differential expression analysis of a cohort of H3K27M and non-K27M pHGGs revealed significant enrichment of Hallmark Epithelial Mesenchymal Transition in genes overexpressed in H3K27M gliomas. B, Heat map of differentially expressed EMT genes between H3K27M and non-K27M pHGGs. C, *SNAI1* is overexpressed in H3K27M glioma, while *TWIST1, FN1, CDH2*, and *CDH11* are underexpressed in H3K27M glioma as compared with non-K27M gliomas (Mann-Whitney significance test; *P < 0.05, **P < 0.01, ***P < 0.001). Boxplot shows median and quartiles, whiskers are at 1.5 interquartile range.

Using the limma package in R [43], we conducted differential expression analysis between the H3K27M and non-K27M pHGG cohorts. A total of 1,905 genes are differentially expressed between the 2 tumor types (Supplementary Table S2). Using Gene Set Enrichment Analysis (GSEA) and the Molecular Signatures Database (MSigDB) [44], we found 23 biological signaling pathways with significant enrichment in protein-coding genes overexpressed in the H3K27M cohort (Supplementary Table S2). The top 5 most significantly enriched gene pathways included “Hallmark KRAS Signaling Down” (genes repressed by KRAS activation) and the “Hallmark Epithelial Mesenchymal Transition” (Fig. 1A). KRAS pathway enrichment is consistent with a recent study that found RAS signaling to be activated in H3K27M gliomas [45].

Because genes involved in the EMT are regulated by deposition of H3K27me3, an epigenetic transcriptional repressive mark that is lost in H3K27M cells, we were particularly interested in the differential expression of genes involved in the EMT pathway. The hallmark EMT pathway gene list is limited to 200 genes [46], so to comprehensively characterize expression of EMT-associated genes in H3K27M-mutant versus non-K27M tumors, we generated a master list of non-redundant EMT-related genes (n = 437) by merging several MSigDB developmental and cellular EMT-related gene sets (Supplementary Table S2). We included only genes from gene sets focused on EMT as a developmental process and eliminated gene sets that were derived from published studies of EMT in adult carcinomas as per MSigDB [46] because the epithelial nature of those cancers makes those gene sets inapplicable to pediatric gliomas. This list includes genes implicated in both pre- and post-EMT cell states, as well as intermediate EMT cell states and EMT-like processes.

To investigate differential EMT gene expression, we calculated the overlap between the EMT master list and the differentially expressed genes (Supplementary Table S2). We found 49 differentially expressed genes from the EMT master list, indicating potential differential activity of the EMT pathway in H3K27M-mutant gliomas (*P* < 7.89^−14^, hypergeometric test). Of these genes, 26 were more highly expressed in H3K27M tumors, and the remaining 23 were more highly expressed in non-K27M tumors (Fig. 1B). Further investigation via manual inspection revealed that, in general, the EMT-related genes overexpressed in the H3K27M cohort are associated with the transcriptional profile of cells prior to an EMT-like transition. In contrast, many of the EMT genes underexpressed in H3K27M tumors were associated with a post-EMT cell state.

To statistically quantify the association of the 26 EMT-related genes overexpressed in the H3K27M cohort with pre-EMT cell states in the brain, we manually identified 9 gene sets relating to epithelial cells and early brain development (Supplementary Table S2). The H3K27M-high EMT genes had significant enrichment in 8 of 9 gene sets (*P* < 0.1). In contrast, we calculated the enrichment of 26 randomly selected genes in these 9 gene sets and it was not significant (Supplementary Table S2). The enriched epithelial gene sets include “GO Epithelium Development” (*P* < 3.4^−12^, hypergeometric test), “GO Epithelial Cell Differentiation” (*P* < 3.587^−4^), and “GO Neural Tube Formation” (*P* < 0.001). In the developing brain, some of the cells of the neural tube, a pseudostratified epithelium, undergo an EMT in order to migrate [21]. *RHOB*, which plays a role in epithelial cell maintenance in the neural tube [47], is more highly expressed in H3K27M tumors and belongs to 3 of 9 epithelial gene sets. Additionally, *SFRP1* and *SFRP2*, which are crucial in neural tube formation [48], are more highly expressed in H3K27M tumors and belong to 6 of 9 epithelial gene sets.

Importantly, we noted that *SNAI1*, a transcription factor and key regulator of the EMT transcriptional program, is significantly overexpressed in H3K27M tumors (log_2_ fold-change [LFC] = 0.6; Fig. 1C). High expression of *SNAI1* is a marker of the beginning of the induction of EMT or EMT-like cellular transitions. If the transition is successful, this is followed by high expression of post-EMT markers *TWIST1* [49], fibronectin (*FN1*) [50], N-cadherin (*CDH2*) [51], and cadherin-11 (*CDH11*) [52]. Using a Mann-Whitney nonparametric significance test, we found significantly reduced expression of all of these genes in H3K27M tumors (*TWIST1* LFC = −1.2, *FN1* LFC = −0.2, *CDH2* LFC = −0.2, *CDH11* LFC = −0.3; Fig. 1C). *TWIST1, CDH2*, and *CDH11* are also underexpressed in the H3K27M cohort by the limma analysis.

Because SNAI1 induces EMT-like processes in the developing brain by directly recruiting PRC2 methyltransferase activity for H3K27-trimethylation, a process blocked by the H3K27M mutation, we hypothesized that the occurrence of the H3K27M mutation may promote tumorigenesis by stalling EMT during early neuroepithelial differentiation. To further investigate this hypothesis, we performed comparative RNA-seq expression outlier analysis developed by the Treehouse Childhood Cancer Initiative, which identifies genes with outlier expression in individual samples as compared to a background cohort of highly correlated and disease-matched samples (pan-disease analysis, see Methods) [34]. We identified genes with outlier expression only in non-K27M pHGG samples (but not H3K27M pHGG samples) as compared to a background glioma cohort and noted that 4 of the top 10 enriched pathways were related to EMT, including “TGF-Beta regulation of the extracellular matrix” (adjusted *P* = 4.01^−9^) and “Extracellular matrix organization” (adjusted *P* = 2.98^−5^) (Supplementary Fig. S1, Supplementary Table S2).

Finally, because EMT is associated with invasiveness in gliomas, and diffuse midline gliomas are by nature more invasive than hemispheric glioma, we performed an additional analysis restricted to diffuse intrinsic pontine glioma (DIPG) to elucidate the role of the H3K27M mutation in the observed EMT-related transcriptional profiles. The goal of this analysis was to remove any potential histological or location signal that may be influencing the EMT-related gene expression. We used 10 H3 wild-type DIPG samples and 47 H3K27M DIPG samples from Treehouse Cancer Compendium v11.

Limma differential expression analysis revealed 48 genes with higher expression in H3K27M DIPG compared with non-K27M DIPG (Supplementary Table S2). We again computed statistical overlap of these genes with 9 gene sets relating to epithelial cells and early brain development and found significant overlap with 4 of the 9 gene sets (*P* < 0.1, Supplementary Table S2). The enrichment of 48 randomly selected genes in these 9 gene sets was not significant (Supplementary Table S2).

Overall, our multiple analyses of the pHGG RNA-seq cohort suggest that H3K27M pHGG tumors are characterized by a transcriptional profile typically expressed by cells before undergoing an EMT-like transitional process, while non-K27M pHGG tumors are characterized by post-EMT gene expression.

### H3K27M-mediated gliomagenesis is associated with pre-EMT cell types

**Figure 2.**
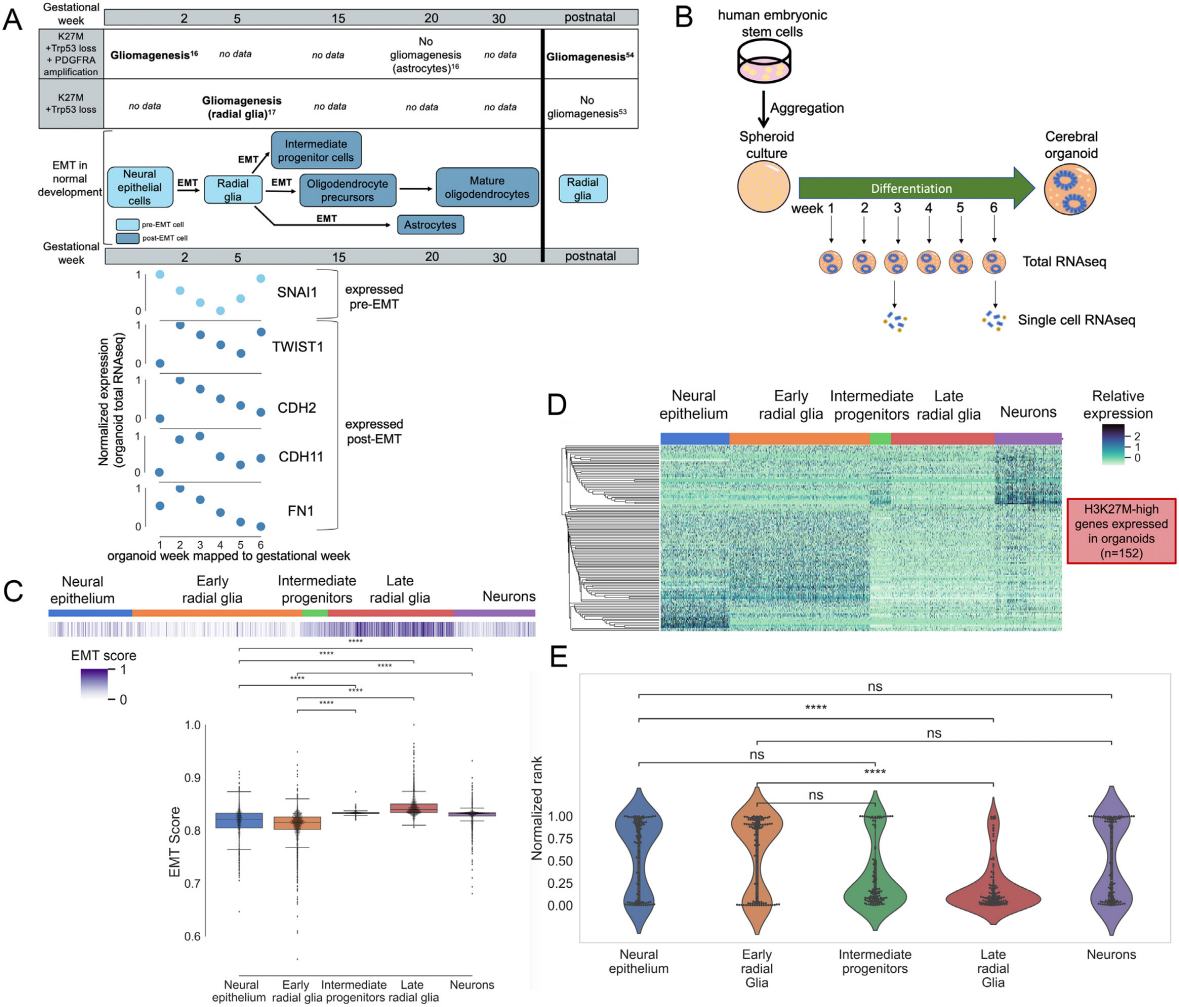
H3K27M-specific EMT transcriptional signature is similar to pre-EMT neural stem cell expression in cerebral organoids. A),*In vitro* and *in vivo* experimental H3K27M-associated gliomagenesis occurs exclusively in pre-EMT cell types (top). These cell types are represented in our cerebral organoid assay, and a time course of these organoid cultures represents 2 EMT events in early brain development (bottom). B, Experimental workflow for total RNA-seq and single-cell RNA-seq from a human embryonic stem cell–derived cerebral cortex organoid time course experiment. C, Single cells from cerebral organoids were scored for EMT completion. Pre-EMT neural epithelium and early radial glia were least enriched for the EMT score, while post-EMT intermediate progenitors, late radial glia, and neurons were the most enriched. Boxplot shows median and quartiles, whiskers are at 1.5 interquartile range. D, A signature of genes differentially expressed in H3K27M gliomas and expressed in cerebral organoids shows highest expression in pre-EMT neural epithelium and early radial glia. E, EMT-related genes highly expressed in H3K27M-mutant gliomas are also highly expressed in neural epithelium and early radial glia (Mann-Whitney significance test; *P value < 0.05, **P value < 0.01, ^****^P value < 0.0001). The violin plots show a kernel density estimation of the data distribution.

Consistent with our differential expression analysis, a review of the literature revealed that H3K27M-associated gliomagenesis has been experimentally recapitulated only in cell types that are poised to undergo an EMT differentiation event (Fig. 2A). For example, a combination of H3K27M, *p53* loss, and *PDGFRA* constitutive activation in human neural progenitor cells (NPCs) induced low-grade gliomas when injected into the pons of neonatal mice [16]. These gliomas expressed markers of pre-EMT neuroepithelial cells. Another study found that H3K27M and *Trp53* loss was sufficient for gliomagenesis in the NPCs of embryonic mice in the forebrain and hindbrain [17]. Strikingly, when introduced postnatally, H3K27M and *p53* loss in pre-EMT NPCs was not sufficient for gliomagenesis, although postnatal induction of H3K27M, *Trp53* loss, and *PDGFRA* amplification in pre-EMT NPCs resulted in glioma formation [53, 54]. Additionally, no tumorigenesis was observed upon introduction of H3K27M, *p53* loss, and *PDGFRA* constitutive activation in mature astrocytes, a post-EMT cell type [16]. These observations indicate that experimental H3K27M-mediated gliomagenesis occurs in a pre-EMT cell type.

On the basis of our gene expression analysis and review of the literature, we hypothesized that H3K27M gliomas arise in pre-EMT cell types and retain the EMT-related transcriptional profile of the cell type in which the mutation arises. To compare the expression of the EMT-related genes of interest between H3K27M tumors and normal developing brain cells, we examined total and single-cell RNA-seq data from a human embryonic stem cell–derived cerebral cortex organoid time course experiment (Fig. 2B) [35]. These organoid cultures mimic the early weeks of human prenatal cortical development and generate relevant cell types, uniquely allowing us to investigate early time points in development that are not available in existing human fetal brain datasets. After induction of neural epithelium by week 1, at week 2 radial glia cells and Cajal-Retzius neurons are present, in addition to some remaining neuroepithelial cells. By week 5, the organoids contain populations of radial glia, intermediate progenitors, and deep-layer neurons.

When we investigated EMT-related gene expression in cerebral organoids during gestational weeks 1–6, we noted the presence of 2 EMT-related transcriptional transitions (Fig. 2A, bottom). The first transition starts as *SNAI1* expression peaks in neural stem cells (week 1), coincident with low expression of post-EMT markers *TWIST1, CDH2, CDH11*, and *FN1*. As differentiation from neural epithelial cells to early radial glia occurs, *SNAI1* expression decreases while post-EMT marker expression increases. In the second transition, as radial glia cells differentiate intermediate progenitor cells, *SNAI1* expression increases once again.

To further characterize the EMT-like transcriptional profiles represented in cerebral organoids, we utilized single-cell RNA-seq data from the cerebral organoids at gestational weeks 3 and 6 [35]. These sample collection times effectively covered all relevant cell type diversity because gestation week 3 organoids contain substantial populations of neural epithelial cells, early radial glia cells, and Cajal-Retzius neurons, while week 6 organoids are composed of late radial glia cells, intermediate progenitors, and immature neurons. We scored the EMT status of each cell using a gene signature representing EMT completion and a previously published scoring method based on aggregate expression of the gene set as compared with a control gene set (Fig. 2C, Supplementary Table S3, see Methods) [18, 55–58]. Neural epithelial and early radial glia cells show significantly lower EMT scores than post-EMT intermediate progenitors, late radial glia, and neurons (Mann-Whitney test, *P* < 0.0001). This shows that our assay contains distinct populations of pre- and post-EMT cerebral cells and is consistent with the levels of *SNAI1, CDH2, CDH11, FN1*, and *TWIST1* in the bulk weeks 1–6 organoid data. This dataset enables us to investigate transcriptional similarities between H3K27M-mutant gliomas and normal pre-EMT cell types during neural development.

We then examined the expression of genes overexpressed in H3K27M gliomas in the single-cell organoid RNA-seq dataset to see which normal cell type is most similar to H3K27M glioma cells. Of the 1,180 H3K27M-overexpressed genes, 152 genes passed the single-cell RNA-seq expression filter (Supplementary Table S3, see Methods). Hierarchical clustering of the expression profiles of these genes in normal cell types during neural development revealed highest expression in pre-EMT neural epithelium and early radial glia (Fig. 2D). We then ranked this gene signature on the basis of each gene's expression in each cell type (see Methods). We found that this signature is ranked most highly in pre-EMT neural epithelium and in early radial glia (*P* < 0.05, Fig. 2E).

Overall, these results suggest that the differential EMT-related gene expression observed in our tumor cohort is consistent with identifiable stages in cyclic EMT-like transcriptional programs in the normal developing brain and that H3K27M tumor cells resemble normal developing brain cells at a point where they are expressing a pre-EMT transcriptional profile.

### Single-cell profiling of H3K27M gliomas reveals groups of cells with different EMT-related transcriptional profiles

We used recently published single-cell RNA-seq data from 6 H3K27M and 2 H3 wild-type (H3WT) gliomas to directly investigate the EMT-related transcriptional profiles of single-cell populations within each tumor type [18]. One of the H3K27M tumors harbors the mutation in the *HIST1H3B* gene (referenced as H3.1K27M), while the remaining 5 H3K27M tumors harbor the mutation in the *H3F3A* gene (referenced as H3.3K27M).

**Figure 3.**
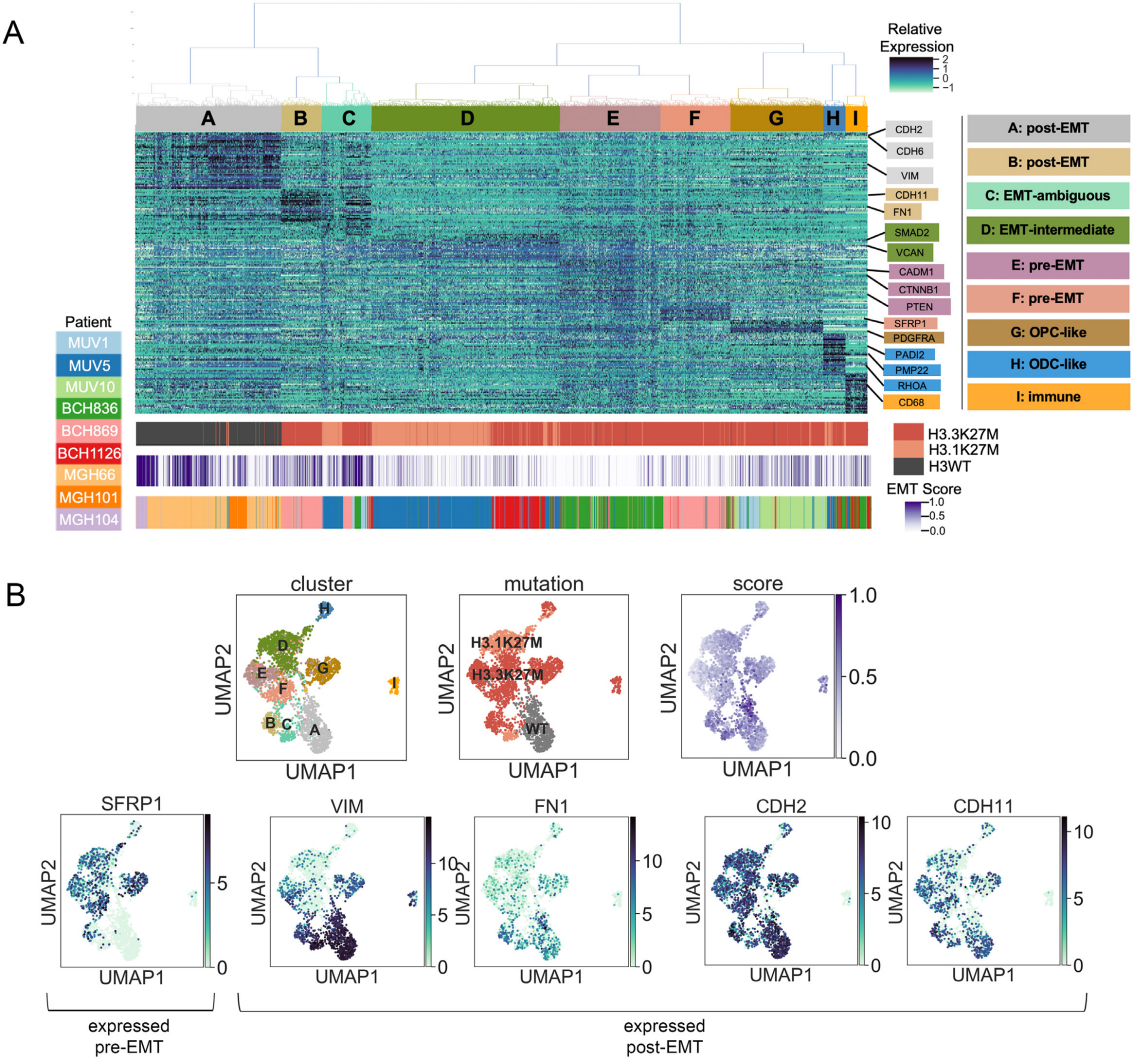
Single-cell RNA sequencing of H3K27M and non-K27M gliomas reveals multiple EMT stages within tumors. A, Expression heat map showing hierarchical clustering of 3,057 cells from 6 H3K27M and 2 non-K27M high-grade gliomas, with a master list of EMT genes. Nine clusters (A–I) were assigned gene signatures on the basis of maximum mean gene expression in each cluster, and clusters were classified on the basis of manual review of each gene signature. Histone H3 mutation status and EMT score are shown at the bottom of the heat map (ODC: oligodendrocyte; OPC: oligodendrocyte precursor). B, UMAP dimensionality reduction projection of the same expression data as the heat map and labeled by cluster, histone H3 mutation status, and EMT score. Expression of selected pre-EMT and post-EMT genes is shown in the bottom panel.

We performed hierarchical clustering of 3,057 tumor cells using 207 genes from the EMT master list that passed expression filters (see Methods, Supplementary Table S4) [59]. Nine EMT-related clusters were discovered and named A–I (Fig. 3A, Supplementary Table S4). Cluster gene signatures were identified by assigning each cluster the genes with maximum mean expression in that cell cluster across the dataset (Supplementary Table S4).

We assigned cluster function on the basis of manual review of genes in each signature, and observed several populations of cells whose presence in this dataset has already been noted [18]. Cluster I is composed predominantly of non-malignant immune cells, indicated by comparatively high expression of immune markers such as *CD68* [60]. Cluster H resembles oligodendrocytic cells, with highest expression of *PADI2, PMP22*, and *RHOA*, and Cluster G resembles oligodendrocyte precursor cells with the highest expression of *PDGFRA* [61–64]. The presence of each of these cell types has already been noted in H3K27M gliomas [18].

However, the remaining clusters are defined by genes associated with EMT. We again scored the EMT status of each cell with a gene signature representing EMT completion (Fig. 3A, see Methods) [18, 55–58]. Clusters D, E, and F scored the lowest overall, while Clusters A, B, and C scored the highest overall. Cluster relationships are shown with Uniform Manifold Approximation and Projection (UMAP) in Fig. 3B, and expression patterns of selected genes relating to transcriptional stages of EMT-like transitions are shown in the lower panel of Fig. 3B. Of the genes identified in the bulk RNA-seq analysis (Fig. 1C), only *FN1, CDH2*, and *CDH11* were expressed in the glioma single-cell RNA-seq data, so we also visualized *VIM* as a post-EMT marker and *SFRP1* as a pre-EMT marker.

In keeping with our previous analysis, we noted that Cluster A, which is composed mainly of H3WT glioma cells, strongly resembles post-EMT cells and most highly expresses canonical post-EMT markers including *CDH2, CDH6*, and *VIM* [65, 66]. This is consistent with our observation that non-K27M gliomas transcriptionally resemble a post-EMT state as compared to H3K27M in the bulk RNA-seq pHGG cohort.

Interestingly, within the clusters composed predominantly of H3K27M cells, we observed multiple EMT-related transcriptional profiles. Cluster B, composed of H3K27M cells, had highest expression of post-EMT markers including *CDH11* and *FN1*, potentially indicating a subclonal population of cells that differentiated through alternative means. Thus, we defined Clusters A and B “post-EMT.”

In contrast, H3K27M-expressing Clusters E and F cells exhibit comparatively the highest expression of several genes known for their expression in pre-EMT cell types, including *CADM1, PTEN, CTNNB1*, and *SFRP1* [48, 67–70]. Therefore, we defined Clusters E and F “pre-EMT.” In contrast, Cluster C has comparatively the highest expression of only 10 genes and has no clear expression profile of any stage of EMT, so we defined Cluster C “EMT-ambiguous.”

Cluster D was defined “EMT-intermediate” because it displays high expression of genes normally expressed while the EMT process is taking place, without a clear bias towards epithelial or mesenchymal gene expression, including *SMAD2* and *VCAN*, which are activated during the EMT process rather than before or after [71, 72].

### Histone H3.1K27M glioma cells express a different EMT-related transcriptional profile than H3.3K27M glioma cells

Further examination revealed that cluster D mainly consists of cells from the H3.1K27M-mutant tumor. H3.1 and H3.3K27M characterize 2 functionally different subtypes of H3K27M gliomas; H3.1K27M gliomas are comparatively rare but have a slightly better prognosis [40, 73]. Normally, histone H3.3 is preferentially located at active chromatin [74–76]. This leads to distinct patterns of epigenetic reprogramming in each histone variant, where loss of the H3.3K27me3 mark is directly correlated with areas of H3.3 genomic enrichment, while H3.1K27me3 loss is higher at intergenic regions [76, 77]. Because the H3K27M mutation is known to induce dose-dependent inhibition of PRC2 methyltransferase, this suggests that the localized distribution of histone H3.3 may result in higher local inhibition of PRC2 and loss of H3K27me3 at H3.3K27M sites [53, 76]. Because precise control of gene transcription via active chromatin is necessary for EMT-like developmental cell state transitions, a H3.3K27M mutation would be particularly damaging to proper regulation of these processes. Indeed, functional analysis of enhancer regions in H3.3K27M-expressing NPCs revealed enrichment of regions positively regulating EMT-related genes, indicating that H3.3 active chromatin regions are directly involved in transcriptional control of EMT-related genes [76]. This suggests that EMT-poised H3.3K27M cells will be unable to properly complete the transition owing to lack of transcriptional control.

Accordingly, we observed EMT-intermediate or E/M hybrid expression genes in glioma single-cell Cluster D, which has a substantial number of H3.1K27M glioma cells. We hypothesized that H3.1K27M cells may be more differentiated than H3.3K27M cells.

**Figure 4.**
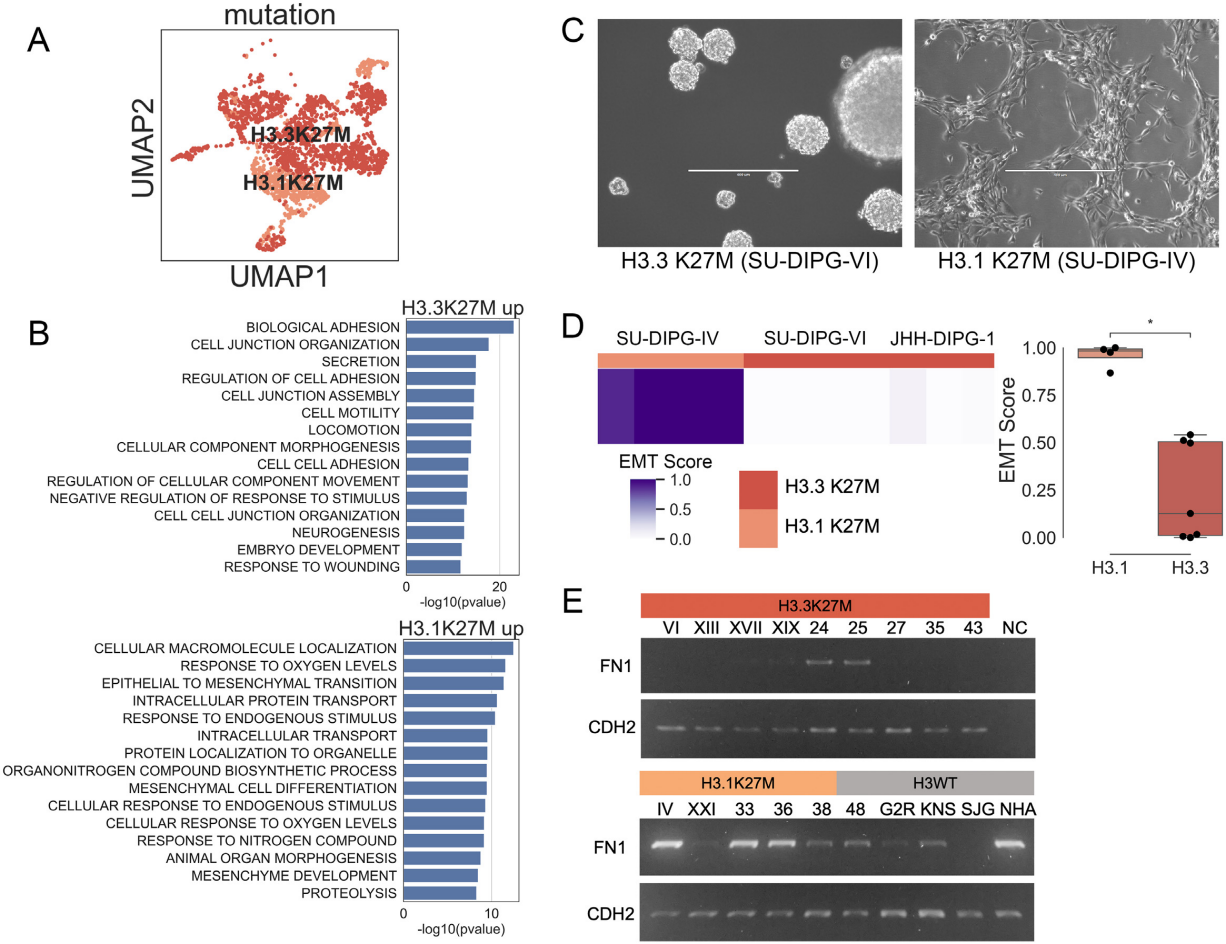
H3.1K27M glioma cells express a different EMT-related transcriptional profile than H3.3K27M glioma cells. A, UMAP dimensionality reduction of 2,458 histone-mutant glioma single cells. B, Gene set enrichment analysis of genes overexpressed in H3.3K27M vs H3.1K27M (top) or H3.1K27M vs H3.3K27M (bottom) by Wilcoxon rank-sum test using glioma single-cell RNA-seq data. C, Representative images of H3.1K27M and H3.3K27M glioma–derived cell cultures. Scale bar 400 μm. D, Total RNA sequencing datasets from glioma cell lines were scored for EMT completeness (4 samples from SU-DIPG-IV, 4 samples from SU-DIPG-VI, and 3 samples from JHH-DIPG1). Scoring is shown in a heat map and a box plot (Mann-Whitney significance test; *P < 0.05). E, RT-PCR of *FN1* and *CDH2* expression in glioma primary cell cultures (all numbered lines are SU-DIPG).

To investigate this hypothesis further, we subset the single-cell glioma RNA-seq data to 2,458 cells with H3.1K27M or H3.3K27M mutation and performed the Wilcoxon rank-sum test to identify genes overexpressed in each variant group (Supplementary Table S4, Supplementary Fig. S2). Consistent with our previous observations, GSEA of Gene Ontology (GO) gene sets (Fig.   4B, Supplementary Table S4) revealed enrichment of epithelial gene sets in H3.3K27M compared with H3.1K27M (GO Adhesion pathways, GO Neurogenesis, GO Embryo Development) and mesenchymal gene sets in H3.1K27M compared with H3.3K27M (GO EMT pathway, GO Mesenchymal Cell Differentiation, and GO Mesenchyme Development). Additionally, scoring of all cells for EMT completeness shows that H3.1K27M cells score significantly higher overall than H3.3K27M cells, while non-K27M cells score significantly higher than either mutant cell type (Supplementary Fig. S3). However, because the H3.1K27M cells come from a single tumor, we performed additional analysis to investigate this observation.

We cultured DIPG primary cell lines isolated in a previous study to investigate the expression of EMT markers in H3.3K27M, H3.1K27M, and non-K27M glioma cells [78]. Morphologically, we observed that when cultured in serum-free conditions, the H3.1K27M cell lines preferentially grow attached to the flask (4 of 5 cell lines), while the H3.3K27M cells preferentially grow as neurospheres (8 of 9 cell lines) (Fig. 4C). Because differentiation out of the neurosphere state is accompanied by attachment and increased expression of N-cadherin, this morphological trend is consistent with our hypothesis that H3.1K27M cells exist in a more differentiated state than H3.3K27M cells [79].

We analyzed RNA-seq data from 3 DIPG cell lines to compare the expression of EMT genes (SU-DIPG-IV is H3.1K27M mutant; SU-DIPG-VI and JHH-DIPG1 are H3.3K27M mutant). We used 4 replicate samples from each of SU-DIPG-IV and SU-DIPG-VI and 3 replicate samples from JHH-DIPG1. Each sample was scored using a gene signature of EMT completion (see Methods), and the H3.1K27M samples scored significantly higher than the H3.3K27M samples (Fig. 4D, *P* < 0.05).

We then performed RT-PCR to quantify the expression of *FN1* and *CDH2*, canonical post-EMT genes that were previously identified as differentially expressed by Mann-Whitney test in the bulk glioma RNA-seq analysis (Fig. 4E, full-length gel in Supplementary Fig. S4). We attempted to quantify E-cadherin/*CDH1* becuse it is a canonical pre-EMT marker, but the levels were so low as to be undetectable by RT-PCR in these cell lines [RNA-seq < 1.0 log_2_(TPM + 1)]. We compared 9 H3.3K27M cell lines (SU-DIPG-VI, XIII, XVII, XIX, 24, 25, 27, 35, and 43) with 5 H3.1K27M cell lines (SU-DIPG-IV, XXI, 33, 36, and 38) and included 5 H3 wild-type lines (SU-DIPG-48, pcGBM2R, KNS42, SJ-GBM2, and normal human astrocytes hTERT) and a negative RT-PCR control (NC). Overall, the H3 wild-type and H3.1K27M cell lines appear to more highly express both post-EMT markers, in keeping with the bulk and single-cell RNA-seq analyses. Our computational and *in vitro* observations are consistent with a recent study indicating that H3.1K27M tumor cells are overall more differentiated than H3.3K27M tumor cells [76]. Our results are also consistent with previous studies on EMT in pediatric gliomas, which first found a mesenchymal subtype of DIPG and subsequently discovered that H3.1K27M-mutant gliomas express genes associated with a more mesenchymal subtype of glioblastoma [73, 80].

Overall, these data suggest that the histone H3K27M mutation is associated with a preferentially early or pre-EMT cell state as compared with non-K27M cells but that H3.1K27M cells may represent a somewhat later or intermediate-EMT cell state as compared with H3.3K27M cells.

## Discussion

H3K27M diffuse midline gliomas are aggressive tumors generally occurring in early childhood in the hindbrain or midline. These tumors have poor prognosis and do not respond to standard chemotherapies for adult gliomas [81]. In contrast to most adult cancers, pediatric cancers, including pediatric gliomas, are thought to occur due to a developmental stall relating to epigenetic dysregulation of normal cellular differentiation pathways [15, 40, 82]. H3K27M diffuse midline glioma cells lose EZH2-deposited H3K27me3 epigenetic transcriptional control markers, which are known to play crucial roles in cell differentiation and development in the brain [6]. In particular, normal H3K27me3 deposition controls neural cell differentiation through multiple EMT processes [22]. Research has implicated the EMT in pediatric gliomas [80, 83, 84], particularly those with a more invasive phenotype. Histone deacetylase inhibitor treatment of *in vitro* pHGG cells reversed mesenchymal phenotypes, in keeping with a model in which the interplay between H3K27 acetylation and methylation controls EMT-related transcriptional states [38]. We hypothesized that loss of H3K27me3 in H3K27M-mutant gliomas may lead to a stall in EMT processes in normal brain development.

In this study, we observed that various canonical EMT-inducing genes are significantly overexpressed in H3K27M-mutant pHGGs, compared with non-K27M pHGGs, while many canonical mesenchymal markers are underexpressed in H3K27M pHGGs as compared with the non-K27M tumors. In particular, we noted higher expression of the pre-EMT transcription factor *SNAI1* in H3K27M-mutant gliomas. Because *SNAI1* relies on PRC2 and H3K27me3 to facilitate EMT through gene expression regulation, this may indicate an arrest in the EMT process. The existence of a hybrid epithelial/mesenchymal phenotype is well established: the result of a partial EMT is the expression of both epithelial and mesenchymal genes [85]. Studies have shown that a hybrid E/M phenotype may indicate a worse prognosis than mesenchymal-only states in solid tumors [85–87].

We hypothesized that if H3K27M mutation prevents full EMT, neural stem cells harboring H3K27M may be forced to retain a proliferative, stem cell phenotype, eventually leading to tumorigenic development. Accordingly, we observed from extensive literature review that experimental induction of H3K27M-associated gliomas has occurred exclusively in pre-EMT cell types, and that 2 consecutive EMT-like transcriptional transitions occur early in normal brain development.

Single-cell RNA-seq from H3K27M and non-K27M tumors confirmed a post-EMT expression signature in the non-K27M cells and also revealed subsets of H3K27M cells with different EMT-related transcriptional profiles. Specifically, we observed an intermediate EMT signature in the H3.1K27M cells as compared with the H3.3K27M cells. This was also observed in bulk RNA-seq and *in vitro* RT-PCR analysis. We hypothesize that because the H3.1K27M mutation is not concentrated at active chromatin, it has less repressive power as specific developmental processes such as EMT are activated over time. If a subset of H3.1K27M cells are able to differentiate, this may explain why H3.1K27M gliomas have a slightly better prognosis.

**Figure 5.**
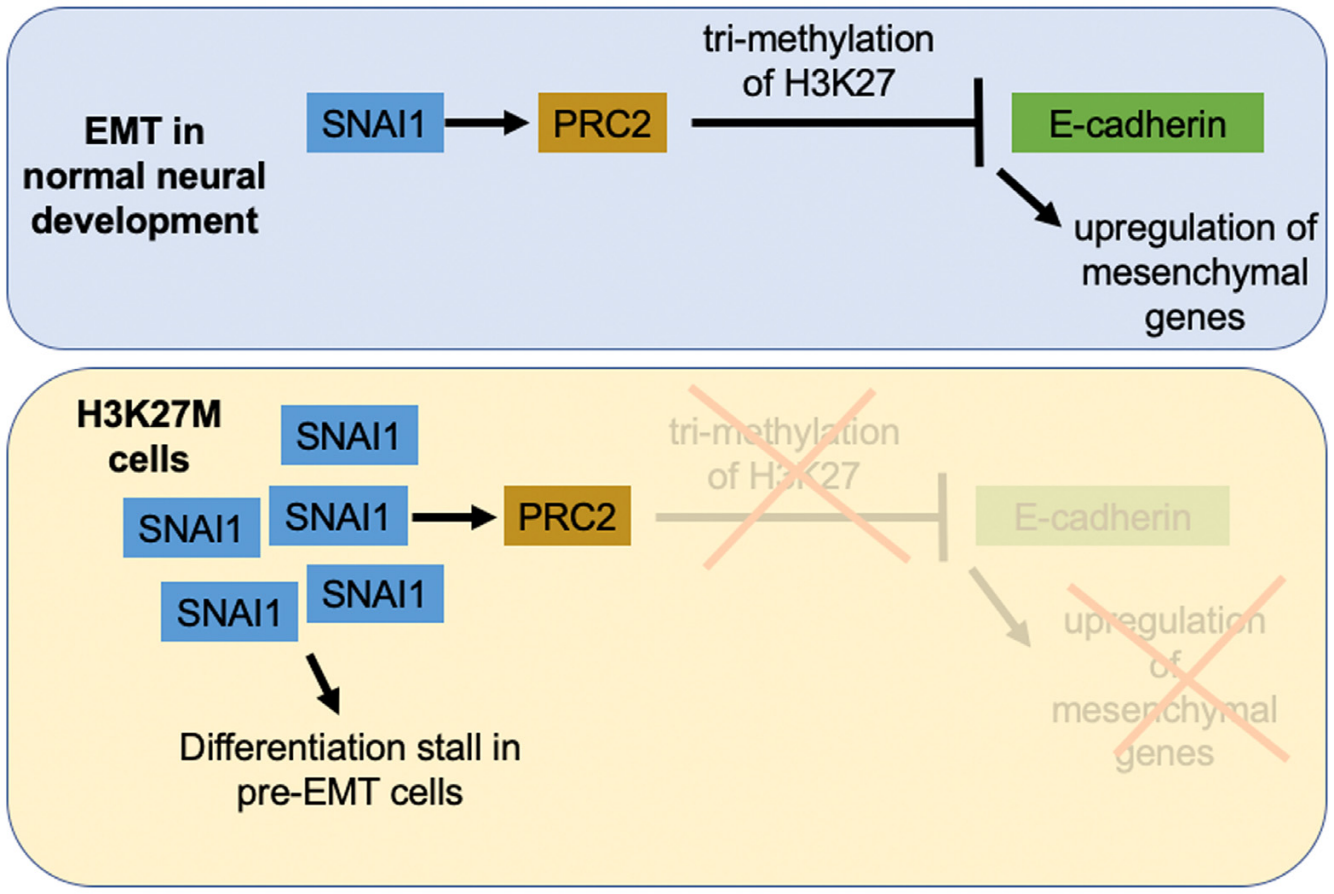
Proposed model for EMT stall in H3K27M cells. We propose that H3K27M cells retain high levels of SNAI1 expression but remain stalled in a pre-EMT state owing to inability of PCR2 to tri-methylate H3K27.

To conclude, we mined 3 publicly available RNA-seq datasets from pediatric gliomas and cerebral organoids to generate a hypothesis for the gliomagenesis of H3K27M gliomas. We propose that the H3K27M mutation is tumorigenic when the mutational hit occurs in a cell poised to undergo an EMT-like cell state transition, due to the dependence of EMT-associated transcriptional activity on the correct timing of the H3K27me3 mark (Fig. 5). More work is needed to characterize the observed difference in the EMT-associated transcriptional profiles between the H3.1 and H3.3K27M variants. Additionally, a limitation of our study is that it is difficult to isolate the role of the H3K27M mutation from other factors such as histology and tumor location. Future studies will focus on EMT-related transcriptional programs in cellular models with inducible H3K27M expression to further characterize the molecular interplay between the H3K27M mutation and EMT in developing brain cells.

Taken together, our results hold important implications for better understanding the developmental origin and timing of these aggressive and untreatable cancers. Furthermore, the presence of an epigenetically driven differentiation stall may imply that a pharmacological methylation agent or a pro-differentiation therapy may aid in future treatment of H3K27M-mutant tumors [88].

## Potential Implications

Our study holds implications for other diseases because H3K27M mutation is not exclusive to diffuse midline gliomas. It can also be found in a fraction of pediatric ependymomas and medulloblastomas [89]. Interestingly, ependymomas located in the posterior fossa typically do not harbor the H3K27M mutation but exhibit the K27M-associated H3K27 hypomethylation phenotype. Thus, the proposed differentiation stall and an associated EMT transcriptional signature as a result of H3K27me3 loss may also apply to these cancers. Beyond the SNAI1-H3K27me3 axis, EMT is also regulated by other epigenetic marks [90]. Given the epigenetically dysfunctional nature of many pediatric cancers [15], EMT arrest could conceivably play a role in the oncogenesis of these tumors as well.

## Methods

### Glioma bulk RNA sequencing data

Gene expression data from 78 pHGG samples were downloaded from the Treehouse Childhood Cancer Initiative public compendium v8 (Tumor Compendium v8 Public) [36]. All samples in the compendium have been uniformly processed using the UC Santa Cruz TOIL RNA-seq pipeline (v3.3.4) [91]. This dataset (n = 58,581 genes) is in transcripts per million (TPM) and normalized by log_2_(TPM + 1). We divided the dataset into 33 H3K27M-mutant samples and 45 non-K27M samples and performed differential expression analysis of all genes between the 2 groups using R library limma v3.34.9 in R v3.3.4. We performed GSEA of the resulting 1,905 differentially expressed genes (*P* < 0.1) with MSigDB v7.0 on the GSEA/MSigDB website v6.4 (Supplementary Table 2). Because the EMT pathway was in the top 5 most significantly enriched pathways in H3K27M overexpressed genes, we created a non-redundant master list of EMT genes (n = 437) by merging 7 EMT-related MSigDB pathways and by identifying EMT-related genes through manual literature curation (Supplementary Table 2).

We performed pan-disease outlier analysis on all the pHGG samples using Treehouse CARE (see Availability of Source Code and Requirements section) against the Treehouse Cancer Compendium v10. Pan-disease outlier analysis identifies genes with outlier expression in each sample of interest as compared to a background cohort of tumors identified as most similar (in this analysis, the background cohort was 37 pediatric gliomas, 19 young adult gliomas, 18 pediatric glioblastomas, and 4 young adult glioblastomas) [34]. We identified a list of genes with outlier expression in the non-K27M pHGG samples that did not also have outlier expression in the H3K27M pHGG samples, and performed GSEA using Enrichr in the GSEApy package (gseapy-v0.9.17) [92] against the BioPlanet_2019 library with *P*-value cut-off 0.05 (outlier genes and enriched pathways in Supplementary Table 2). We used the EnrichmentMap app in Cytoscape to visualize functionally similar clusters of enriched pathways [93].

### Cerebral organoid RNA sequencing data (bulk and single cell)

Gene expression data (TPM) from 6 weekly time points of human cerebral organoid growth were downloaded from accession GSE106245 [35]. Organoid weeks 0–5 were converted to gestational weeks 1–6 and duplicate gene measurements were averaged. For Fig. 2A, expression of each gene was normalized in the range 0–1. Single-cell RNA-seq data from weeks 2 and 5 (gestational weeks 3 and 6) cerebral organoids were downloaded from accession GSE106245 [35]. Expression data were filtered to remove genes with expression in <10% of cells. Cell types were assigned using a list of marker genes (Supplementary Table S3).

### Glioma single-cell RNA sequencing data

Smart-seq2 RSEM TPM single-cell RNA-seq data from 3,057 glioma cells were downloaded from accession GSE102130 [18]. Data were log_2_-normalized and filtered to remove genes with expression in <20% of cells. The cells per tumor remaining after filtering are as follows: MUV1: 146, MUV5: 708, MUV10: 286, BCH836: 527, BCH869: 492, BCH1126: 299, MGH66: 442, MGH101: 92, MGH104: 65. Hierarchical clustering of all cells was performed using the Python scipy.cluster.hierarchy function (scipy v1.4.1) after subsetting to a non-redundant master list of EMT genes (n = 437, Supplementary Table S2). Of these genes, 207 passed the expression filter and were included in the hierarchical clustering. The clustering results were plotted using the scipy.cluster.hierarchy.dendrogram function with threshold set to 3.5. Gene signatures for each cluster were assigned by identifying the cluster in which each gene has maximum mean expression, and assigning that gene to that cluster. For UMAP visualizations, Leiden clustering was performed on the single-cell data using the scanpy.tl.leiden function (scanpy v1.4.5.post1) with resolution set to 0.5 and top 10 principal components used as input.

### DIPG cell lines

The patient-derived DIPG cell lines (SU-DIPG-IV, SU-DIPG-VI, SU-DIPG-XIII, SU-DIPG-XVII, SU-DIPG-XIX, SU-DIPG-XXI, SU-DIPG-24, SU-DIPG-25, SU-DIPG-27, SU-DIPG-33, SU-DIPG-35, SU-DIPG-36, SU-DIPG-38, SU-DIPG-48) were kindly provided by Dr. Michelle Monje (Stanford University School of Medicine, Stanford, CA) [38]. SU-DIPG-IV, SU-DIPG-XXI, SU-DIPG-33, SU-DIPG-36, and SU-DIPG-38 cells harbor a H3.1K27M mutation while SU-DIPG-VI, SU-DIPG-XIII, SU-DIPG-XVII, SU-DIPG-XIX, SU-DIPG-24, SU-DIPG-25, SU-DIPG-27, SU-DIPG-35, and SU-DIPG-43 cells harbor a H3.3K27M mutation. SU-DIPG-48 and glioblastoma cell line SU-pcGBM-2 are H3WT. Glioblastoma H3WT cell lines, as well as KNS-42 (BCRJ Cat# 0295, RRID:CVCL_0378), SJ-GBM2 (RRID:CVCL_M141), and 1 normal astrocyte cell line NHA hTERT, were kindly provided by Prof. Sameer Agnihotri (University of Pittsburgh Medical Center Children's Hospital of Pittsburgh, Pittsburgh, PA). The Universal Mycoplasma Detection Kit (ATTC, Manassas, VA) was used for testing SU-DIPG-XIII, XVII, XIX, and VI latest on 10 January 2020. All cells were cultured in tumor stem medium containing 50X B-27 Supplement Minus Vitamin A (Invitrogen, Waltham, MA), H-EGF at 20 ng/mL (Shenandoah Biotechnology, Warwick, PA), H-FGF-basic-154 at 20 ng/mL (Shenandoah Biotechnology, Warwick, PA), H-PDGF-AA at 10 ng/mL (Shenandoah Biotechnology, Warwick, PA), H-PDGF-BB at 10 ng/mL (Shenandoah Biotechnology, Warwick, PA), and 0.2% heparin solution at 2 μg/mL (STEMCELL Technologies, BC, Canada). All experiments used cells collected within 5 passages after thawing. The cells were passaged by the treatment of TrypLE (Gibco, Waltham, MA) and DNAse I (Worthington, Lakewood, NJ) rocking at 37°C for 5–15 minutes, then HBSS (Corning, Corning, NY) was added to deactivate TrypLE. The cells were transferred to new Nunc EasYFlask Cell Culture Flasks (ThermoFisher Scientific, Waltham, MA) and grown in tumor stem medium as previously described. The bulk RNA-seq data from lines SU-DIPG-VI, SU-DIPG-IV, and JHH-DIPG1 were obtained with permission from Dr. Michelle Monje from dbGap accession phs000900.v1.p1.

### RNA extraction and RT-PCR

Total RNA was extracted from cell pellets using the Quick-RNA Miniprep Kit (Zymo Research, Irvine, CA). Complementary DNA was synthesized from 1 μg of total RNA using Oligo(dT)20 primers and the SuperScript III First Strand Synthesis System (Invitrogen, Waltham, MA). PCR was performed using KAPA HiFi HotStart ReadyMixPCR Kit (KAPA Biosystems, Wilmington, MA), 50 ng of template DNA, and the appropriate primers and 27 PCR cycles and an annealing temperature of 64°C. *CDH2* primer sequences were as follows: forward: ggcttaatggtgattttgctcag; reverse: tccataccacaaacatcagcac. *FN1* primer sequences were as follows: forward: cttgaaccaacctacggatgac; reverse: tcccatcatcataacacgttgc. Primer oligos were purchased from Integrated DNA Technologies.

### Data Analysis

All statistical comparisons were performed with a 2-sided Mann-Whitney test, with measurements taken from distinct samples without assumption of normality, and Benjamini-Hochberg multiple testing correction was applied. Single-cell and bulk tumor samples were scored for EMT activity using a manually curated set of mesenchymal genes and a previously published scoring method based on aggregate expression of the gene set as compared to a control gene set (Supplementary Table S3) [18, 56, 94].

## Availability of Source Code and Requirements

Code for figures and data analysis: https://github.com/lauren-sanders/EMT-paper

Code for outlier analysis: https://github.com/UCSC-Treehouse/CARE/

License: Apache-2.0

Operating system: Platform independent

Programming languages: Python, R

Other requirements: Python 3.8 or higher, R 3.3 or higher

## Data Availability

All data used for this article are available at the following websites or accession numbers: publicly available: (i) bulk glioma RNA-seq: [36], (ii) cerebral organoid RNA-seq: GSE106245, (iii) glioma single-cell RNA-seq: GSE102130. Data available with permission for the glioma cell line RNA-seq data dbGap phs000900.v1.p1. All supporting data and materials are available in the *GigaScience* GigaDB database [95].

## Additional Files

Supplementary Figure S1. Comparative gene expression analysis shows enrichment of post-EMT and mesenchymal pathways in genes with outlier expression in H3WT pHGG tumors.

Supplementary Figure S2. Top 30 differentially expressed genes by Wilcoxon rank-sum test comparing H3.3K27M mutant glioma cells with H3.1K27M mutant glioma cells.

Supplementary Figure S3. Continuum of EMT completeness scores in glioma single cell RNA-seq data.

Supplementary Figure S4. Full-length RT-PCR gel images for FN1 and CDH2 quantification.

Supplementary Table S1. Patient characteristics.

Supplementary Table S2. Differentially expressed genes between H3K27M and H3WT gliomas.

Supplementary Table S3. Genes used for EMT scoring.

Supplementary Table S4. Glioma single-cell clusters and genes.

## Abbreviations

DIPG: diffuse intrinsic pontine glioma; E/M: epithelial/mesenchymal; EMT: epithelial-mesenchymal transition; GO: gene ontology; GSEA: gene set enrichment analysis; H3WT: histone 3 wild-type; LFC: log_2_ fold-change; MSigDB: Molecular Signatures Database; NPC: neural progenitor cell; ODC: oligodendrocyte cell; OPC: oligodendrocyte precursor cell; pHGG: pediatric high-grade glioma; PNOC: Pacific Pediatric Neuro-Oncology Consortium; TPM: transcripts per million; UMAP: Uniform Manifold Approximation and Projection; UCSC: University of California Santa Cruz.

## Ethics Statement

The protocols for the PNOC-003 trial, Dr. Michelle Monje's studies, Dr. Mariella Filbin's studies, The Cancer Genome Atlas, the Children's Brain Tumor Tissue Consortium, the International Cancer Genome Consortium, and the University of Michigan Clinical Sequencing Exploratory Research have been previously described [18, 37–42]. The UCSC Treehouse Childhood Cancer Initiative protocol was approved by the UCSC Institutional Review Board (No. HS2648) [34].

## Competing Interests

The authors declare that they have no competing interests.

## Funding

This study was funded by American Association for Cancer Research NextGen Grant for Transformative Cancer Research Award (O.M.V.), St Baldrick's Foundation Consortium Award and Emily Beazley Kures for Kids Fund Hero Award (D.H., O.M.V., S.R.S.), Alex's Lemonade Stand Foundation for Childhood Cancer Research, Unravel Pediatric Cancer, Team G Childhood Cancer Foundation, and Live for Others Foundation, The Schmidt Futures Foundation (D.H.), CIRM Shared Stem Cell Facilities (CL1-00506) award to UCSC. A.C. is supported by the T32GM133391 Training Program in Molecular, Cell, and Developmental Biology. D.H. is a Howard Hughes Medical Institute Investigator. O.M.V. holds the Colligan Presidential Chair in Pediatric Genomics.

## Authors' Contributions

Analysis and manuscript authorship: L.M.S. and A.C.

Single-cell organoid cell type gene ranking: L.S.

Experimental work: A.C., A.v.d.B., and M.C.

Treehouse cancer compendium and manuscript review: H.C.B., E.T.K., J.P., K.L., A.G.L., and I.B.

Funding, scientific oversight, and manuscript review: D.H., S.R.S., and O.M.V.

## Supplementary Material

giaa136_GIGA-D-20-00117_Original_Submission

giaa136_GIGA-D-20-00117_Revision_1

giaa136_GIGA-D-20-00117_Revision_2

giaa136_Response_to_Reviewer_Comments_Original_Submission

giaa136_Response_to_Reviewer_Comments_Revision_1

giaa136_Reviewer_1_Report_Original_Submission Alexandra Sexton Oates -- 5/20/2020 Reviewed

giaa136_Reviewer_2_Report_Original_Submission Jacques Grill -- 5/23/2020 Reviewed

giaa136_Supplemental_Files
