# Supplementary material for: Identification of a differentiation stall in epithelial mesenchymal transition in histone H3–mutant diffuse midline glioma
Source: Gigascience. 2020 Dec 15;9(12):giaa136. doi: 10.1093/gigascience/giaa136 (PMC7736793; doi:10.1093/gigascience/giaa136)
Supplement: giaa136_GIGA-D-20-00117_Original_Submission [file giaa136_giga-d-20-00117_original_submission.pdf]

GigaScience

Identification of a differentiation stall in epithelial mesenchymal transition in histone H3 mutant diffuse midline glioma

--Manuscript Draft--

**Manuscript Number:**	GIGA-D-20-00117
**Full Title:**	Identification of a differentiation stall in epithelial mesenchymal transition in histone H3 mutant diffuse midline glioma
**Article Type:**	Research
**Funding Information:**	American Association for Cancer Research — Dr Olena M Vaske<br>St. Baldrick's Foundation — Dr David Haussler<br>Emily Beazley Kures for Kids Fund — Dr David Haussler<br>Alex's Lemonade Stand Foundation for Childhood Cancer — Dr David Haussler<br>Unravel Pediatric Cancer — Dr David Haussler<br>Team G Childhood Cancer Foundation — Dr David Haussler<br>Live for Others Foundation — Dr David Haussler<br>Schmidt Futures Foundation — Dr David Haussler<br>California Institute for Regenerative Medicine — Dr David Haussler
**Abstract:**	Background<br>Diffuse midline gliomas with Histone H3 K27M (H3K27M) mutations occur in early childhood and are marked by an invasive phenotype and global decrease in H3K27me3, an epigenetic mark which regulates differentiation and development. H3K27M mutation timing and effect on early embryonic brain development are not fully characterized.<br>Results<br>We analyzed multiple publicly available RNA sequencing datasets to identify differentially expressed genes between H3K27M and nonK27M pediatric gliomas. We found that genes involved in the epithelial-mesenchymal transition (EMT) were significantly overrepresented among differentially expressed genes. Overall, the expression of pre-EMT genes was increased in the H3K27M tumors as compared to nonK27M tumors, while the expression of post-EMT genes was decreased. We hypothesized that H3K27M may contribute to gliomagenesis by stalling an EMT in early brain development, and evaluated this hypothesis by employing another publicly available dataset of single-cell and bulk RNA sequencing data from developing cerebral organoids. This analysis revealed similarities between H3K27M tumors and pre-EMT normal brain cells. Finally, a previously published single-cell RNA sequencing dataset of H3K27M and nonK27M gliomas revealed subgroups of cells at different stages of EMT. In particular, H3.1K27M tumors resemble a later EMT stage compared to H3.3K27M tumors.<br>Conclusions<br>Our data analyses indicate that this mutation may be associated with EMT arrest, and that H3K27M cells preferentially exist in a pre-EMT cell phenotype. This study demonstrates how novel biological insights could be derived from combined analysis of previously published datasets, highlighting the importance of making genomic data available to the community in a timely manner.
**Corresponding Author:**	Lauren Sanders<br>University of California, Santa Cruz<br>Santa Cruz, CA UNITED STATES
**Corresponding Author Secondary Information:**	
**Corresponding Author's Institution:**	University of California, Santa Cruz

Powered by Editorial Manager® and ProduXion Manager® from Aries Systems Corporation

Corresponding Author's Secondary Institution:	
First Author:	Lauren Sanders
First Author Secondary Information:	
Order of Authors:	Lauren Sanders
	Allison Cheney
	Lucas Seninge
	Anouk van den Bout
	Marissa Chen
	Holly C Beale
	Ellen Towle Kephart
	Jacob Pfeil
	Katrina Learned
	A Geoffrey Lyle
	Isabel Bjork
	David Haussler
	Sofie R Salama
	Olena M Vaske
Order of Authors Secondary Information:	
Additional Information:	
**Question**	**Response**
Are you submitting this manuscript to a special series or article collection?	No
**Experimental design and statistics**<br><br>Full details of the experimental design and statistical methods used should be given in the Methods section, as detailed in our Minimum Standards Reporting Checklist. Information essential to interpreting the data presented should be made available in the figure legends.<br><br>Have you included all the information requested in your manuscript?	Yes
**Resources**<br><br>A description of all resources used, including antibodies, cell lines, animals and software tools, with enough information to allow them to be uniquely	Yes

identified, should be included in the Methods section. Authors are strongly encouraged to cite Research Resource Identifiers (RRIDs) for antibodies, model organisms and tools, where possible.<br><br>Have you included the information requested as detailed in our Minimum Standards Reporting Checklist?	
**Availability of data and materials**<br><br>All datasets and code on which the conclusions of the paper rely must be either included in your submission or deposited in publicly available repositories (where available and ethically appropriate), referencing such data using a unique identifier in the references and in the “Availability of Data and Materials” section of your manuscript.<br><br>Have you have met the above requirement as detailed in our Minimum Standards Reporting Checklist?	Yes

Manuscript

Click here to download Manuscript EMT Paper Giga Science Submission.pdf

Identification of a differentiation stall in epithelial mesenchymal transition in histone H3 mutant diffuse midline glioma

Lauren M. Sanders[1,4*#], Allison Cheney[2#], Lucas Seninge[1,4], Anouk van den Bout[2,4], Marissa Chen[2,4], Holly C. Beale[2,4], Ellen Towle Kephart[4], Jacob Pfeil[1,4], Katrina Learned[4], A. Geoffrey Lyle[2,4], Isabel Bjork[4], David Haussler[1,3,4], Sofie R. Salama[1,3,4+], Olena M. Vaske[2,4+]

[1]Department of Biomolecular Engineering, [2]Department of Molecular, Cell and Developmental Biology, [3]Howard Hughes Medical Institute, [4]University of California Santa Cruz Genomics Institute, University of California Santa Cruz, 1156 High Street, Santa Cruz, CA, USA, 95064

*Corresponding author

[#]Co-first author

[+]Co-senior author

Author Email Addresses

Lauren M. Sanders lmsh@ucsc.edu; Allison Cheney archeney@ucsc.edu; Lucas Seninge lseninge@ucsc.edu; Anouk Van Den Bout anvanden@ucsc.edu; Marissa Chen marissaamberchen@gmail.com; Holly C. Beale hcbeale@ucsc.edu; Ellen Towle Kephart ekephart@ucsc.edu; Jacob Pfeil <jpfeil@ucsc.edu; Katrina Learned klearned@ucsc.edu; A. Geoffrey Lyle aglyle@ucsc.edu; Isabel Bjork ibjork@ucsc.edu; David Haussler haussler@ucsc.edu; Sofie R. Salama ssalama@ucsc.edu; Olena M. Vaske olena@ucsc.edu

Corresponding Author

Lauren M. Sanders
1156 High Street, 220 Sinsheimer Labs
University of California Santa Cruz
Santa Cruz, CA 95060 USA
phone: (530) 409 2174
lmsh@ucsc.edu

Abstract

Background

Diffuse midline gliomas with Histone H3 K27M (H3K27M) mutations occur in early childhood and are marked by an invasive phenotype and global decrease in H3K27me3, an epigenetic mark which regulates differentiation and development. H3K27M mutation timing and effect on early embryonic brain development are not fully characterized.

Results

We analyzed multiple publicly available RNA sequencing datasets to identify differentially expressed genes between H3K27M and nonK27M pediatric gliomas. We found that genes involved in the epithelial-mesenchymal transition (EMT) were significantly overrepresented among differentially expressed genes. Overall, the expression of pre-EMT genes was increased in the H3K27M tumors as compared to nonK27M tumors, while the expression of post-EMT genes was decreased. We hypothesized that H3K27M may contribute to gliomagenesis by stalling an EMT in early brain development, and evaluated this hypothesis by employing another publicly available dataset of single-cell and bulk RNA sequencing data from developing cerebral organoids. This analysis revealed similarities between H3K27M tumors and pre-EMT normal brain cells. Finally, a previously published single-cell RNA sequencing dataset of H3K27M and

nonK27M gliomas revealed subgroups of cells at different stages of EMT. In particular, H3.1K27M tumors resemble a later EMT stage compared to H3.3K27M tumors.

Conclusions

Our data analyses indicate that this mutation may be associated with EMT arrest, and that H3K27M cells preferentially exist in a pre-EMT cell phenotype. This study demonstrates how novel biological insights could be derived from combined analysis of previously published datasets, highlighting the importance of making genomic data available to the community in a timely manner.

Keywords

Glioma, H3K27M mutation, epithelial mesenchymal transition

Background

Pediatric high grade gliomas (pHGGs) are aggressive brain tumors occurring at a median age of 6[1]. Sixty percent of pHGGs harbor a histone H3 K27M mutation, which is associated with an aggressive phenotype and dismal survival rates[2]. H3K27M-mutant pHGG tumors are located along the midline, including in the pons, cerebellum, and brainstem. A diffuse phenotype and delicate location leave them unsuitable for surgery, and their pronounced chemoresistance renders the standard treatments for gliomas ineffective, resulting in a median survival time of only 12 months[3,4]. The prognostic significance of the H3 K27M mutation in these gliomas resulted in a new WHO tumor classification, diffuse midline glioma with H3K27M mutation[5].

The H3K27M mutation results in a global decrease in H3K27me3, an epigenetic repressive mark and posttranslational histone modification[6]. Seventy five percent of gene loci lose or have reduced H3K27me3, although a few loci gain the mark as a result of the H3K27M

mutation[2,7]. H3K27me3 is deposited predominantly by EZH2, the catalytic subunit of the PRC2 methyltransferase complex. By regulating H3K27me3, EZH2 maintains cell identity and regulates cellular differentiation[8–11]. Silencing EZH2 in neuroepithelial cells before their differentiation alters the distribution of the progeny cell types[12]. EZH2 also maintains neuroepithelial cell integrity, and midbrain identity[13,14].

Because H3K27me3 is globally lost in H3K27M-mutant glioma, the subsequent deregulation of gene expression is thought to lead to tumorigenesis, although the developmental timing of the mutational event is important[15]. H3K27M expression in neural stem cells has led to tumorigenesis in mice when accompanied by *TP53* knockout and/or *PDGFRA* amplification, but this combination of molecular aberrations failed to result in tumorigenesis when introduced in mature astrocytes[16,17]. However, the precise cell type of origin for H3K27M gliomas is not yet known. Candidate cell types include neuroepithelial cells (also known as neural stem cells), radial glia (also known as neural progenitor cells), and oligodendrocyte precursor cells (OPCs)[16–18].

Many important brain developmental processes are regulated by H3K27me3 deposition and could contribute to gliomagenesis if not well controlled. One of these is the epithelial-mesenchymal transition (EMT), which is essential for gastrulation, migration of neural crest cells, and neural tube formation[19–21]. The EMT is regulated by SNAI1, a transcription factor master regulator[22–24]. By regulating EMT, SNAI1 plays a critical role in many developmental processes, including gastrulation and differentiation of embryonic stem cells[25–27]. SNAI1 induces EMT through direct recruitment of PRC2, resulting in H3K27 trimethylation of key epithelial genes such as concurrently upregulating mesenchymal genes[28,29].
In the brain, processes closely resembling EMT are involved in key developmental steps such as the differentiation of neuroepithelial cells to both neuronal and glial cells[30,31]. These processes, which control cell fate and identity in early neural progenitor cell development, are regulated by EZH2[32]. Interestingly, while EMT mainly results in a differentiation event, in some

cases EMT causes increased stem cell properties[33–37]. Recent research potentially reconciles these results by introducing the hybrid epithelial/mesenchymal phenotype: the result of a partial EMT in which both epithelial and mesenchymal genes are expressed[38,39]. This process may allow cancer cells to revert to a more stem cell-like phenotype.

Given the regulation of the EMT by H3K27me3 deposition, and the disruption of this deposition by the H3K27M mutation, we sought to investigate the EMT status in pHGGs with and without the H3K27M mutation. We analyzed RNA sequencing data from 78 pHGGs obtained from three different studies. First, we performed differential expression analysis using RNA sequencing (RNA-seq) derived gene expression from bulk tumor samples, and found that H3K27M gliomas differentially express pre-EMT genes[40]. Secondly, we examined previously published cerebral organoid data and observed similarities between pre-EMT neural stem cells and H3K27M gliomas[41]. Finally, we leveraged a recent single cell RNA sequencing dataset to uncover multiple stages of EMT in H3K27M tumor cells[18]. Overall, our results suggest that the H3K27M mutation may cause an arrest in development of a neural stem cell type at an early stage of EMT, indicating a developmental window of opportunity for H3K27M occurrence.

Our study highlights the importance of genomic data sharing for rare diseases, such as pHGGs. By combining RNA sequencing data from multiple previously published studies, we were able to assemble a cohort of 78 pHGG, large enough for the differential expression analysis of pHGGs with and without the H3K27M mutation. We used this new cohort of previously published data to derive a novel biological model to describe the molecular pathogenesis of the disease.

Data Description

The RNA sequencing data from bulk clinical pediatric glioma samples used in these analyses were downloaded from the Treehouse Cancer Compendium, where it is publicly

available (treehousegenomics.soe.ucsc.edu/public-data/). All samples passed the RNA sequencing quality control analysis used in curation of the Treehouse Cancer Compendium[40]. The single cell glioma RNA sequencing data were downloaded from the Gene Expression Omnibus (accession: GSE102130), where it is publicly available. The dataset was log-normalized and filtered for low expression and low variability genes. The RNA sequencing data from glioma cell lines were accessed with permission from dbGap phs000900.v1.p1, where it is available to other researchers with permission, and all samples passed the RNA sequencing quality control analysis used in curation of the Treehouse Cancer Compendium[40]. The bulk and single cell organoid RNA sequencing data were downloaded from the Gene Expression Omnibus (accession: GSE106245), where it is publicly available. The datasets were log-normalized and filtered for low expression and low variability genes.

Analyses

A. Differential expression analysis of pediatric gliomas with and without H3K27M mutation reveals deregulation of genes involved in epithelial-mesenchymal transition.

We obtained RNA-seq data from 33 H3K27M pediatric high grade gliomas (pHGG) and 45 nonK27M pHGG from the Treehouse Childhood Cancer Initiative public cancer compendium [42] (Supplementary Table 1). These data came from several cohorts including the Pacific Pediatric Neuro-Oncology Consortium (PNOC), Dr. Michelle Monje’s studies, and The Cancer Genome Atlas[43–48].

Using the *limma* package in R[49], we conducted differential expression analysis between the H3K27M and nonK27M pHGG cohorts. A total of 1905 genes are differentially expressed between the two tumor types (Supplementary Table 2). Using Gene Set Enrichment

Analysis (GSEA) and the Molecular Signatures Database (MSigDB)[50], we found 23 biological signaling pathways with significant enrichment in coding genes overexpressed in the H3K27M cohort (Supplementary Table 2). The top 5 most significantly enriched gene pathways included “Hallmark KRAS Signaling Down” (genes repressed by KRAS activation) and the “Hallmark Epithelial Mesenchymal Transition” (Figure 1A). KRAS pathway enrichment is consistent with a recent study which found RAS signaling to be activated in H3K27M gliomas[51].

Because the epithelial-mesenchymal transition (EMT) is regulated by deposition of H3K27me3, an epigenetic transcriptional repressive mark that is lost in H3K27M cells, we were particularly interested in the differential expression of genes involved in the EMT pathway. The Hallmark EMT pathway gene list is limited to 200 genes[52], so to comprehensively characterize differential EMT activity in H3K27M mutant versus nonK27M tumors, we generated a master list of non-redundant EMT-related genes (n=1226) by merging all MSigDB EMT-related gene sets and by identifying EMT-related genes through manual literature curation (Supplementary Table 2). This list includes genes implicated in both epithelial and mesenchymal cell states, as well as several intermediate EMT cell states and EMT-like processes.

To investigate differential EMT gene expression, we calculated the overlap between the EMT master list and the differentially expressed genes (Supplementary Table 2). We found 123 differentially expressed genes from the EMT master list, indicating potential differential activity of the EMT pathway in H3K27M mutant gliomas (pvalue<2.38^{-28}, hypergeometric test). Of these genes, 73 were more highly expressed in H3K27M tumors, and the remaining 50 were more highly expressed in nonK27M tumors. (Figure 1B). Further investigation revealed that, in general, the EMT-related genes overexpressed in the H3K27M cohort are associated with epithelial-like cell states, and are normally upregulated prior to the EMT. In contrast, many of the EMT genes underexpressed in H3K27M tumors are mesenchymal markers or associated with a post-EMT cell state.

A few examples illustrate this striking trend. *SFRP1* and *SFRP2*, which are more highly expressed in H3K27M tumors, have been shown to inhibit pro-EMT transcription factors and thereby increase expression of E-cadherin in epithelial cells (*SRFP1* log fold change (LFC)=0.5, *SFRP2* LFC=0.8)[53]. *GALNT3*, which has been characterized as one of the best expression markers for epithelial cells, has higher expression in H3K27M tumors (LFC=0.6)[54]. In contrast, *GSC*/Goosecoid is a key marker of mesenchymal cells, and displays lower expression in H3K27M tumors compared to nonK27M tumors (LFC=-3.1)[55,56].

In particular, we noted that *SNAI1*, a transcription factor and key regulator of the EMT, is significantly overexpressed in H3K27M tumors (LFC=0.6; Figure 1C). High expression of *SNAI1* is a marker of EMT induction in epithelial cells. If the EMT is successful, this is followed by high expression of mesenchymal markers *TWIST1[57]*, fibronectin (*FN1*)[58], N-cadherin (*CDH2*)[59] and cadherin-11 (*CDH11*)[60]. Using a Mann-Whitney nonparametric significance test, we found significantly reduced expression of all of these mesenchymal markers in H3K27M tumors (*TWIST1* LFC=-1.2, *FN1* LFC=-0.2, *CDH2* LFC=-0.2, *CDH11* LFC=-0.3; Figure 1C). *TWIST1*, *CDH2* and *CDH11* are also underexpressed in the H3K27M cohort by the *limma* analysis.

Because *SNAI1* induces EMT by directly recruiting PRC2 methyltransferase activity for H3K27-trimethylation, a process blocked by the H3K27M mutation, we hypothesized that the occurrence of the H3K27M mutation may promote tumorigenesis by stalling EMT during early neuroepithelial differentiation. To further investigate this hypothesis, we performed comparative RNA-sequencing expression outlier analysis developed by the Treehouse Childhood Cancer Initiative, which identifies genes with outlier expression in individual samples as compared to a background cohort of highly correlated and disease-matched samples (pan-disease analysis, see Methods) [40]. We identified genes with outlier expression only in nonK27M pHGG samples (but not H3K27M pHGG samples) as compared to a background glioma cohort, and noted that many mesenchymal and post-EMT pathways were identified as enriched among the outlier genes (Supplementary Figure 1, Supplemental Table 1).

Overall, our multiple analyses of the pHGG RNA-seq cohort suggest that H3K27M pHGG tumors are associated with pre-EMT gene expression, while nonK27M pHGG tumors are characterized by post-EMT and mesenchymal gene expression.

Figure 1. The EMT pathway is differentially expressed in H3K27M gliomas as compared to nonK27M gliomas. A) Differential expression analysis of a cohort of H3K27M and nonK27M pHGG revealed significant enrichment of Hallmark Epithelial Mesenchymal Transition in genes overexpressed in H3K27M gliomas. B) Heatmap of differentially expressed EMT genes between H3K27M and nonK27M pHGG. C) SNAI1, master regulator of EMT, is overexpressed in H3K27M glioma, while mesenchymal markers TWIST1, FN1, CDH2 and CDH11 are underexpressed in H3K27M glioma as compared to nonK27M gliomas (Mann-Whitney significance test; * pvalue < 0.05, ** pvalue < 0.01, *** pvalue < 0.001).

B. H3K27M-mediated gliomagenesis is associated with pre-EMT cell types.

Consistent with our differential expression analysis, a review of the literature revealed that H3K27M-associated gliomagenesis has been experimentally recapitulated only in cell types which are poised to undergo an EMT differentiation event (Figure 2A). For example, a combination of H3K27M, *p53* loss, and *PDGFRA* constitutive activation in human neural progenitor cells (NPCs) induced low grade gliomas when injected into the pons of neonatal mice[16]. These gliomas expressed markers of pre-EMT neuroepithelial cells. Another study found that H3K27M and *Trp53* loss was sufficient for gliomagenesis in the NPCs of embryonic mice in the forebrain and hindbrain[17]. Strikingly, when introduced post-natally, H3K27M and *p53* loss in NPCs was not sufficient for gliomagenesis, although post-natal induction of H3K27M, *Trp53* loss and *PDGFRA* amplification in neural stem cells resulted in glioma formation[61,62]. Additionally, no tumorigenesis was observed upon introduction of H3K27M, *p53* loss and *PDGFRA* constitutive activation in mature astrocytes, a post-EMT cell type[16].

These observations indicate that experimental H3K27M-mediated gliomagenesis occurs in a pre-EMT cell type.

Based on our gene expression analysis and review of the literature, we hypothesized that H3K27M gliomas arise in pre-EMT cell types and retain the EMT signature of the cell type in which the mutation arises. Given this hypothesis, we expect that H3K27M gliomas harbor gene expression signatures of normal pre-EMT cell types that exist during neuronal development. In order to compare the expression of the EMT-related genes of interest between H3K27M tumors and normal developing brain cells, we examined total and single cell RNA-seq data from a human embryonic stem cell-derived cerebral cortex organoid time course experiment (Figure 2B)[41]. These organoid cultures mimic the early weeks of human prenatal cortical development and generate relevant cell types, uniquely allowing us to investigate early time-points in development which are not available in existing human fetal brain datasets. After induction of neural epithelium by week 1, at week 2 radial glia cells and Cajal-Retzius neurons are present in addition to some remaining neuroepithelial cells. By week 5, the organoids contain populations of radial glia, intermediate progenitors and deep-layer neurons.

When we investigated EMT-related gene expression in cerebral organoids during gestational weeks 1-6, we noted the presence of 2 distinct EMT processes (Figure 2A, lower panel). The first process starts as *SNAI1* expression peaks in neural stem cells (week 1), coincident with low expression of mesenchymal markers *TWIST1*, *CDH2, CDH11* and *FN1*. As differentiation from neural epithelial cells to early radial glia occurs, *SNAI1* expression decreases while mesenchymal marker expression increases. In the second process, as radial glia cells prepare to undergo a second EMT into intermediate progenitor cells, *SNAI1* expression increases once again.

To further characterize the EMT states represented in cerebral organoids, we utilized single cell RNA-seq data from the cerebral organoids at gestational weeks 3 and 6[41]. These sample collection times effectively covered all relevant cell type diversity, as gestation week 3

organoids contain substantial populations of neural epithelial cells, early radial glia cells and Cajal-Retzius neurons, while week 6 organoids are composed of late radial glia cells, intermediate progenitors, and immature neurons. We scored the EMT status of each cell using a gene signature representing EMT completion (Figure 2C, Supplementary Table 3)[18,63–66]. Neural epithelial and early (presumably pre-EMT) radial glia cells show significantly lower EMT scores than post-EMT intermediate progenitors, late radial glia and neurons (Mann-Whitney test, pvalue<0.0001). This shows that our assay contains distinct populations of pre- and post-EMT cerebral cells, and is consistent with the levels of *SNAI1*, *CDH2*, *CDH11, FN1* and *TWIST1* in the bulk weeks 1-6 organoid data. This dataset enables us to investigate transcriptional similarities between H3K27M-mutant gliomas and normal pre-EMT cell types during neural development.

We then examined the expression of genes overexpressed in H3K27M gliomas in the single cell organoid RNA-seq dataset, to see which normal cell type is most similar to H3K27M glioma cells. Of the 1180 H3K27M-overexpressed genes, 152 genes passed the single cell RNA-seq expression filter (Supplementary Table 3, see Methods). Hierarchical clustering of the expression profiles of these genes in normal cell types during neural development revealed highest expression in pre-EMT neural epithelium and early radial glia (Figure 2D). We then ranked this gene signature based on each gene’s expression in each cell type (see Methods). We found that this signature is ranked most highly in pre-EMT neural epithelium and in early radial glia (pvalue<0.05, Figure 2E).

Overall, these results suggest that the differential EMT gene expression observed in our tumor cohort is related to stages of EMT in the normal developing brain, and that H3K27M tumor cells resemble pre-EMT neural cell types.

Figure 2. H3K27M-specific EMT transcriptional signature is similar to pre-EMT neural stem cell expression in cerebral organoids. A) In vitro and in vivo experimental H3K27M-

associated gliomagenesis occurs exclusively in pre-EMT cell types (upper panel). These cell types are represented in our cerebral organoid assay, and a time course of these organoid cultures represents 2 EMT events in early brain development (lower panel). B) Experimental workflow for total RNA-seq and single cell RNA-seq from a human embryonic stem cell derived cerebral cortex organoid time course experiment. C) Single cells from cerebral organoids were scored for EMT completion. Pre-EMT neural epithelium and early radial glia were least enriched for the EMT score, while post-EMT intermediate progenitors, late radial glia and neurons were the most enriched. D) A signature of genes differentially expressed in H3K27M gliomas and expressed in cerebral organoids shows highest expression in pre-EMT neural epithelium and early radial glia. E) EMT-related genes highly expressed in H3K27M-mutant gliomas are also highly expressed in neural epithelium and early radial glia. (Mann-Whitney significance test; * pvalue < 0.05, ** pvalue < 0.01, **** pvalue < 0.0001)

C. Single-cell profiling of H3K27M gliomas reveals groups of cells at different stages of EMT.

We utilized recently published single cell RNA-seq data from 6 H3K27M and 2 H3 wild type (H3WT) gliomas to directly investigate the EMT signatures of single cell populations within each tumor type[18]. One of the H3K27M tumors harbors the mutation in the *HIST1H3B* gene (referenced as H3.1K27M), while the remaining 5 H3K27M tumors harbor the mutation in the *H3F3A* gene (referenced as H3.3K27M).

We performed hierarchical clustering of 3057 tumor cells using 629 genes from the EMT master list which passed expression filters (see Methods, Supplementary Table 4)[67]. Ten EMT-related clusters were discovered and named A-J (Figure 3A, Supplementary Table 4). Cluster gene signatures were identified by assigning each cluster the genes with maximum mean expression in that cell cluster across the dataset (Supplementary Table 4).

We assigned cluster function based on manual review of genes in each signature, and observed several populations of cells whose presence in this dataset has already been noted[18]. Cluster C has highest expression of cell cycle markers including *E2F2* and *MCM2-7*, indicating that these are actively cycling cells[68]. Cluster E is composed predominantly of non-malignant immune cells, indicated by comparatively highest expression of immune markers such as *CD68[69]*. Cluster I resembles oligodendrocytic cells, with highest expression of *CD9* and *ZEB2*, and cluster J resembles oligodendrocyte precursor cells with the highest expression of *PDGFRA[70–73]*. The presence of each of these cell types has already been noted in H3K27M gliomas, and these cell type signatures are not informative for assessing EMT state[18].

However, the remaining clusters are defined by gene expression representing various stages of EMT. We again scored the EMT status of each cell with a gene signature representing EMT completion (Figure 3A, see Methods)[18,63–66]. Cluster A scored the lowest overall, while clusters F, G, and H scored the highest overall. Cluster relationships are shown with Uniform Manifold Approximation and Projection (UMAP) in Figure 3B, and expression patterns of selected EMT marker genes are shown in the lower panel of Figure 3B. Of the EMT marker genes identified in the bulk RNA sequencing analysis (Figure 1C), only *FN1*, *CDH2* and *CDH11* were expressed in the glioma single cell RNA-seq data, so we also visualized *VIM* as a post-EMT marker and *OCLN* as a pre-EMT marker.

In keeping with our previous analysis, we noted that clusters F and G, which are composed mainly of H3WT glioma cells, strongly resemble post-EMT cells and most highly express canonical mesenchymal markers including *CDH2*, *CDH11, FN1* and *VIM[74,75]*. This is consistent with our observation that nonK27M gliomas transcriptionally resemble a post-EMT state as compared to H3K27M in the bulk RNA-seq pHGG cohort. Thus, we defined Clusters F and G “post-EMT”.

Interestingly, within the clusters composed predominantly of H3K27M cells, multiple stages of EMT emerged. Cluster A cells exhibit comparatively highest expression of several genes known to be active in epithelial or pre-EMT cell types, including *CADM1*, *EGR1, PTEN, NOTCH1,* and *OCLN[76–80]*. Additionally, cluster A cells are characterized by high expression of genes activated at the early stages of the *SMAD3*-induced EMT pathway, including *SMAD3*, *CTNNB1*, *FOS*, and *FOSB[79,81,82]*. Therefore, we defined Cluster A "pre-EMT". In contrast, Cluster B has comparatively highest expression of only 6 genes (*ACTG1, BMP2, COPA, PLXNA2, RPS27A and TP53INP1*) and has no clear expression signature of any stage of EMT, so we defined Cluster B "EMT-ambiguous".

Clusters D and H were defined "EMT-intermediate", because both clusters display high expression of genes normally expressed while the EMT process is taking place, without a clear bias towards epithelial or mesenchymal gene expression. For example, cluster D has the highest expression of *MMP2*, *VCAN*, and *SMAD2*, which are activated during the EMT process rather than before or after[79,83]. Cluster H cells display both pro-EMT and anti-EMT signaling, as evidenced by expression of genes involved in activating EMT (*TNC*, *MMP14*, and *FGFR3*), and genes implicated in suppressing EMT (*DLG5*, *LRIG1*, and *WWC1*)[84–89]. Cluster H also has the highest expression of several genes previously identified as characterizing an intermediate epithelial/mesenchymal (E/M) state (*COL6A1*, *NR2F1*, *TFPI*, *WNT5A*)[39].

Figure 3. Single cell RNA sequencing of H3K27M and nonK27M gliomas reveals multiple EMT stages within tumors. A) Expression heatmap showing hierarchical clustering of 3,057 cells from 6 H3K27M and 2 nonK27M high-grade gliomas, with a master list of EMT genes. Ten clusters (A-J) were assigned gene signatures based on maximum mean gene expression in each cluster, and clusters were classified based on manual review of each gene signature. Histone H3 mutation status and EMT score are shown at the bottom of the heatmap (ODC=oligodendrocyte, OPC=oligodendrocyte precursor). B) UMAP dimensionality reduction

projection of the same expression data as the heatmap and labeled by cluster, Histone H3 mutation status and EMT score. Expression of selected epithelial and mesenchymal genes shown in bottom panel.

D. Histone H3.1K27M glioma cells may represent a more advanced stage of EMT than H3.3K27M glioma cells.

Further examination revealed that cluster D mainly consists of cells from the H3.1K27M mutant tumor, and cluster H consists of a mixture of H3.1 and H3.3K27M cells. H3.1 and H3.3K27M characterize two functionally different subtypes of H3K27M gliomas; H3.1K27M gliomas are comparatively rare but have a slightly better prognosis[46,90]. The H3.1 histone is diffusely distributed throughout the genome, while the H3.3 histone is preferentially located at active chromatin[91–93]. This leads to distinct patterns of epigenetic reprogramming in each histone variant, where loss of the H3.3K27me3 mark is directly correlated with areas of H3.3 genomic enrichment, but H3.1K27me3 loss is not localized[93]. Because the H3K27M mutation is known to induce dose-dependent inhibition of PRC2 methyltransferase, this suggests that the localized distribution of H3.3 histone may result in higher local inhibition of PRC2 and loss of H3K27me3 at H3.3K27M sites, whereas the widespread distribution of H3.1K27M results in diffuse PRC2 inhibition[61,93]. Because precise control of gene transcription via active chromatin is necessary for a successful EMT, a H3.3K27M mutation would be particularly damaging to proper regulation of the EMT pathway. Indeed, functional analysis of enhancer regions in H3.3K27M-expressing NPCs revealed enrichment of regions positively regulating EMT, indicating that H3.3 active chromatin regions are directly involved in transcriptional control of EMT genes[93]. This suggests that EMT-poised H3.3K27M cells will be unable to properly complete EMT due to lack of transcriptional control.

Accordingly, we observed EMT-intermediate or E/M hybrid expression genes in glioma single-cell clusters D and H, both of which have substantial numbers of H3.1K27M glioma cells. We hypothesized that H3.1K27M cells may be more differentiated and farther along the EMT process than H3.3K27M cells.

In order to investigate this hypothesis further, we subset the single cell glioma RNA-seq data to 2458 cells with H3.1K27M or H3.3K27M mutation and performed Wilcoxon rank-sum test to identify genes overexpressed in each variant group (Supplementary Table 4; Supplementary Figure 2). Consistent with our previous observations, GSEA of Gene Ontology (GO) gene sets (Figure 4B, Supplementary Table 4) revealed enrichment of epithelial gene sets in H3.3K27M compared to H3.1K27M (GO Adhesion pathways, GO Neurogenesis, GO Embryo Development) and mesenchymal gene sets in H3.1K27M compared to H3.3K27M (GO EMT pathway, GO Mesenchymal Cell Differentiation and GO Mesenchyme Development). Additionally, scoring of all cells for EMT completeness shows that H3.1K27M cells score significantly higher overall than H3.3K27M cells, while nonK27M cells score significantly higher than either mutant cell type (Supplementary Figure 3). However, because the H3.1K27M cells come from a single tumor, we performed additional analysis to investigate this observation. We cultured diffuse intrinsic pontine glioma (DIPG) primary cell lines isolated in a previous study to investigate the expression of EMT markers in H3.3K27M, H3.1K27M and nonK27M glioma cells[94]. Morphologically, we observed that when cultured in serum-free conditions, the H3.1K27M cell lines preferentially grow attached to the flask (4 of 5 cell lines), while the H3.3K27M cells preferentially grow as neurospheres (8 of 9 cell lines) (Figure 4C). Because differentiation of neurospheres is accompanied by attachment and increased expression of N-cadherin, this morphological trend is consistent with our hypothesis that H3.1K27M cells exist in a more differentiated state than H3.3K27M cells[95].

We analyzed RNA-seq data from 3 DIPG cell lines to compare the expression of EMT genes (SU-DIPG-IV is H3.1K27M mutant; SU-DIPG-VI and JHH-DIPG1 are H3.3K27M mutant).

We used 4 replicate samples from each SU-DIPG-IV and SU-DIPG-VI and 3 replicate samples from JHH-DIPG1. Each sample was scored using a gene signature of EMT completion (see Methods), and the H3.1K27M samples scored significantly higher than the H3.3K27M samples (Figure 4D, pvalue<0.05).

We then performed RT-PCR to quantify expression of *FN1* and *CDH2*, canonical mesenchymal marker genes which were previously identified in the bulk glioma RNA sequencing analysis (Figure 4E, full-length gel in Supplementary Figure 4). We attempted to quantify E-cadherin/*CDH1* as it is a canonical epithelial marker, but the levels were so low as to be undetectable by RT-PCR in these cell lines (RNA-seq <1.0 log2(TPM+1)). We compared 9 H3.3K27M cell lines (SU-DIPG-VI, XIII, XVII, XIX, 24, 25, 27, 35 and 43) with 5 H3.1K27M cell lines (SU-DIPG-IV, XXI, 33, 36 and 38) and included 5 H3 wild-type lines (SU-DIPG-48, pcGBM2R, KNS42, SJG2 and normal human astrocytes hTERT) and a negative RT-PCR control (NC). Overall, the H3 wild-type and H3.1K27M cell lines appear to more highly express both mesenchymal markers, in keeping with the bulk and single-cell RNA-seq analyses. Our computational and *in vitro* observations are consistent with a recent study indicating that H3.1K27M tumor cells are overall more differentiated than H3.3K27M tumor cells[93]. Overall, these data suggest that the histone H3K27M mutation is associated with a preferentially early or pre-EMT cell state as compared to nonK27M cells, but that H3.1K27M cells may represent a somewhat later or intermediate-EMT cell state as compared to H3.3K27M cells.

Figure 4. H3.1K27M glioma cells appear more mesenchymal than H3.3K27M glioma cells. A) UMAP dimensionality reduction of 2458 histone mutant glioma single cells. B) Gene set enrichment analysis of genes overexpressed in H3.3K27M versus H3.1K27M (top graph) or H3.1K27M versus H3.3K27M (lower graph) by Wilcoxon rank-sum test using glioma single cell RNA-seq data. C) Representative images of H3.1K27M and H3.3K27M glioma derived cell cultures. Scale bar 400 um. D) Total RNA sequencing datasets from glioma cell lines were

scored for EMT completeness (4 samples from SU-DIPG-IV, 4 samples from SU-DIPG-VI and 3 samples from JHH-DIPG1). Scoring is shown in a heatmap and a boxplot. (Mann-Whitney significance test; * pvalue < 0.05) E) RT-PCR of FN1 and CDH2 expression in glioma primary cell cultures (all numbered lines are SU-DIPG).

Discussion

H3K27M diffuse midline gliomas are aggressive tumors generally occurring in early childhood in the hindbrain or midline. These tumors have poor prognosis and do not respond to standard chemotherapies for adult gliomas[96]. Unlike most adult cancers, pediatric cancers, including pediatric gliomas, are thought to have a developmental origin[15,46,97]. The temporal- and region-specific occurrence of pediatric diffuse midline gliomas reinforces this possible developmental origin. EZH2-deposited H3K27me3 transcriptional marks are known to have crucial roles in cell differentiation and development in the brain and are lost in H3K27M cells[6]. Research has implicated the epithelial mesenchymal transition in pediatric gliomas[98,99], particularly those with a more invasive phenotype. A large portion of diffuse midline glioma tumors highly express genes known to be involved in EMT occurring in adult glioblastomas[100]. EZH2 appears to play an important role in EMT in adult gliomas: EZH2 depletion in adult glioblastomas leads to a reduction in expression of mesenchymal markers, and an increase in epithelial markers[6]. Other studies suggest EZH2 is important for the invasion of gliomas[101–103]. Thus, a molecular aberration affecting the activity of EZH2 might prevent a complete epithelial-mesenchymal transition.

In this study, we observed that various canonical EMT-inducing genes are significantly overexpressed in H3K27M mutant pHGGs, compared to nonK27M pHGGs, while many canonical mesenchymal markers are underexpressed in H3K27M pHGGs as compared to the nonK27M tumors. In particular, we noted higher expression of the pro-EMT transcription factor

SNAI1 in H3K27M-mutant gliomas. Because *SNAI1* relies on PRC2 and H3K27me3 to facilitate EMT through gene expression regulation, this may indicate an arrest in the EMT process. The existence of a hybrid epithelial/mesenchymal phenotype is well-established: the result of a partial EMT is the expression of both epithelial and mesenchymal genes[38]. Studies have shown that a hybrid E/M phenotype may indicate a worse prognosis than mesenchymal-only states in solid tumors[38,39,104].

We hypothesized that if H3K27M mutation prevents full EMT, neural stem cells harboring H3K27M may be forced to retain a proliferative, stem cell phenotype, eventually leading to tumorigenic development. Accordingly, we observed from extensive literature review that experimental induction of H3K27M-associated gliomas has occurred exclusively in pre-EMT cell types, and that two consecutive EMT processes occur early in normal brain development. Single cell RNA-seq from H3K27M and nonK27M tumors confirmed a more mesenchymal expression signature in the nonK27M cells, and also revealed subsets of H3K27M cells at various stages of EMT. Specifically, we observed an intermediate EMT signature in the H3.1K27M cells as compared to the more epithelial H3.3K27M cells. This was also observed in bulk RNA-seq and *in vitro* analysis. We hypothesize that because the H3.1K27M mutation is not concentrated at active chromatin, it has less repressive power as specific developmental pathways such as EMT are activated over time. If a subset of H3.1K27M cells are able to differentiate through the EMT, this may explain why H3.1K27M gliomas have a slightly better prognosis.

To conclude, we mined 3 publicly available RNA-seq datasets from pediatric gliomas and cerebral organoids to generate a hypothesis for the gliomagenesis of H3K27M gliomas. We propose that the H3K27M mutation is tumorigenic when the mutational hit occurs in a cell poised to undergo the EMT, due to the dependence of normal EMT on the correct timing of the H3K27me3 mark (Figure 5). More work is needed to characterize the observed difference in the EMT status between the H3.1 and H3.3K27M variants. These results hold important

implications for better understanding the developmental origin and timing of these aggressive and untreatable cancers. Further, the presence of an epigenetically-driven differentiation stall may imply that a pharmacological methylation agent or a pro-differentiation therapy may aid in future treatment of H3K27M mutant tumors[105].

Figure 5. Proposed model for EMT stall in H3K27M cells. We propose that H3K27M cells retain high levels of SNAI1 expression but remain stalled in a pre-EMT state due to inability of PCR2 to tri-methylate H3K27.

Potential Implications

Our study holds implications for other diseases, because H3K27M mutation is not exclusive to diffuse midline gliomas. It can also be found in a fraction of pediatric ependymomas and medulloblastomas[106]. Interestingly, ependymomas located in the posterior fossa typically do not harbor the H3K27M mutation, but exhibit the K27M-associated H3K27 hypomethylation phenotype. Thus, the proposed EMT arrest and differentiation stall as a result of H3K27me3 loss may also apply to these cancers. Beyond the SNAI1-H3K27me3 axis, EMT is also regulated by other epigenetic marks[107]. Given the epigenetically dysfunctional nature of many pediatric cancers[15], EMT arrest could conceivably play a role in the oncogenesis of these tumors as well.

Methods

Glioma bulk RNA sequencing data

Gene expression data from 78 pediatric high grade glioma samples were downloaded from the Treehouse Childhood Cancer Initiative public compendium v8[42]. All samples in the compendium have been uniformly processed using the UC Santa Cruz TOIL RNA-seq pipeline

(v3.3.4)[108]. This dataset (n=58581 genes) is in transcripts per million (TPM) and normalized by log2(TPM+1). We divided the dataset into 33 H3K27M mutant samples and 45 nonK27M samples, and performed differential expression analysis of all genes between the two groups using R library *limma* v3.34.9 in R v3.3.4. We performed gene set enrichment analysis (GSEA) of the resulting 1905 differentially expressed genes (pvalue<0.1) with Molecular Signatures Database (MSigDB) v7.0 on the GSEA/MSigDB web site v6.4 (Supplementary Table 2). Since the epithelial-mesenchymal transition (EMT) pathway was in the top 5 most significantly enriched pathways in H3K27M over expressed genes, we created a non-redundant master list of EMT genes (n=1226) by merging 15 EMT related MSigDB pathways and by identifying EMT-related genes through manual literature curation (Supplementary Table 2).

We performed pan-disease outlier analysis on all the pHGG samples using Treehouse CARE (see Availability of source code and requirements section) against the Treehouse Cancer Compendium v10. Pan-disease outlier analysis identifies genes with outlier expression in each sample of interest as compared to a background cohort of tumors identified as most similar[40]. We identified a list of genes with outlier expression in the nonK27M pHGG samples that did not also have outlier expression in the H3K27M pHGG samples, and performed gene set enrichment analysis using Enrichr in the GSEApy package (gseapy-v0.9.17)[109] against BioPlanet_2019 library with p-value cutoff 0.05 (outlier genes and enriched pathways in Supplementary Table 2). We used the EnrichmentMap app in Cytoscape to visualize functionally similar clusters of enriched pathways[110].

Cerebral organoid RNA sequencing data (bulk and single cell)

Gene expression data (TPM) from 6 weekly timepoints of human cerebral organoid growth were downloaded from accession GSE106245[41]. Organoid weeks 0-5 were converted to gestational weeks 1-6 and duplicate gene measurements were averaged. For Figure 2A, expression of each gene was normalized between 0-1. Single cell RNA sequencing data from

weeks 2 and 5 (gestational weeks 3 and 6) cerebral organoids were downloaded from accession GSE106245[41]. Expression data were filtered to remove genes with expression in fewer than 10% of cells. Cell types were assigned using a list of marker genes (Supplementary Table 3).

Glioma single cell RNA sequencing data

Smart-seq2 RSEM TPM single cell RNA sequencing data from 3,057 glioma cells were downloaded from accession GSE102130[18]. Data were log2-normalized and filtered to remove genes with expression in fewer than 20% of cells. Hierarchical clustering of all cells was performed using the Python *scipy.cluster.hierarchy* function (scipy v1.4.1) after subsetting to a non-redundant master list of EMT genes (n=1226, Supplementary Table 2). Of these genes, 629 passed the expression filter and were included in the hierarchical clustering. The clustering results were plotted using the *scipy.cluster.hierarchy.dendrogram* function with threshold set to 3.5. Gene signatures for each cluster were assigned by identifying the cluster in which each gene has maximum mean expression, and assigning that gene to that cluster. For UMAP visualizations, Leiden clustering was performed on the single cell data using the *scanpy.tl.leiden* function (scanpy v1.4.5.post1) with resolution set to 0.5 and top 10 principle components used as input.

DIPG Cell Lines

The patient-derived DIPG cell lines (SU-DIPG-IV, SU-DIPG-VI, SU-DIPG-XIII, SU-DIPG-XVII, SU-DIPG-XIX, SU-DIPG-XXI, SU-DIPG-24, SU-DIPG-25, SU-DIPG-27, SU-DIPG-33, SU-DIPG-35, SU-DIPG-36, SU-DIPG-38, SU-DIPG-48) were kindly provided by Dr. Michelle Monje (Stanford University School of Medicine, Stanford CA)[44]. SU-DIPG-IV, SU-DIPG-XXI, SU-DIPG-33, SU-DIPG-36, and SU-DIPG-38 cells harbor a H3.1K27M mutation while SU-DIPG-VI,

SU-DIPG-XIII, SU-DIPG-XVII, SU-DIPG-XIX, SU-DIPG-24, SU-DIPG-25, SU-DIPG-27, SU-DIPG-35, SU-DIPG-43 cells harbor a H3.3K27M mutation. SU-DIPG-48 and Glioblastoma cell line SU-pcGBM-2 are H3WT. Glioblastoma H3WT cell lines; KNS-42 (RRID:CVCL_0378), SJ-GBM2 (RRID:CVCL_M141), and one normal astrocyte cell line NHA hTERT were kindly provided by Prof. Sameer Agnihotri (UPMC Children's Hospital of Pittsburgh, Pittsburgh PA). The Universal Mycoplasma Detection Kit (AACC) was used for testing SU-DIPG-XIII, XVII, XIX, and VI latest on January 10, 2020. All cells were cultured in tumor stem medium containing 50X B-27 Supplement Minus Vitamin A (Invitrogen), H-EGF at 20ng/mL (Shenandoah Biotechnology), H-FGF-basic-154 at 20ng/mL (Shenandoah Biotechnology), H-PDGF-AA at 10ng/mL (Shenandoah Biotechnology), H-PDGF-BB at 10ng/mL (Shenandoah Biotechnology), and 0.2% Heparin Solution at 2ug/mL (STEMCELL Technologies). All experiments used cells collected within 5 passages after thawing. The cells were passaged by the treatment of TrypLE (Gibco) and DNAse I (Worthington) rocking at 37°C for 5-15 minutes then HBSS (Corning) was added to deactivate TrypLE. The cells were transferred to new Nunc EasYFlask Cell Culture Flasks (ThermoFisher Scientific) and grown in tumor stem medium as previously described. The bulk RNA sequencing data from lines SU-DIPG-VI, SU-DIPG-IV and JHH-DIPG1 were obtained with permission from Dr. Michelle Monje from dbGap accession phs000900.v1.p1.

RNA Extraction and RT-PCR

Total RNA was extracted from cell pellets using the Quick-RNA Miniprep Kit (Zymo Research). cDNA was synthesized from 1 ug of total RNA using Oligo(dT)20 primers and the SuperScript III First Strand Synthesis System (Invitrogen). PCR was performed using KAPA HiFi HotStart ReadyMixPCR Kit (KAPA Biosystems), 50 ng of template DNA and the appropriate primers and 27 PCR cycles. *CDH2* primer sequences: forward: ggcttaatggtgattttgctcag reverse: tccataccacaaacatcagcac. *FN1* primer sequences: forward: cttgaaccaacctacggatgac reverse: tcccatcatcataacacgttgc. Primer oligos were purchased from Integrated DNA Technologies.

Data Analysis

All statistical comparisons are performed with a two-sided Mann-Whitney test, with measurements taken from distinct samples without assumption of normality. Single cell and bulk tumor samples were scored for EMT activity using a manually curated set of mesenchymal genes and a scoring method based on aggregate expression of the gene set as compared to a control gene set (Supplementary Table 3)[18,64,111].

Availability of source code and requirements

Code for figures and data analysis: github.com/lauren-sanders/EMT-paper/

Code for outlier analysis: github.com/UCSC-Treehouse/CARE/

Operating system: Platform independent

Programming languages: Python, R

Availability of supporting data and materials

All data used in the manuscript is available at the following websites or accession numbers: Publicly available: 1) bulk glioma RNA-seq: treehousegenomics.soe.ucsc.edu/public-data, 2) cerebral organoid RNA-seq: GSE106245, 3) glioma single-cell RNAseq: GSE102130. Data available with permission for glioma cell line RNA-seq data: dbGap phs000900.v1.p1.

Declarations

List of abbreviations

DIPG: diffuse intrinsic pontine glioma; E/M: epithelial/mesenchymal; EMT: epithelial-mesenchymal transition; GO: gene ontology; GSEA: gene set enrichment analysis; H3WT: histone 3 wild-type; MSigDB: molecular signatures database; NPC: neural progenitor cells;

ODC: oligodendrocyte cells; OPC: oligodendrocyte precursor cells; pHGG: pediatric high-grade gliomas; PNOC: Pacific Pediatric Neuro-Oncology Consortium; TPM: transcripts per million; UMAP: Uniform Manifold Approximation and Projection; WHO: World Health Organization.

Ethics Statement

The protocols for the PNOC-003 trial, Dr. Michelle Monje's studies, Dr. Mariella Filbin's studies, The Cancer Genome Atlas, the Children's Brain Tumor Tissue Consortium, the International Cancer Genome Consortium, and the University of Michigan Clinical Sequencing Exploratory Research have been previously described[18,43–48]. The UCSC Treehouse Childhood Cancer Initiative protocol was approved by the UCSC Institutional Review Board (No. HS2648)[40].

Funding and Acknowledgements

This study was funded by American Association for Cancer Research NextGen Grant for Transformative Cancer Research Award (OMV), St Baldrick's Foundation Consortium Award and Emily Beazley Kures for Kids Fund Hero Award (DH), Alex's Lemonade Stand Foundation for Childhood Cancer Research, Unravel Pediatric Cancer, Team G Childhood Cancer Foundation, and Live for Others Foundation, The Schmidt Futures Foundation (DH), CIRM Shared Stem Cell Facilities (CL1-00506) award to UCSC. AC is supported by the T32GM133391 Training Program in Molecular, Cell, and Developmental Biology. DH is a Howard Hughes Medical Institute Investigator. OMV holds a Colligan Presidential Chair in Pediatric Genomics. We gratefully acknowledge Dr. Michelle Monje and Prof. Sameer Agnihotri who provided cell lines used in this study.

Author Contributions

Analysis and manuscript authorship: LMS and AC

Single cell organoid cell type gene ranking: LS
Experimental work: AC, AB, MC
Treehouse cancer compendium and manuscript review: HCB, ETK, JP, KL, AGL and IB
Scientific oversight and manuscript review: DH, SRS and OMV

Competing Interests

The authors declare no potential conflicts of interest.

References

1. Juratli TA, Qin N, Cahill DP, Filbin MG. Molecular pathogenesis and therapeutic implications in pediatric high-grade gliomas. Pharmacol Ther. 2018;182: 70–79.

2. Chan K-M, Fang D, Gan H, Hashizume R, Yu C, Schroeder M, et al. The histone H3.3K27M mutation in pediatric glioma reprograms H3K27 methylation and gene expression. Genes Dev. 2013;27: 985–990.

3. Johung TB, Monje M. Diffuse Intrinsic Pontine Glioma: New Pathophysiological Insights and Emerging Therapeutic Targets. Curr Neuropharmacol. 2017;15: 88–97.

4. Jones C, Baker SJ. Unique genetic and epigenetic mechanisms driving paediatric diffuse high-grade glioma. Nat Rev Cancer. 2014;14. doi:10.1038/nrc3811

5. Louis DN, Perry A, Reifenberger G, von Deimling A, Figarella-Branger D, Cavenee WK, et al. The 2016 World Health Organization Classification of Tumors of the Central Nervous System: a summary. Acta Neuropathol. 2016;131: 803–820.

6. de Vries NA, Hulsman D, Akhtar W, de Jong J, Miles DC, Blom M, et al. Prolonged Ezh2

Depletion in Glioblastoma Causes a Robust Switch in Cell Fate Resulting in Tumor Progression. Cell Rep. 2015;10: 383–397.

7. Mohammad F, Weissmann S, Leblanc B, Pandey DP, Højfeldt JW, Comet I, et al. EZH2 is a potential therapeutic target for H3K27M-mutant pediatric gliomas. Nat Med. 2017;23: 483–492.

8. Margueron R, Reinberg D. The Polycomb complex PRC2 and its mark in life. Nature. 2011;469: 343–349.

9. Mohn F, Weber M, Rebhan M, Roloff TC, Richter J, Stadler MB, et al. Lineage-specific polycomb targets and de novo DNA methylation define restriction and potential of neuronal progenitors. Mol Cell. 2008;30: 755–766.

10. Roidl D, Hacker C. Histone methylation during neural development. Cell Tissue Res. 2014;356: 539–552.

11. Sher F, Boddeke E, Olah M, Copray S. Dynamic changes in Ezh2 gene occupancy underlie its involvement in neural stem cell self-renewal and differentiation towards oligodendrocytes. PLoS One. 2012;7: e40399.

12. Sher F, Rössler R, Brouwer N, Balasubramaniyan V, Boddeke E, Copray S. Differentiation of neural stem cells into oligodendrocytes: involvement of the polycomb group protein Ezh2. Stem Cells. 2008;26: 2875–2883.

13. Akizu N, Martínez-Balbás MA. EZH2 orchestrates apicobasal polarity and neuroepithelial cell renewal. Neurogenesis (Austin). 2016;3: e1250034.

14. Zemke M, Draganova K, Klug A, Schöler A, Zurkirchen L, Gay MH-P, et al. Loss of Ezh2 promotes a midbrain-to-forebrain identity switch by direct gene derepression and Wnt-

dependent regulation. BMC Biol. 2015;13: 103.

15. Filbin M, Monje M. Developmental origins and emerging therapeutic opportunities for childhood cancer. Nat Med. 2019;25: 367–376.

16. Funato K, Major T, Lewis PW, Allis CD, Tabar V. Use of human embryonic stem cells to model pediatric gliomas with H3.3K27M histone mutation. Science. 2014;346: 1529–1533.

17. Pathania M, De Jay N, Maestro N, Harutyunyan AS, Nitarska J, Pahlavan P, et al. H3.3K27M Cooperates with Trp53 Loss and PDGFRA Gain in Mouse Embryonic Neural Progenitor Cells to Induce Invasive High-Grade Gliomas. Cancer Cell. 2017;32: 684–700.e9.

18. Filbin MG, Tirosh I, Hovestadt V, Shaw ML, Escalante LE, Mathewson ND, et al. Developmental and oncogenic programs in H3K27M gliomas dissected by single-cell RNA-seq. Science. 2018;360: 331–335.

19. Viebahn C. Epithelio-Mesenchymal Transformation during Formation of the Mesoderm in the Mammalian Embryo. Acta Anal. 1995. Available: https://www.karger.com/Article/PDF/147753

20. Duband J-L. Diversity in the molecular and cellular strategies of epithelium-to-mesenchyme transitions: Insights from the neural crest. Cell Adh Migr. 2010;4: 458–482.

21. Kalcheim C. Epithelial-Mesenchymal Transitions during Neural Crest and Somite Development. J Clin Med Res. 2015;5. doi:10.3390/jcm5010001

22. Bolós V, Peinado H, Pérez-Moreno MA, Fraga MF, Esteller M, Cano A. The transcription factor Slug represses E-cadherin expression and induces epithelial to mesenchymal transitions: a comparison with Snail and E47 repressors. J Cell Sci. 2003;116: 499–511.

23. Cano A, Pérez-Moreno MA, Rodrigo I, Locascio A, Blanco MJ, del Barrio MG, et al. The

transcription factor snail controls epithelial-mesenchymal transitions by repressing E-cadherin expression. Nat Cell Biol. 2000;2: 76–83.

24. Lin Y, Dong C, Zhou BP. Epigenetic regulation of EMT: the Snail story. Curr Pharm Des. 2014;20: 1698–1705.

25. Galvagni F, Lentucci C, Neri F, Dettori D, De Clemente C, Orlandini M, et al. Snai1 promotes ESC exit from the pluripotency by direct repression of self-renewal genes. Stem Cells. 2015;33: 742–750.

26. Murray SA, Gridley T. Snail family genes are required for left-right asymmetry determination, but not neural crest formation, in mice. Proc Natl Acad Sci U S A. 2006;103: 10300–10304.

27. Carver EA, Jiang R, Lan Y, Oram KF, Gridley T. The mouse snail gene encodes a key regulator of the epithelial-mesenchymal transition. Mol Cell Biol. 2001;21: 8184–8188.

28. Motta FJN, Valera ET, Lucio-Eterovic AKB, Queiroz RGP, Neder L, Scrideli CA, et al. Differential expression of E-cadherin gene in human neuroepithelial tumors. Genet Mol Res. 2008;7: 295–304.

29. Howng S-L, Wu C-H, Cheng T-S, Sy W-D, Lin P-CK, Wang C, et al. Differential expression of Wnt genes, beta-catenin and E-cadherin in human brain tumors. Cancer Lett. 2002;183: 95–101.

30. Itoh Y, Moriyama Y, Hasegawa T, Endo TA, Toyoda T, Gotoh Y. Scratch regulates neuronal migration onset via an epithelial-mesenchymal transition-like mechanism. Nat Neurosci. 2013;16: 416–425.

31. Ohayon D, Garcès A, Joly W, Soukkarieh C, Takagi T, Sabourin J-C, et al. Onset of

Spinal Cord Astrocyte Precursor Emigration from the Ventricular Zone Involves the Zeb1 Transcription Factor. Cell Rep. 2016;17: 1473–1481.

32. Hirabayashi Y, Suzki N, Tsuboi M, Endo TA, Toyoda T, Shinga J, et al. Polycomb limits the neurogenic competence of neural precursor cells to promote astrogenic fate transition. Neuron. 2009;63: 600–613.

33. Li Q, Hutchins AP, Chen Y, Li S, Shan Y, Liao B, et al. A sequential EMT-MET mechanism drives the differentiation of human embryonic stem cells towards hepatocytes. Nat Commun. 2017;8: 15166.

34. Mani SA, Guo W, Liao M-J, Eaton EN, Ayyanan A, Zhou AY, et al. The epithelial-mesenchymal transition generates cells with properties of stem cells. Cell. 2008;133: 704–715.

35. Scheel C, Weinberg RA. Cancer stem cells and epithelial-mesenchymal transition: concepts and molecular links. Semin Cancer Biol. 2012;22: 396–403.

36. Ullmann U, In't Veld P, Gilles C, Sermon K, De Rycke M, Van de Velde H, et al. Epithelial-mesenchymal transition process in human embryonic stem cells cultured in feeder-free conditions. Mol Hum Reprod. 2007;13: 21–32.

37. Wang H, Unternaehrer JJ. Epithelial-mesenchymal Transition and Cancer Stem Cells: At the Crossroads of Differentiation and Dedifferentiation. Dev Dyn. 2019;248: 10–20.

38. Christiansen JJ, Rajasekaran AK. Reassessing epithelial to mesenchymal transition as a prerequisite for carcinoma invasion and metastasis. Cancer Res. 2006;66: 8319–8326.

39. Grosse-Wilde A, Fouquier d'Hérouël A, McIntosh E, Ertaylan G, Skupin A, Kuestner RE, et al. Stemness of the hybrid Epithelial/Mesenchymal State in Breast Cancer and Its Association with Poor Survival. PLoS One. 2015;10: e0126522.

40. Vaske OM, Bjork I, Salama SR, Beale H, Tayi Shah A, Sanders L, et al. Comparative Tumor RNA Sequencing Analysis for Difficult-to-Treat Pediatric and Young Adult Patients With Cancer. JAMA Netw Open. 2019;2: e1913968.

41. Field AR, Jacobs FMJ, Fiddes IT, Phillips APR, Reyes-Ortiz AM, LaMontagne E, et al. Structurally Conserved Primate LncRNAs Are Transiently Expressed during Human Cortical Differentiation and Influence Cell-Type-Specific Genes. Stem Cell Reports. 2019;12: 245–257.

42. Treehouse Public Data. [cited 21 Apr 2020]. Available: https://treehousegenomics.soe.ucsc.edu/public-data/

43. Mueller S, Jain P, Liang WS, Kilburn L, Kline C, Gupta N, et al. A pilot precision medicine trial for children with diffuse intrinsic pontine glioma - PNOC003: a report from the Pacific Pediatric Neuro-Oncology Consortium. Int J Cancer. 2019. doi:10.1002/ijc.32258

44. Grasso CS, Tang Y, Truffaux N, Berlow NE, Liu L, Debily M-A, et al. Functionally defined therapeutic targets in diffuse intrinsic pontine glioma. Nat Med. 2015;21: 555–559.

45. Ceccarelli M, Barthel FP, Malta TM, Sabedot TS, Salama SR, Murray BA, et al. Molecular Profiling Reveals Biologically Discrete Subsets and Pathways of Progression in Diffuse Glioma. Cell. 2016;164: 550–563.

46. Mackay A, Burford A, Carvalho D, Izquierdo E, Fazal-Salom J, Taylor KR, et al. Integrated Molecular Meta-Analysis of 1,000 Pediatric High-Grade and Diffuse Intrinsic Pontine Glioma. Cancer Cell. 2017;32: 520–537.e5.

47. Robinson DR, Wu Y-M, Lonigro RJ, Vats P, Cobain E, Everett J, et al. Integrative clinical genomics of metastatic cancer. Nature. 2017;548: 297–303.

48. Sturm D, Orr BA, Toprak UH, Hovestadt V, Jones DTW, Capper D, et al. New Brain

Tumor Entities Emerge from Molecular Classification of CNS-PNETs. Cell. 2016;164: 1060–1072.

49. Ritchie ME, Phipson B, Wu D, Hu Y, Law CW, Shi W, et al. limma powers differential expression analyses for RNA-sequencing and microarray studies. Nucleic Acids Res. 2015;43: e47.

50. Subramanian A, Tamayo P, Mootha VK, Mukherjee S, Ebert BL, Gillette MA, et al. Gene set enrichment analysis: a knowledge-based approach for interpreting genome-wide expression profiles. Proc Natl Acad Sci U S A. 2005;102: 15545–15550.

51. Koncar RF, Dey BR, Stanton A-CJ, Agrawal N, Wassell ML, McCarl LH, et al. Identification of Novel RAS Signaling Therapeutic Vulnerabilities in Diffuse Intrinsic Pontine Gliomas. Cancer Res. 2019;79: 4026–4041.

52. Liberzon A, Birger C, Thorvaldsdóttir H, Ghandi M, Mesirov JP, Tamayo P. The Molecular Signatures Database (MSigDB) hallmark gene set collection. Cell Syst. 2015;1: 417–425.

53. Chung M-T, Lai H-C, Sytwu H-K, Yan M-D, Shih Y-L, Chang C-C, et al. SFRP1 and SFRP2 suppress the transformation and invasion abilities of cervical cancer cells through Wnt signal pathway. Gynecol Oncol. 2009;112: 646–653.

54. Maupin KA, Sinha A, Eugster E, Miller J, Ross J, Paulino V, et al. Glycogene expression alterations associated with pancreatic cancer epithelial-mesenchymal transition in complementary model systems. PLoS One. 2010;5: e13002.

55. Taube JH, Herschkowitz JI, Komurov K, Zhou AY, Gupta S, Yang J, et al. Core epithelial-to-mesenchymal transition interactome gene-expression signature is associated with claudin-low and metaplastic breast cancer subtypes. Proc Natl Acad Sci U S A. 2010;107:

15449–15454.

56. Xue T-C, Ge N-L, Zhang L, Cui J-F, Chen R-X, You Y, et al. Goosecoid promotes the metastasis of hepatocellular carcinoma by modulating the epithelial-mesenchymal transition. PLoS One. 2014;9: e109695.

57. Tran DD, Corsa CAS, Biswas H, Aft RL, Longmore GD. Temporal and spatial cooperation of Snail1 and Twist1 during epithelial-mesenchymal transition predicts for human breast cancer recurrence. Mol Cancer Res. 2011;9: 1644–1657.

58. Stanisavljevic J, Porta-de-la-Riva M, Batlle R, de Herreros AG, Baulida J. The p65 subunit of NF-κB and PARP1 assist Snail1 in activating fibronectin transcription. J Cell Sci. 2011;124: 4161–4171.

59. Javaid S, Zhang J, Anderssen E, Black JC, Wittner BS, Tajima K, et al. Dynamic chromatin modification sustains epithelial-mesenchymal transition following inducible expression of Snail-1. Cell Rep. 2013;5: 1679–1689.

60. Tanaka S, Kobayashi W, Haraguchi M, Ishihata K, Nakamura N, Ozawa M. Snail1 expression in human colon cancer DLD-1 cells confers invasive properties without N-cadherin expression. Biochem Biophys Rep. 2016;8: 120–126.

61. Lewis PW, Müller MM, Koletsky MS, Cordero F, Lin S, Banaszynski LA, et al. Inhibition of PRC2 activity by a gain-of-function H3 mutation found in pediatric glioblastoma. Science. 2013;340: 857–861.

62. Larson JD, Kasper LH, Paugh BS, Jin H, Wu G, Kwon C-H, et al. Histone H3.3 K27M Accelerates Spontaneous Brainstem Glioma and Drives Restricted Changes in Bivalent Gene Expression. Cancer Cell. 2019;35: 140–155.e7.

63. Tirosh I, Venteicher AS, Hebert C, Escalante LE, Patel AP, Yizhak K, et al. Single-cell RNA-seq supports a developmental hierarchy in human oligodendroglioma. Nature. 2016;539: 309–313.

64. Neftel C, Laffy J, Filbin MG, Hara T, Shore ME, Rahme GJ, et al. An Integrative Model of Cellular States, Plasticity, and Genetics for Glioblastoma. Cell. 2019. doi:10.1016/j.cell.2019.06.024

65. Tan TZ, Miow QH, Miki Y, Noda T, Mori S, Huang RY-J, et al. Epithelial-mesenchymal transition spectrum quantification and its efficacy in deciphering survival and drug responses of cancer patients. EMBO Mol Med. 2014;6: 1279–1293.

66. Mak MP, Tong P, Diao L, Cardnell RJ, Gibbons DL, William WN, et al. A Patient-Derived, Pan-Cancer EMT Signature Identifies Global Molecular Alterations and Immune Target Enrichment Following Epithelial-to-Mesenchymal Transition. Clin Cancer Res. 2016;22: 609–620.

67. Virtanen P, Gommers R, Oliphant TE, Haberland M, Reddy T, Cournapeau D, et al. SciPy 1.0--Fundamental Algorithms for Scientific Computing in Python. arXiv [cs.MS]. 2019. Available: http://arxiv.org/abs/1907.10121

68. Lukas J, Petersen BO, Holm K, Bartek J, Helin K. Deregulated expression of E2F family members induces S-phase entry and overcomes p16INK4A-mediated growth suppression. Mol Cell Biol. 1996;16: 1047–1057.

69. Holness CL, Simmons DL. Molecular cloning of CD68, a human macrophage marker related to lysosomal glycoproteins. Blood. 1993;81: 1607–1613.

70. Nakamura Y, Iwamoto R, Mekada E. Expression and distribution of CD9 in myelin of the central and peripheral nervous systems. Am J Pathol. 1996;149: 575–583.

71. Weng Q, Chen Y, Wang H, Xu X, Yang B, He Q, et al. Dual-mode modulation of Smad signaling by Smad-interacting protein Sip1 is required for myelination in the central nervous system. Neuron. 2012;73: 713–728.

72. Kagawa T, Mekada E, Shishido Y, Ikenaka K. Immune system-related CD9 is expressed in mouse central nervous system myelin at a very late stage of myelination. J Neurosci Res. 1997;50: 312–320.

73. Richardson WD, Pringle N, Mosley MJ, Westermark B, Dubois-Dalcq M. A role for platelet-derived growth factor in normal gliogenesis in the central nervous system. Cell. 1988;53: 309–319.

74. Zeisberg M, Neilson EG. Biomarkers for epithelial-mesenchymal transitions. J Clin Invest. 2009;119: 1429–1437.

75. Sancisi V, Gandolfi G, Ragazzi M, Nicoli D, Tamagnini I, Piana S, et al. Cadherin 6 is a new RUNX2 target in TGF-β signalling pathway. PLoS One. 2013;8: e75489.

76. Vallath S, Sage EK, Kolluri KK, Lourenco SN, Teixeira VS, Chimalapati S, et al. CADM1 inhibits squamous cell carcinoma progression by reducing STAT3 activity. Sci Rep. 2016;6: 24006.

77. Sakurai-Yageta M, Masuda M, Tsuboi Y, Ito A, Murakami Y. Tumor suppressor CADM1 is involved in epithelial cell structure. Biochem Biophys Res Commun. 2009;390: 977–982.

78. Kim J, Kang HS, Lee Y-J, Lee H-J, Yun J, Shin JH, et al. EGR1-dependent PTEN upregulation by 2-benzoyloxycinnamaldehyde attenuates cell invasion and EMT in colon cancer. Cancer Lett. 2014;349: 35–44.

79. Xu J, Lamouille S, Derynck R. TGF-beta-induced epithelial to mesenchymal transition.

Cell Res. 2009;19: 156–172.

80. Tsukita S, Furuse M. Occludin and claudins in tight-junction strands: leading or supporting players? Trends Cell Biol. 1999;9: 268–273.

81. Zhang Y, Feng XH, Derynck R. Smad3 and Smad4 cooperate with c-Jun/c-Fos to mediate TGF-beta-induced transcription. Nature. 1998;394: 909–913.

82. Barrett CSX, Millena AC, Khan SA. TGF-β Effects on Prostate Cancer Cell Migration and Invasion Require FosB. Prostate. 2017;77: 72–81.

83. Lv Q-L, Huang Y-T, Wang G-H, Liu Y-L, Huang J, Qu Q, et al. Overexpression of RACK1 Promotes Metastasis by Enhancing Epithelial-Mesenchymal Transition and Predicts Poor Prognosis in Human Glioma. Int J Environ Res Public Health. 2016;13. doi:10.3390/ijerph13101021

84. Berndt A, Richter P, Kosmehl H, Franz M. Tenascin-C and carcinoma cell invasion in oral and urinary bladder cancer. Cell Adh Migr. 2015;9: 105–111.

85. Turunen SP, Tatti-Bugaeva O, Lehti K. Membrane-type matrix metalloproteases as diverse effectors of cancer progression. Biochim Biophys Acta Mol Cell Res. 2017;1864: 1974–1988.

86. Jing P, Zhao N, Xie N, Ye M, Zhang Y, Zhang Z, et al. miR-24-3p/FGFR3 Signaling as a Novel Axis Is Involved in Epithelial-Mesenchymal Transition and Regulates Lung Adenocarcinoma Progression. J Immunol Res. 2018;2018: 2834109.

87. Liu J, Li J, Ren Y, Liu P. DLG5 in cell polarity maintenance and cancer development. Int J Biol Sci. 2014;10: 543–549.

88. Zhang X, Song Q, Wei C, Qu J. LRIG1 inhibits hypoxia-induced vasculogenic mimicry

formation via suppression of the EGFR/PI3K/AKT pathway and epithelial-to-mesenchymal transition in human glioma SHG-44 cells. Cell Stress Chaperones. 2015;20: 631–641.

89. Liu X, Li C, Zhang R, Xiao W, Niu X, Ye X, et al. The EZH2- H3K27me3-DNMT1 complex orchestrates epigenetic silencing of the wwc1 gene, a Hippo/YAP pathway upstream effector, in breast cancer epithelial cells. Cell Signal. 2018;51: 243–256.

90. Castel D, Philippe C, Calmon R, Le Dret L, Truffaux N, Boddaert N, et al. Histone H3F3A and HIST1H3B K27M mutations define two subgroups of diffuse intrinsic pontine gliomas with different prognosis and phenotypes. Acta Neuropathol. 2015;130: 815–827.

91. Szenker E, Ray-Gallet D, Almouzni G. The double face of the histone variant H3.3. Cell Res. 2011;21: 421–434.

92. Goldberg AD, Banaszynski LA, Noh K-M, Lewis PW, Elsaesser SJ, Stadler S, et al. Distinct factors control histone variant H3.3 localization at specific genomic regions. Cell. 2010;140: 678–691.

93. Nagaraja S, Quezada MA, Gillespie SM, Arzt M, Lennon JJ, Woo PJ, et al. Histone Variant and Cell Context Determine H3K27M Reprogramming of the Enhancer Landscape and Oncogenic State. Mol Cell. 2019. doi:10.1016/j.molcel.2019.08.030

94. Lin GL, Monje M. A Protocol for Rapid Post-mortem Cell Culture of Diffuse Intrinsic Pontine Glioma (DIPG). J Vis Exp. 2017. doi:10.3791/55360

95. Kim MY, Kaduwal S, Yang DH, Choi KY. Bone morphogenetic protein 4 stimulates attachment of neurospheres and astrogenesis of neural stem cells in neurospheres via phosphatidylinositol 3 kinase-mediated upregulation of N-cadherin. Neuroscience. 2010;170: 8–15.

96. Jones C, Karajannis MA, Jones DTW, Kieran MW, Monje M, Baker SJ, et al. Pediatric high-grade glioma: biologically and clinically in need of new thinking. Neuro Oncol. 2017;19: 153–161.

97. Hargrave D, Bartels U, Bouffet E. Diffuse brainstem glioma in children: critical review of clinical trials. Lancet Oncol. 2006;7: 241–248.

98. Meel MH, Schaper SA, Kaspers GJL, Hulleman E. Signaling pathways and mesenchymal transition in pediatric high-grade glioma. Cell Mol Life Sci. 2018;75: 871–887.

99. Tam WL, Weinberg RA. The epigenetics of epithelial-mesenchymal plasticity in cancer. Nat Med. 2013;19: 1438–1449.

100. Puget S, Philippe C, Bax DA, Job B, Varlet P, Junier M-P, et al. Mesenchymal transition and PDGFRA amplification/mutation are key distinct oncogenic events in pediatric diffuse intrinsic pontine gliomas. PLoS One. 2012;7: e30313.

101. Ott M, Litzenburger UM, Sahm F, Rauschenbach KJ, Tudoran R, Hartmann C, et al. Promotion of glioblastoma cell motility by enhancer of zeste homolog 2 (EZH2) is mediated by AXL receptor kinase. PLoS One. 2012;7: e47663.

102. Vajkoczy P, Knyazev P, Kunkel A, Capelle H-H, Behrndt S, von Tengg-Kobligk H, et al. Dominant-negative inhibition of the Axl receptor tyrosine kinase suppresses brain tumor cell growth and invasion and prolongs survival. Proc Natl Acad Sci U S A. 2006;103: 5799–5804.

103. Yin Y, Qiu S, Peng Y. Functional roles of enhancer of zeste homolog 2 in gliomas. Gene. 2016;576: 189–194.

104. Jolly MK, Mani SA, Levine H. Hybrid epithelial/mesenchymal phenotype(s): The “fittest” for metastasis? Biochim Biophys Acta Rev Cancer. 2018;1870: 151–157.

105. Pan M-R, Hsu M-C, Chen L-T, Hung W-C. Orchestration of H3K27 methylation: mechanisms and therapeutic implication. Cell Mol Life Sci. 2018;75: 209–223.

106. Gröbner SN, Worst BC, Weischenfeldt J, Buchhalter I, Kleinheinz K, Rudneva VA, et al. The landscape of genomic alterations across childhood cancers. Nature. 2018;555: 321–327.

107. Sun L, Fang J. Epigenetic regulation of epithelial-mesenchymal transition. Cell Mol Life Sci. 2016;73: 4493–4515.

108. Vivian J, Rao AA, Nothaft FA, Ketchum C, Armstrong J, Novak A, et al. Toil enables reproducible, open source, big biomedical data analyses. Nat Biotechnol. 2017;35: 314–316.

109. Fang Z. GSEApy.

110. Merico D, Isserlin R, Stueker O, Emili A, Bader GD. Enrichment map: a network-based method for gene-set enrichment visualization and interpretation. PLoS One. 2010;5: e13984.

111. Tirosh I, Izar B, Prakadan SM, Wadsworth MH 2nd, Treacy D, Trombetta JJ, et al. Dissecting the multicellular ecosystem of metastatic melanoma by single-cell RNA-seq. Science. 2016;352: 189–196.

Figure 1

Click here to download Figure Figure1.pdf

A

Enriched in H3K27M differential gene expression

Pathway	p-value
Hallmark Myogenesis	3.29^{-6}
Hallmark UV Response Down	7.16^{-5}
Hallmark KRAS Signaling Down	1.70^{-4}
Hallmark Epithelial Mesenchymal Transition	5.54^{-4}
Hallmark Estrogen Response Late	5.54^{-4}

B

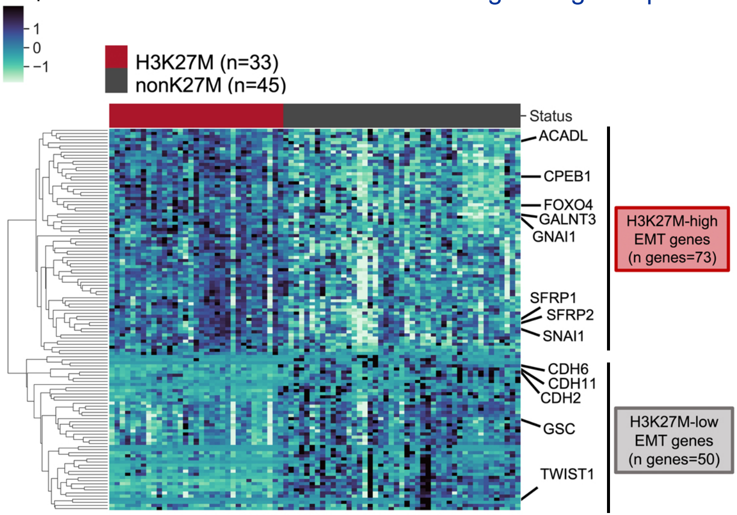

C

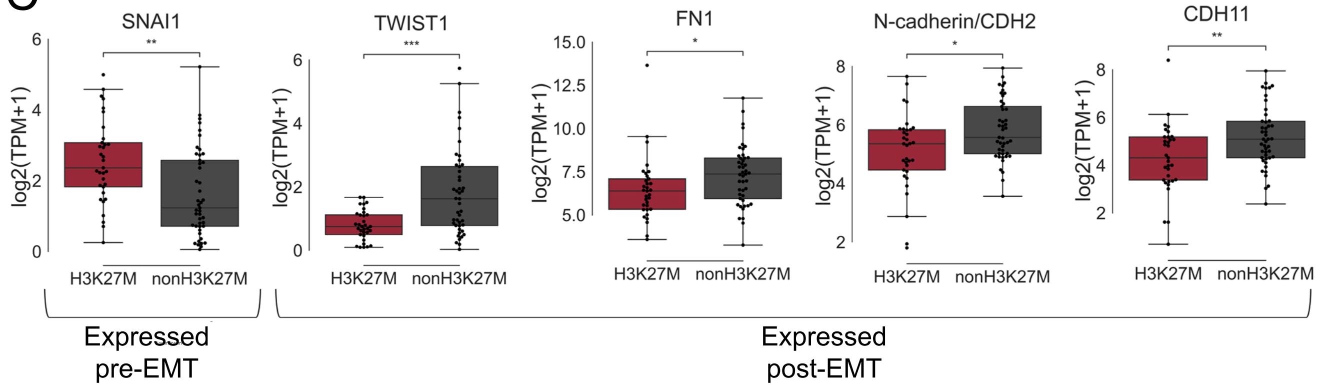

Figure 2

Click here to download Figure Figure2.pdf

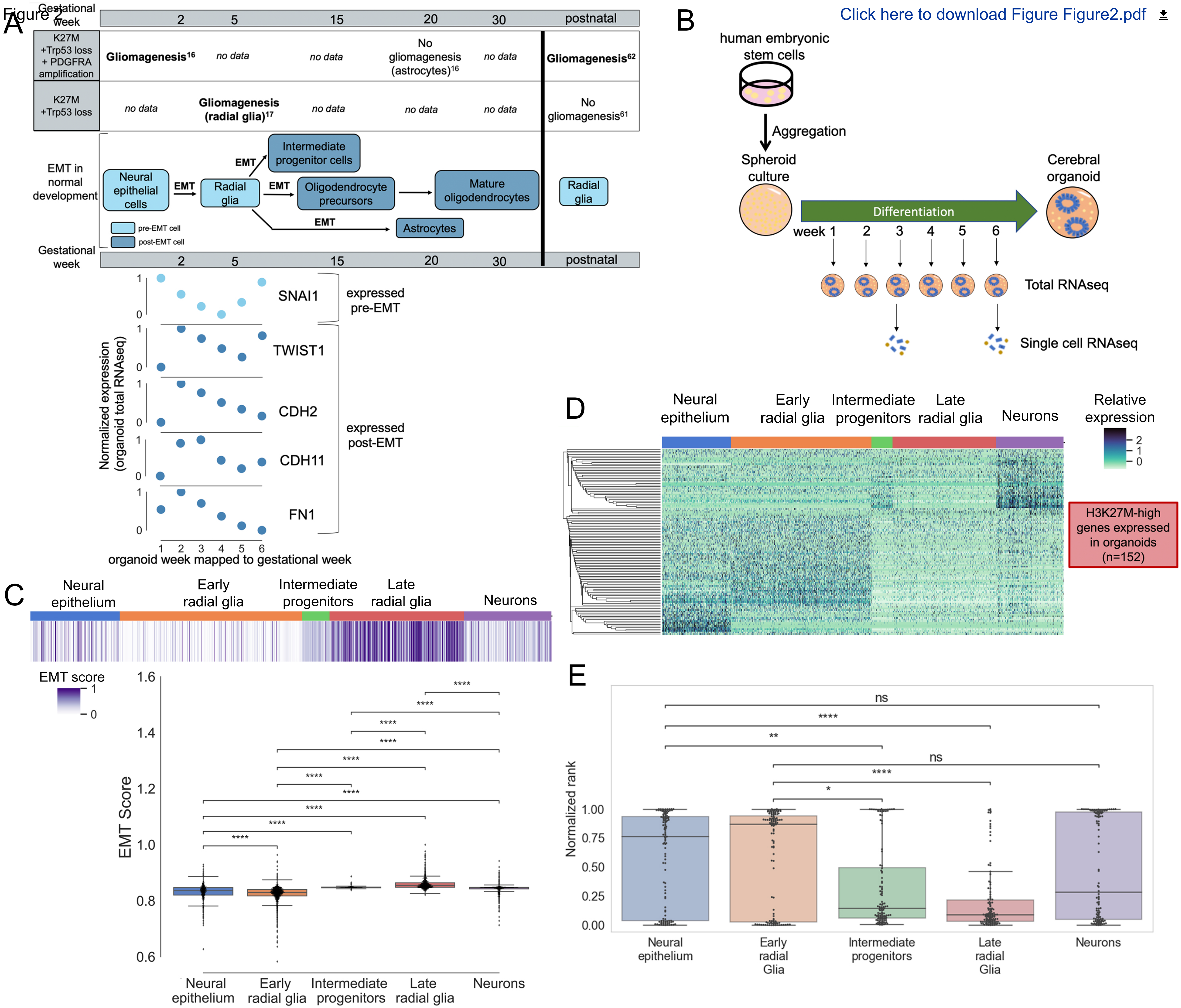

Figure 3

Click here to download Figure Figure3.pdf

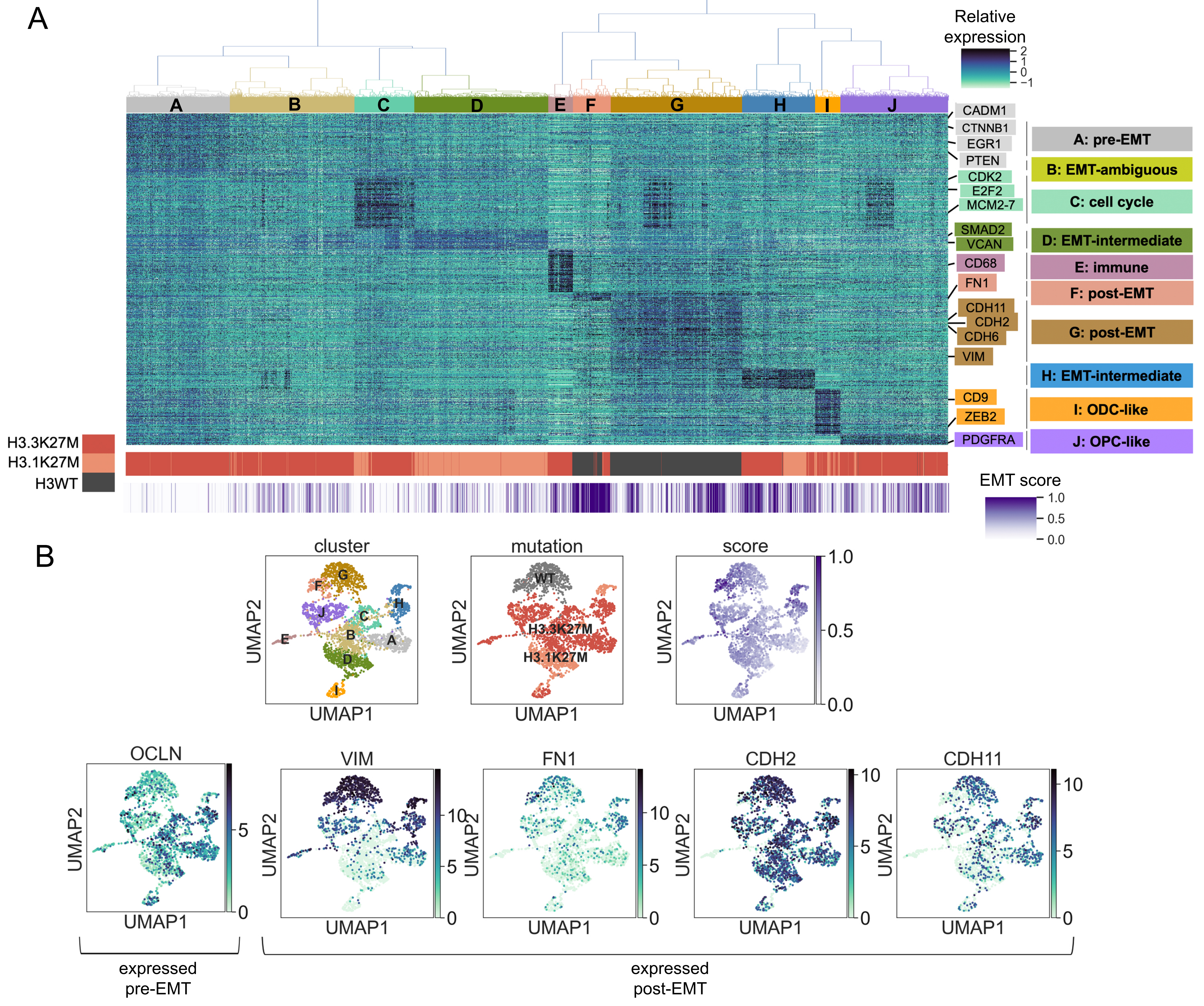

Figure 4

Click here to download Figure Figure4.pdf

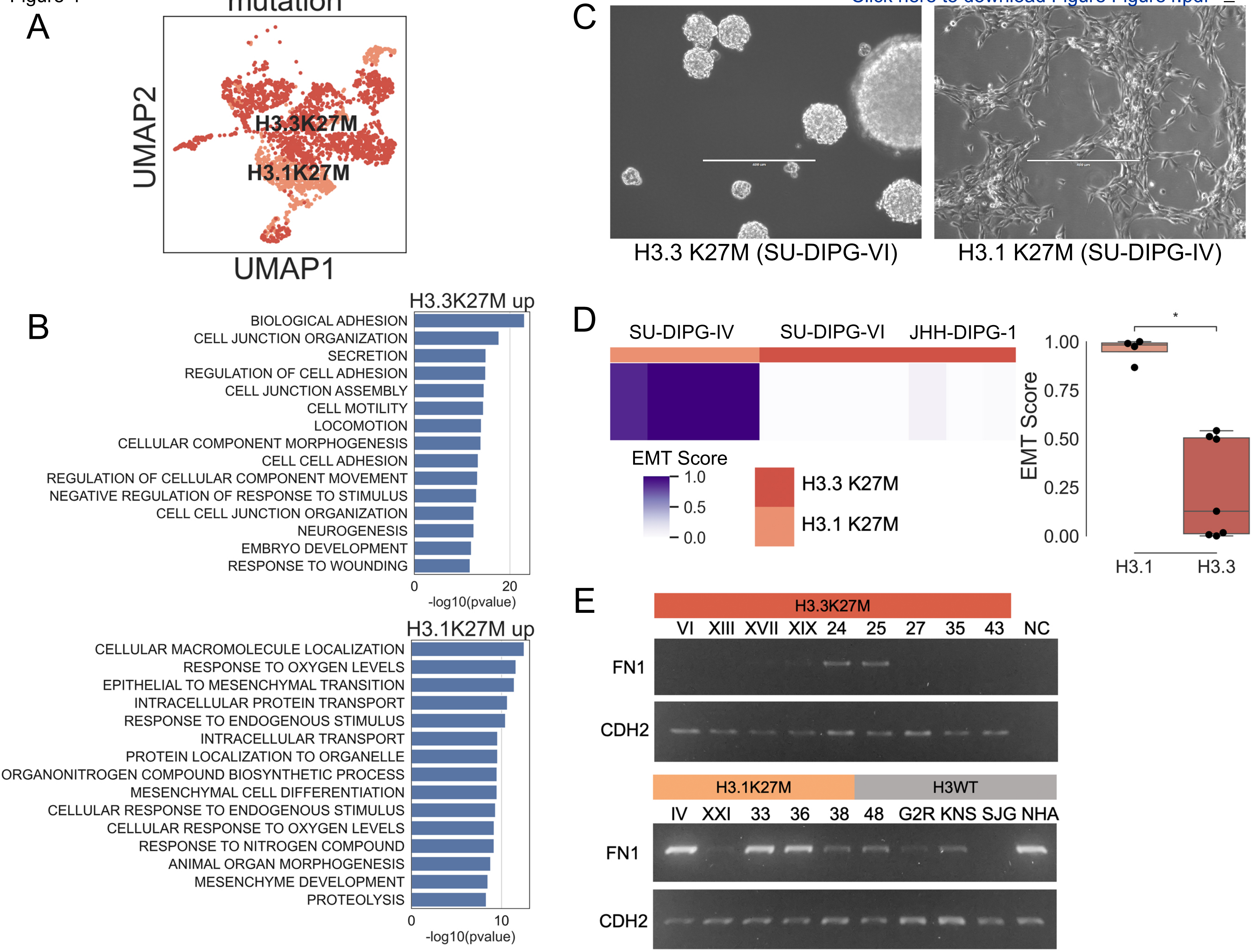

Figure 5

Click here to download Figure Figure5.pdf

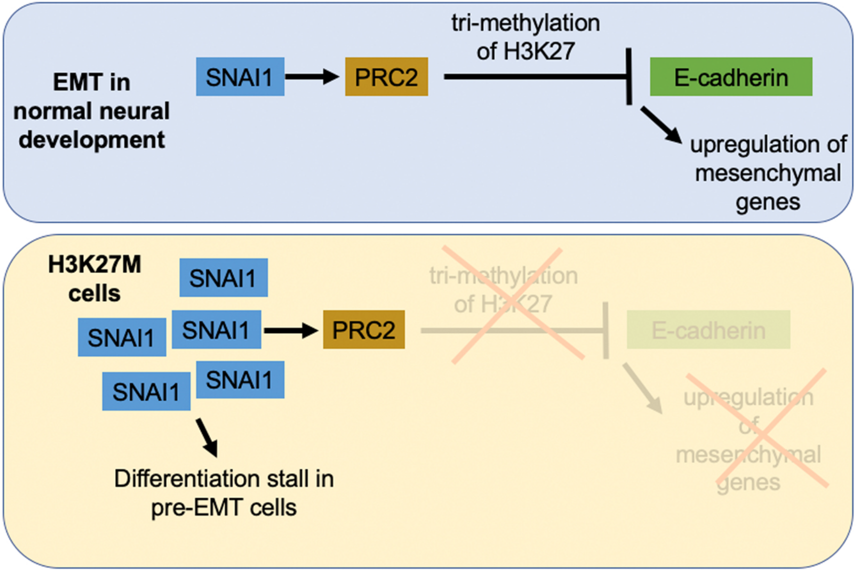

Click here to access/download
Supplementary Material
Supplementary Figures.pdf

Supplementary Table 1

Click here to access/download
Supplementary Material
Supplementary Table 1.xlsx

Supplementary Table 2

Click here to access/download

Supplementary Material

Supplementary Table 2.xlsx

Supplementary Table 3

Click here to access/download
Supplementary Material
Supplementary Table 3.xlsx

Supplementary Table 4

Click here to access/download
Supplementary Material
Supplementary Table 4.xlsx
