# Supplementary material for: Identification of a differentiation stall in epithelial mesenchymal transition in histone H3–mutant diffuse midline glioma
Source: Gigascience. 2020 Dec 15;9(12):giaa136. doi: 10.1093/gigascience/giaa136 (PMC7736793; doi:10.1093/gigascience/giaa136)
Supplement: giaa136_GIGA-D-20-00117_Revision_2 [file giaa136_giga-d-20-00117_revision_2.pdf]

// GigaScience

GigaScience

Identification of a differentiation stall in epithelial mesenchymal transition in histone H3 mutant diffuse midline glioma

--Manuscript Draft--

Manuscript Number:	GIGA-D-20-00117R2
Full Title:	Identification of a differentiation stall in epithelial mesenchymal transition in histone H3 mutant diffuse midline glioma
Article Type:	Research
Funding Information:	American Association for Cancer Research — Dr Olena M Vaske<br>St. Baldrick's Foundation — Dr David Haussler<br>Emily Beazley Kures for Kids Fund — Dr David Haussler<br>Alex's Lemonade Stand Foundation for Childhood Cancer — Dr David Haussler<br>Unravel Pediatric Cancer — Dr David Haussler<br>Team G Childhood Cancer Foundation — Dr David Haussler<br>Live for Others Foundation — Dr David Haussler<br>Schmidt Futures Foundation — Dr David Haussler<br>California Institute for Regenerative Medicine — Dr David Haussler
Abstract:	Background<br>Diffuse midline gliomas with Histone H3 K27M (H3K27M) mutations occur in early childhood and are marked by an invasive phenotype and global decrease in H3K27me3, an epigenetic mark which regulates differentiation and development. H3K27M mutation timing and effect on early embryonic brain development are not fully characterized.<br>Results<br>We analyzed multiple publicly available RNA sequencing datasets to identify differentially expressed genes between H3K27M and nonK27M pediatric gliomas. We found that genes involved in the epithelial-mesenchymal transition (EMT) were significantly overrepresented among differentially expressed genes. Overall, the expression of pre-EMT genes was increased in the H3K27M tumors as compared to nonK27M tumors, while the expression of post-EMT genes was decreased. We hypothesized that H3K27M may contribute to gliomagenesis by stalling an EMT required for early brain development, and evaluated this hypothesis by employing another publicly available dataset of single-cell and bulk RNA sequencing data from developing cerebral organoids. This analysis revealed similarities between H3K27M tumors and pre-EMT normal brain cells. Finally, a previously published single-cell RNA sequencing dataset of H3K27M and nonK27M gliomas revealed subgroups of cells at different stages of EMT. In particular, H3.1K27M tumors resemble a later EMT stage compared to H3.3K27M tumors.<br>Conclusions<br>Our data analyses indicate that this mutation may be associated with a differentiation stall evident from failure to proceed through the EMT-like developmental processes, and that H3K27M cells preferentially exist in a pre-EMT cell phenotype. This study demonstrates how novel biological insights could be derived from combined analysis of several previously published datasets, highlighting the importance of making genomic data available to the community in a timely manner.
Corresponding Author:	Lauren Sanders<br>University of California, Santa Cruz<br>Santa Cruz, CA UNITED STATES
Corresponding Author Secondary Information:	

Powered by Editorial Manager® and ProduXion Manager® from Aries Systems Corporation

**Corresponding Author's Institution:**	University of California, Santa Cruz
**Corresponding Author's Secondary Institution:**	
**First Author:**	Lauren Sanders
**First Author Secondary Information:**	
**Order of Authors:**	Lauren Sanders
	Allison Cheney
	Lucas Seninge
	Anouk van den Bout
	Marissa Chen
	Holly C Beale
	Ellen Towle Kephart
	Jacob Pfeil
	Katrina Learned
	A Geoffrey Lyle
	Isabel Bjork
	David Haussler
	Sofie R Salama
	Olena M Vaske
**Order of Authors Secondary Information:**	
**Response to Reviewers:**	Reviewer #1:<br>Well written manuscript exploring the gene expression profile differences between H3K27M mutated and non-mutated paediatric high grade glioma. Results suggest that H3K27M mutant pHGG have a pre-EMT gene expression phenotype in comparison to non-mutant tumours. Some queries below.<br><br>Differential expression analysis:<br>1. Could it be stated somewhere in the manuscript that some of the samples used for differential expression analysis are not strictly paediatric and were rather aged <30.<br>Response:<br>We have included this statement on page 7.<br><br>2. Were the p-values adjusted for multiple testing?<br>Response:<br>Yes, a Benjamini Hochberg multiple testing correction was applied; we have included this in the Methods on page 29.<br><br>Pan-disease outlier analysis<br>3. It was unclear which types of cancers were included in the background tumours for this analysis.<br>Response:<br>The background cohort for the pan-disease outlier analysis consisted of 37 pediatric gliomas, 19 young adult gliomas, 18 pediatric glioblastomas and 4 young adult glioblastomas. We have included this information in the Methods (page 26).<br><br>RT-PCR<br>4. please provide annealing temperatures for each primer pair<br>Response:<br>The annealing temperature for both primer pairs was 64C. We have included this in the Methods on page 29.<br><br>Figure 2A

Powered by Editorial Manager® and ProduXion Manager® from Aries Systems Corporation

5. It's not super clear that the previous finding of gliomagenesis in the postnatal period was identified in a pre-EMT cell type, and the finding of no-gliomagenesis occurred in a post-EMT cell type. Could it be made clearer in the figure what cell types these previous experiments were performed in?
Response:
To clarify, Lewis et al., Science 2013 (ref. 62) found that expression of H3.3K27M with p53 loss in neonatal progenitor cells was insufficient for gliomagenesis. Subsequently, Larson et al. Cancer Cell 2019 (ref. 63) found that induction of H3.3K27M, p53 loss and mutant PDGFRA in neonatal progenitor cells did result in spontaneous gliomagenesis. Both experiments involved pre-EMT progenitor cells. We have clarified this in the text on page 12.

EMT gene signature and score
6. Could you please provide more information on how this was developed and calculated.
Response:
The EMT completion gene signature was developed through literature review and contains canonical post-EMT gene markers. Supplementary Table 3 shows the genes in this signature and literature sources for the role of each gene in EMT. The single cell transcriptional score is calculated using a previously published method (Tirosh et al., Science 2016, Filbin et al., Science 2018, and Neftel et al., Cell 2019). A control gene set 100 times the length of the gene set of interest is produced by binning all genes into 30 bins of aggregate expression levels. For each gene in the gene set of interest, 100 genes are randomly selected from that gene's expression bin. This creates a control gene set with a comparable expression distribution, so that its expression provides a background gene set that is 100 times larger than the gene set of interest. For each cell, the average expression of the gene set of interest is compared to the average expression of the control gene set to create a raw score for that cell. The scores are then normalized across the dataset. The code for our implementation of this method is publicly available at: github.com/lauren-sanders/EMT-paper/

Figure 3A
7. Would it be possible to include which of the 8 individual gliomas each cell is from as an additional coloured bar? It would be interesting to see if the cells clustered by patient as well as mutation.
Response:
We have included this bar in Figure 3A.

8. Additionally could you provide the number of cells sequenced and analysed per glioma for this figure.
Response:
The number of cells per glioma is below and we have added this information to the Methods (page 27).
MUV1: 146
MUV5: 708
MUV10: 286
BCH836: 527
BCH869: 492
BCH1126: 299
MGH66: 442
MGH101: 92
MGH104: 65

Line 92
9. I think there may be a word missing in this line: "resulting in H3K27 trimethylation of key epithelial genes such as concurrently upregulating mesenchymal genes"
Response:
Thank you; we have reworded the sentence.

Reviewer #2:
The authors searched a subset of publicly available pediatric high-grade gliomas datasets to compare differentially expressed genes between H3K27M mutant diffuse gliomas and H3K27M wild-type gliomas. They identified an early EMT signature

overexerted in the former compared to the latter.
MAJOR COMMENTS
1. Diffuse midline glioma whether H3K27M mutant are different from hemispheric glioma especially in their invasive phenotype. As such, comparing these entity is proved to show a difference in EMT as invasion and EMT are so linked. Therefore, the authors can not infer that the difference shown here is due to the H3K27M mutation per se. To support this statement, they should either perform KO or KD experiments in H3K27M mutant cells or KI experiments in H3K27M wild-type cells. Alternatively, separating in the data set diffuse gliomas from the more circumscribed gliomas would be helpful. Ideally a significant data set from diffuse gliomas without H3K27M mutation may be a better control. If these experiments can not be performed, the statement should be then softened.
Response:
We thank the reviewer for raising a very important point and providing some excellent recommendations for additional analysis. In order to address this issue, we have repeated our differential expression analysis with a cohort of DIPG samples containing no hemispheric glioma samples, in order to clarify the role of H3K27M mutation after removing possible histological or location factors. The results of this analysis are consistent with the original findings. We describe this analysis on page 10 of the manuscript, and we also provide a caveat in the Discussion (page 24) and suggest future studies that could more clearly describe the role of H3K27M mutation alone in the observed EMT-related transcriptional signature. We provide the description of the additional DIPG-specific analysis, as well as the caveat in the Discussion, below:

Additional DIPG-specific Analysis: (page 10)
Finally, because EMT is associated with invasiveness in gliomas, and diffuse midline glioma are by nature more invasive than hemispheric glioma, we performed an additional analysis restricted to diffuse intrinsic pontine glioma (DIPG) to elucidate the role of the H3K27M mutation in the observed EMT-related transcriptional profiles. The goal of this analysis was to remove any potential histological or location signal that may be influencing the EMT-related gene expression. We used 10 H3 wild-type DIPG samples and 47 H3K27M DIPG samples from Treehouse cancer compendium v11. Limma differential expression analysis revealed 48 genes with higher expression in H3K27M DIPG compared to nonK27M DIPG (Supplementary Table 2). We again computed statistical overlap of these genes with 9 gene sets relating to epithelial cells and early brain development, and found significant overlap with 4 of the 9 gene sets (pvalue<0.1, Supplementary Table 2). The enrichment of 48 randomly selected genes in these 9 gene sets were not significant (Supplementary Table 2).

Caveat in the Discussion (page 24):
“Additionally, a limitation of our study is that it is difficult to isolate the role of the H3K27M mutation from other factors such as histology and tumor location. Future studies will focus on EMT-related transcriptional programs in cellular models with inducible H3K27M expression to further characterize the molecular interplay between the H3K27M mutation and EMT in developing brain cells.”

2. A comparison with adult type gliomas of the mesenchymal subtype would be interesting as well to tease out the real role of H3K27M mutation in this phenotype.

Response:
We examined the expression of the adult glioma mesenchymal subtype gene signature (Verhaak et al., Cancer Cell 2010) in our pHGG cohort (Reviewer Requested Figure 1). The H3K27M and nonK27M pHGG samples display similar expression of the adult mesenchymal signature, such that the expression of these genes does not separate them into different subtypes. This may be explained by the numerous differences between adult glioblastomas and pediatric diffuse midline gliomas. Adult glioblastomas have a higher mutation rate than pediatric gliomas (Schwartzentruber et al., Nature 2012), and the vast majority are supratentorial (Jones and Baker, Nat Rev Cancer 2014), while by definition DMGs occur along the midline. Additionally, treatments that have shown efficacy in adult GBM are notably ineffective in DMGs (Jones et al., Neuro Oncol 2016).

Additionally, the adult GBM Mesenchymal subtype is predominantly characterized by mutations or deletions of NF1, a gene which is rarely altered in pediatric glioma.

Powered by Editorial Manager® and ProduXion Manager® from Aries Systems Corporation

Moreover, this subtype expresses a combination of mesenchymal and astrocytic markers linked to dedifferentiated tumors. While dedifferentiation is well characterized in adult tumors since they arise in fully developed tissues, pediatric gliomas arise during embryonic development and are thought to represent a differentiation stall rather than dedifferentiation events (Filbin and Monje, Nat Med 2019).

Also, while the EMT is associated with proliferation and invasion in some adult cancers (Marcucci et al., Nat Rev Drug Discov 2016), here we refer to the EMT as a developmental process which must occur multiple times during normal neuronal development. This process is controlled by the H3K27me3 epigenetic mark, and we hypothesize that lack of H3K27me3 may prevent full completion of EMT at one or multiple timepoints during early brain development. Here, we follow up on previous work in the field which shows that early neural or glial precursor cells are in a susceptible transcriptional state and undergo a differentiation stall upon the occurrence of H3K27M. Because embryonic neural progenitor/stem cells undergo EMT-like processes during the formation of the neural plate and neural tube, we hypothesized that early neural or glial precursors poised to undergo an EMT as part of normal development are in a susceptible transcriptional state for gliomagenesis. Importantly, here we discuss EMT as a normal developmental process implicated in the early stages of H3K27M gliomagenesis, separate and distinct from the EMT as a process utilized by malignant cells undergoing metastasis, which may also occur later in pHGG tumor growth, as noted in Puget et al., PLoS One 2012.

We have clarified this throughout the manuscript by removing the use of the words “epithelial” or “mesenchymal” when referring to the EMT-like pHGG phenotypes that we observe.

3. The concept of EMT in gliomas can not be extrapolated form the data acquired in epithelial tumors. Most glioma gene expression profiles are mesenchymal and not epithelial at all while the so called mesenchymal subtype is even more mesenchymal (Iser et al, Med Res Rev 2017). The term is therefore misleading, should be used with caution and the EMT datasets from epithelial cancers should not be applied. The genes defining mesenchymal subset of GBM may have been more appropriate.

Response:
Thank you to the reviewer for an excellent point. To address this comment, we have removed genes derived from EMT datasets from epithelial cancers, and retain only genes from datasets annotated by EMT as a cellular developmental process. This change is reflected in Supplementary Table 2 “EMT master gene list” and Figures 1B and 3A, as well as in the text on page 8. As discussed in point 2, the genes defining the mesenchymal subtype of adult GBM may be less relevant for studying EMT as a developmental process in the brain.

MINOR COMMENTS
- Differential expression analysis of pediatric gliomas with and without H3K27M mutation reveals deregulation of genes involved in epithelial-mesenchymal transition.
4. The authors should provide a reference that indicate that EMT is orchestrated by H3K27me3 marks (I found one reference in glioma Zhou et al, Oncol Lett 2019, the rest is in epithelial cancers).
Response:
Thank you to the reviewer for identifying an excellent reference. We have included this reference on page 4.

5. The limitation of GSEA datasets is that in most of the cases they are not oriented by over-expression or repression of the gene in the described pathway.
Response:
In order to overcome this limitation, we performed differential expression analysis first, then used gene set enrichment analysis to investigate gene sets enriched in overexpressed genes in the H3K27M glioma cohort.

6. The authors claim that over expressed genes in H3K27M mutant genes are associated with epithelial-like states and normally unregulated prior EMT while those

Powered by Editorial Manager® and ProduXion Manager® from Aries Systems Corporation

under expressed are associated with the mesenchymal state, this is in contradiction with the adult literature in gliomas and also previous publications (Meel et al, Cell Mol Life Sci 2018 or Puget et al, PLoS ONE 2012). They should provide statistically meaningful data and not only exemples.

Response:
In order to provide statistically meaningful analysis of the genes over-expressed in H3K27M mutant gliomas, we performed additional gene set enrichment analysis using the EMT genes over-expressed in H3K27M mutant glioma. Our results are consistent with the rest of our original findings, and show significant enrichment in gene sets related to early development, neurodevelopment, epithelial proliferation and epithelium development. We have included these results in the manuscript on page 9.

With respect to the publications mentioned by the reviewer, Meel et al 2018 primarily reviews the data on EMT in adult gliomas; there is limited data on the expression of EMT genes in DIPGs. They also acknowledge differences in the role of EMT genes between adult gliomas and DIPGs. In figure 4 of their paper, the authors show that in 6/8 pro-EMT genes examined, their expression in DIPGs is lower than or equal to the expression in adult GBM. Those results concur with our data in H3K27M gliomas. In addition, Meel et al specifically calls for more research in EMT in pediatric gliomas, which is the focus of our study.

Puget 2012 identifies a subset of DIPG samples (whose H3K27M status is unknown) that is enriched for the mesenchymal adult GBM subtype (mentioned above). They also examine expression of a list of 53 transcription factors associated with EMT in adult HGGs. It appears from figure 3A that some portion of these genes has relatively low expression in this mesenchymal subset of DIPGs, which is consistent with our analysis as well. While Puget was crucial in discovering a role for EMT in DIPGs, our study provides a novel contribution by focusing on the role of H3K27M and the concomitant loss of H3K27me3 in the expression of EMT-related genes.

We discuss both of these previous studies in our manuscript (Meel et al 2018 referenced on page 22, Puget 2012 referenced on pages 20 and 21 as discussed in points 12 and 14).

- H3K27M-mediated gliomagenesis is associated with pre-EMT cell types.

7. If I understood the experiment correctly, the authors show that H3K27M mutant gliomas have a pre-EMT signature and that this signature is present in the developing brain in cells in pre-EMT stage. This is a rather tautologic demonstration. EMT and stem cell state are really correlated in these gliomas where the bulk of the tumor cells are stem-like cells (Filbin et al, Science 2018). This could well be another hypothesis. This organoid experiment does not clarify it in my view.
Response:
To clarify, the organoid experiment serves to identify specific cell stages in the developing brain which are transcriptionally similar to H3K27M glioma. We employed the organoid data in order to investigate the hypothesis that H3K27M gliomas are similar to pre-EMT developing cells, and this analysis shows a transcriptional similarity between H3K27M gliomas and pre-EMT brain cell stages. We have clarified this experiment in the text on page 14.

- Single-cell profiling of H3K27M gliomas reveals groups of cells at different stages of EMT.
8. Number of samples are really low and lower than in the the reference paper where this data was extracted from, why?
Response:
The reference paper analyzed 6 K27M gliomas and 3 IDH-WT / H3-WT GBMs, all of which were publicly available at GSE102130 and we have included all of these data in our analysis. Although the reference paper does reference additional IDH-mutant astrocytoma and IDH-mutant oligodendroglioma samples, these data were not publicly released and would not have been relevant for our analysis.

9. How do the A, F, G, H clusters compare with the stemless signature used by Filbin et al?

Response:
We examined the expression of the 4 signatures identified by Filbin et al., Science 2018 (cell cycle, astrocytic, oligodendrocytic, and OPC-like) in the clusters identified in the EMT analysis. Consistent with our analysis, the mature astrocytic signature is enriched in “post-EMT” clusters A and B. The mature oligodendrocytic signature is enriched in cluster H, consistent with our finding that cluster H is ODC-like. The oligodendrocyte precursor stem-like signature is enriched in cluster E, consistent with our finding that cluster E is less differentiated and “pre-EMT”, and and in “OPC-like” cluster G. We include this figure in the manuscript as Supplementary Figure 5.

10. The occurence of tumor cells in different state of EMT is in contradiction with the claim that H3K27M tumors are in a differentiation stall, isn't?
Response:
The single cell RNA-seq analysis refines the hypothesis that H3K27M tumors undergo a differentiation stall during developmental EMT events. While both H3.3K27M and H3.1K27M cells appear to be less mature than H3WT cells, the two mutations result in slightly different EMT-related transcriptional signatures, with H3.3K27M cells expressing a more primitive signature than H3.1K27M cells.

11. The mixing of different H3K27M tumors is not appropriate, especially with the many differences already shown between H3.1 and H3.3K27M tumors (Nagaraja, Castel for exemple).
Response:
There are indeed many known differences between H3.1K27M and H3.3K27M tumors as noted by the reviewer. However, for our study, we started out by hypothesizing that both H3.1K27M and H3.3K27M mutant gliomas would be different from the H3WT tumors because they both have loss of H3K27 trimethylation. Since we included both mutation types and wild-type cells in our unsupervised single cell clustering, we were able to identify different transcriptional signatures which would not have been apparent if we had kept the mutation types separate.

- Histone H3.1K27M glioma cells may represent a more advanced stage of EMT than H3.3K27M glioma cells.
12. EMT has been observed in DIPG (the prototypic H3K27M diffuse midline glioma) since 2012 by Puget et al. Albeit the histone H3 mutation was not known. The mesenchymal subtype described in this paper resembles the H3.1K27M subgroup mentioned here. This may be discussed later. Moreover, the H3.1K27M DIPG gene expression profile has been already shown to be enriched with the mesenchymal GBM signature, this old data support their finding and should not be omitted in the discussion.
Response:
Thank you to the reviewer for the reminder of two very relevant studies. We have included a reference of the EMT-related findings of Puget et al (PLoS One 2012) and Castel et al (Acta Neuropathol 2015) on page 21. The new text is below:
“Our computational and in vitro observations are consistent with a recent study indicating that H3.1K27M tumor cells are overall more differentiated than H3.3K27M tumor cells (Nagaraja et al., Mol Cell 2019). Our results are also consistent with previous studies on EMT in pediatric gliomas which first found a mesenchymal subtype of DIPG and subsequently discovered that H3.1K27M mutant gliomas express genes associated with a more mesenchymal subtype of glioblastoma(Puget et al., PLoS One 2012, Castel et al., Acta Neuropathol 2015).”

13. There is also data out there showing a different distribution of H3K27me3 marks in DIPG with respect to the histone H3 variant mutated at K27 (Castel et al, Acta Neuropathol Comm 2018) and this data is not in line with the statement that in H3.1K27M induced H3K27me3 loss is diffuse. Please comment or amend.
Response:
We have amended our description of the trimethylation loss in the two mutation types on page 19 to be consistent with the findings of Castel et al, Acta Neuropathol Comm 2018, and we have included a reference to this paper. The amended text is below:
“Normally, histone H3.3 is preferentially located at active chromatin (Szenker et al., Cell Res 2011, Goldberg et al., Cell 2010, Nagaraja et al., Mol Cell 2019). This leads to distinct patterns of epigenetic reprogramming in each histone variant, where loss of the H3.3K27me3 mark is directly correlated with areas of H3.3 genomic enrichment, while

Powered by Editorial Manager® and ProduXion Manager® from Aries Systems Corporation

	H3.1K27me3 loss is higher at intergenic regions (Nagaraja et al., Mol Cell 2019, Castel et al., Acta Neuropathol Commun 2018).”<br><br>14. The single cell data comparing H3.1 and H3.2K27M cells confirms the previous finding of Castel et al (Acta Neuropathol 2015). Why did the authors not consider these findings?<br>Response:<br>Thank you to the reviewer for the suggestion and reference. As noted in point 12, we have included a discussion of the findings of Castel et al (Acta Neuropathol 2015) on page 21.<br><br>Discussion<br>15. In Grasso's paper (Nature Med 2015), it was shown that HDACi treatment was reverting the mesenchymal phenotype of the tumors cells in vitro. The authors should comment this information as well since it is supporting the interplay between H3K27Ac/H3K27me3 and mesenchymal drift.<br>Response:<br>Thank you to the reviewer for the suggestion. We now include a discussion of the findings of Grasso et al., Nature Med 2015 on page 23. The new text is below:<br>“Histone deacetylase inhibitor treatment of in vitro pHGG cells reversed mesenchymal phenotypes, in keeping with a model in which the interplay between H3K27 acetylation and methylation controls EMT-related transcriptional states(Grasso et al., Nature Med 2015).”
**Additional Information:**	
**Question**	**Response**
Are you submitting this manuscript to a special series or article collection?	No
**Experimental design and statistics**<br><br>Full details of the experimental design and statistical methods used should be given in the Methods section, as detailed in our Minimum Standards Reporting Checklist. Information essential to interpreting the data presented should be made available in the figure legends.<br><br>Have you included all the information requested in your manuscript?	Yes
**Resources**<br><br>A description of all resources used, including antibodies, cell lines, animals and software tools, with enough information to allow them to be uniquely identified, should be included in the Methods section. Authors are strongly encouraged to cite Research Resource Identifiers (RRIDs) for antibodies, model organisms and tools, where possible.	Yes

Have you included the information requested as detailed in our Minimum Standards Reporting Checklist?	
**Availability of data and materials**<br><br>All datasets and code on which the conclusions of the paper rely must be either included in your submission or deposited in publicly available repositories (where available and ethically appropriate), referencing such data using a unique identifier in the references and in the “Availability of Data and Materials” section of your manuscript.<br><br>Have you have met the above requirement as detailed in our Minimum Standards Reporting Checklist?	Yes

Powered by Editorial Manager® and ProduXion Manager® from Aries Systems Corporation

Manuscript

Click here to access/download;Manuscript;EMT Paper Giga Science Final Sub 110220.pdf

Identification of a differentiation stall in epithelial mesenchymal transition in histone H3 mutant diffuse midline glioma

Lauren M. Sanders[1,4*#$], Allison Cheney[2#], Lucas Seninge[1,4], Anouk van den Bout[2,4], Marissa Chen[2,4], Holly C. Beale[2,4], Ellen Towle Kephart[4], Jacob Pfeil[1,4], Katrina Learned[4], A. Geoffrey Lyle[2,4], Isabel Bjork[4], David Haussler[1,3,4], Sofie R. Salama[1,3,4+], Olena M. Vaske[2,4+]

[1]Department of Biomolecular Engineering, [2]Department of Molecular, Cell and Developmental Biology, [3]Howard Hughes Medical Institute, [4]University of California Santa Cruz Genomics Institute, University of California Santa Cruz, 1156 High Street, Santa Cruz, CA, USA, 95064

*Corresponding author

#Co-first author

+Co-senior author

$ L.M.S.'s current affiliation is Department of Molecular, Cell and Developmental Biology, University of California Santa Cruz.

Author Email Addresses

Lauren M. Sanders lmsh@ucsc.edu; Allison Cheney archeney@ucsc.edu; Lucas Seninge lseninge@ucsc.edu; Anouk van den Bout anvanden@ucsc.edu; Marissa Chen marissaamberchen@gmail.com; Holly C. Beale hcbeale@ucsc.edu; Ellen Towle Kephart ekephart@ucsc.edu; Jacob Pfeil <jpfeil@ucsc.edu; Katrina Learned klearned@ucsc.edu; A. Geoffrey

Lyle aglyle@ucsc.edu; Isabel Bjork ibjork@ucsc.edu; David Haussler haussler@ucsc.edu; Sofie R. Salama ssalama@ucsc.edu; Olena M. Vaske olena@ucsc.edu

Corresponding Author

Lauren M. Sanders

1156 High Street, 220 Sinsheimer Labs

University of California Santa Cruz

Santa Cruz, CA 95060 USA

phone: (530) 409 2174

lmsh@ucsc.edu

ORCIDs:

Lauren M. Sanders, 0000-0001-9393-0861;

Allison Cheney, 0000-0003-1435-6190;

Lucas Seninge, 0000-0001-6536-6810;

Anouk van den Bout, 0000-0001-8769-6853;

Marissa Chen, 0000-0002-8882-6004;

Holly C. Beale, 0000-0003-4091-538X;

Jacob Pfeil, 0000-0002-8773-8520;

A. Geoffrey Lyle, 0000-0002-3435-526X;

David Haussler, 0000-0003-1533-4575;

Sofie R. Salama, 0000-0001-6999-7193;

Olena Vaske, 0000-0002-1677-417X

Abstract

Background

Diffuse midline gliomas with Histone H3 K27M (H3K27M) mutations occur in early childhood and are marked by an invasive phenotype and global decrease in H3K27me3, an epigenetic mark which regulates differentiation and development. H3K27M mutation timing and effect on early embryonic brain development are not fully characterized.

Results

We analyzed multiple publicly available RNA sequencing datasets to identify differentially expressed genes between H3K27M and nonK27M pediatric gliomas. We found that genes involved in the epithelial-mesenchymal transition (EMT) were significantly overrepresented among differentially expressed genes. Overall, the expression of pre-EMT genes was increased in the H3K27M tumors as compared to nonK27M tumors, while the expression of post-EMT genes was decreased. We hypothesized that H3K27M may contribute to gliomagenesis by stalling an EMT required for early brain development, and evaluated this hypothesis by employing another publicly available dataset of single-cell and bulk RNA sequencing data from developing cerebral organoids. This analysis revealed similarities between H3K27M tumors and pre-EMT normal brain cells. Finally, a previously published single-cell RNA sequencing dataset of H3K27M and nonK27M gliomas revealed subgroups of cells at different stages of EMT. In particular, H3.1K27M tumors resemble a later EMT stage compared to H3.3K27M tumors.

Conclusions

Our data analyses indicate that this mutation may be associated with a differentiation stall evident from failure to proceed through the EMT-like developmental processes, and that H3K27M cells preferentially exist in a pre-EMT cell phenotype. This study demonstrates how

novel biological insights could be derived from combined analysis of several previously published datasets, highlighting the importance of making genomic data available to the community in a timely manner.

Keywords

Glioma, H3K27M mutation, epithelial mesenchymal transition

Background

Pediatric high grade gliomas (pHGGs) are aggressive brain tumors occurring at a median age of 6[1]. Sixty percent of pHGGs harbor a histone H3 K27M mutation, which is associated with an aggressive phenotype and dismal survival rates[2]. H3K27M-mutant pHGG tumors are located along the midline, including in the pons, cerebellum, and brainstem. A diffuse phenotype and delicate location leave them unsuitable for surgery, and their pronounced chemoresistance renders the standard treatments for gliomas ineffective, resulting in a median survival time of only 12 months[3,4]. The prognostic significance of the H3 K27M mutation in these gliomas resulted in a new WHO tumor classification, diffuse midline glioma with H3K27M mutation[5]. The H3K27M mutation results in a global decrease in H3K27me3, an epigenetic repressive mark and posttranslational histone modification[6]. Seventy five percent of gene loci lose or have reduced H3K27me3, although a few loci gain the mark as a result of the H3K27M mutation[2,7]. H3K27me3 is deposited predominantly by EZH2, the catalytic subunit of the PRC2 methyltransferase complex. By regulating H3K27me3, EZH2 maintains cell identity and regulates cellular differentiation[8–11]. Silencing EZH2 in neuroepithelial cells before their differentiation alters the distribution of the progeny cell types[12]. EZH2 also maintains neuroepithelial cell integrity, and midbrain identity[13,14].

Because H3K27me3 is globally lost in H3K27M-mutant glioma, the subsequent deregulation of gene expression is thought to lead to tumorigenesis, although the developmental timing of the mutational event is important[15]. H3K27M expression in neural stem cells has led to tumorigenesis in mice when accompanied by *TP53* knockout and/or *PDGFRA* amplification, but this combination of molecular aberrations failed to result in tumorigenesis when introduced in mature astrocytes[16,17]. However, the precise cell type of origin for H3K27M gliomas is not yet known. Candidate cell types include neuroepithelial cells (also known as neural stem cells), radial glia (also known as neural progenitor cells), and oligodendrocyte precursor cells (OPCs)[16–18].

Many important brain developmental processes are regulated by H3K27me3 deposition and could contribute to gliomagenesis if not well controlled. One of these is the epithelial-mesenchymal transition (EMT) pathway, which is essential for gastrulation, migration of neural crest cells, and neural tube formation[19–22]. ~~The~~ EMT is regulated by SNAI1, a transcription factor master regulator[23–25]. By regulating EMT, SNAI1 plays a critical role in many developmental processes, including gastrulation and differentiation of embryonic stem cells[26–28]. SNAI1 induces EMT through direct recruitment of PRC2, resulting in H3K27 trimethylation of key epithelial genes ~~such~~ as well as concurrently upregulating mesenchymal genes[29,30].

In the brain, cellular transitions driven by EMT-like transcriptional programs are involved in key developmental steps such as the differentiation of neuroepithelial cells to both neuronal and glial cells[31,32]. These transitional transcriptional programs, which control cell fate and identity in early neural cell development, are regulated by EZH2[33].

Given the regulation of EMT-associated gene transcription by H3K27me3 deposition in the brain, and the disruption of this deposition by the H3K27M mutation, we sought to investigate

EMT-related gene expression in pHGGs with and without the H3K27M mutation. We analyzed RNA sequencing data from 78 pHGGs obtained from several different studies (Supplementary Table 1). First, we performed differential expression analysis using RNA sequencing (RNA-seq) derived gene expression from bulk tumor samples, and found that H3K27M gliomas differentially express pre-EMT genes[34]. Secondly, we examined previously published cerebral organoid data and observed transcriptional similarities between pre-transition neural stem cells and H3K27M gliomas[35]. Finally, we leveraged a recent single cell RNA sequencing dataset to uncover multiple EMT-related transcriptional states in H3K27M tumor cells[18]. Overall, our results suggest that the H3K27M mutation may cause an arrest in development of a neural stem cell type due to lack of H3K27me3 transcriptional control of EMT-related cellular transitions, indicating a developmental window of opportunity for H3K27M mutations to induce gliomagenesis.

Our study highlights the importance of genomic data sharing for rare diseases, such as pHGGs. By combining RNA sequencing data from multiple previously published studies, we were able to assemble a cohort of 78 pHGG, large enough for the differential expression analysis of pHGGs with and without the H3K27M mutation. We used this new cohort of previously published data to derive a novel biological model to describe the molecular pathogenesis of the disease.

Data Description

The RNA sequencing data from bulk clinical pediatric glioma samples used in these analyses were downloaded from the Treehouse cancer compendium v8, which is publicly available at the Treehouse website (treehousegenomics.soe.ucsc.edu/public-data/). All samples passed the RNA sequencing quality control analysis used in the curation of the Treehouse cancer compendium[34]. The single cell glioma RNA sequencing data were downloaded from the Gene Expression

Omnibus (accession: GSE102130), where it is publicly available. The dataset was log-normalized and filtered for low expression and low variability genes. The RNA sequencing data from glioma cell lines were accessed with permission from dbGap phs000900.v1.p1, where it is available to other researchers with permission, and all samples passed the RNA sequencing quality control analysis used in the curation of the Treehouse cancer compendium[34]. The bulk and single cell organoid RNA sequencing data were downloaded from the Gene Expression Omnibus (accession: GSE106245), which is publicly available. The datasets were log-normalized and filtered for low expression and low variability genes.

Analyses

A. Differential expression analysis of pediatric gliomas with and without H3K27M mutation reveals deregulation of genes involved in epithelial-mesenchymal transition.

We obtained RNA-seq data from 33 H3K27M pediatric/young-adult (ages 0-29) high grade gliomas (pHGG) and 45 nonK27M pHGG from the Treehouse Childhood Cancer Initiative public cancer compendium v8[36] (Supplementary Table 1). These data came from several cohorts including the Pacific Pediatric Neuro-Oncology Consortium (PNOC), Dr. Michelle Monje's studies, and The Cancer Genome Atlas[37–42].

Using the *limma* package in R [43], we conducted differential expression analysis between the H3K27M and nonK27M pHGG cohorts. A total of 1905 genes are differentially expressed between the two tumor types (Supplementary Table 2). Using Gene Set Enrichment Analysis (GSEA) and the Molecular Signatures Database (MSigDB)[44], we found 23 biological signaling pathways with significant enrichment in protein coding genes overexpressed in the H3K27M cohort (Supplementary Table 2). The top 5 most significantly enriched gene pathways included

"Hallmark KRAS Signaling Down" (genes repressed by KRAS activation) and the "Hallmark Epithelial Mesenchymal Transition" (Figure 1A). KRAS pathway enrichment is consistent with a recent study which found RAS signaling to be activated in H3K27M gliomas[45].

Because genes involved in the epithelial-mesenchymal transition (EMT) are regulated by deposition of H3K27me3, an epigenetic transcriptional repressive mark that is lost in H3K27M cells, we were particularly interested in the differential expression of genes involved in the EMT pathway. The Hallmark EMT pathway gene list is limited to 200 genes[46], so to comprehensively characterize expression of EMT-associated genes in H3K27M mutant versus nonK27M tumors, we generated a master list of non-redundant EMT-related genes (n=437) by merging several MSigDB developmental and cellular EMT-related gene sets (Supplementary Table 2). We included only genes from gene sets focused on EMT as a developmental process, and eliminated gene sets that were derived from published studies of EMT in adult carcinomas as per MSigDB[46], because the epithelial nature of those cancers makes those gene sets inapplicable to pediatric gliomas. This list includes genes implicated in both pre- and post-EMT cell states, as well as intermediate EMT cell states and EMT-like processes.

To investigate differential EMT gene expression, we calculated the overlap between the EMT master list and the differentially expressed genes (Supplementary Table 2). We found ~~123~~ 49 differentially expressed genes from the EMT master list, indicating potential differential activity of the EMT pathway in H3K27M mutant gliomas (pvalue$<7.89^{-14}$, hypergeometric test). Of these genes, 26 were more highly expressed in H3K27M tumors, and the remaining 23 were more highly expressed in nonK27M tumors. (Figure 1B). Further investigation via manual inspection revealed that, in general, the EMT-related genes overexpressed in the H3K27M cohort are associated with the transcriptional profile of cells prior to an EMT-like transition. In contrast,

many of the EMT genes underexpressed in H3K27M tumors associated with a post-EMT cell state.

To statistically quantify the association of the 26 EMT-related genes overexpressed in the H3K27M cohort with pre-EMT cell states in the brain, we manually identified 9 gene sets relating to epithelial cells and early brain development (Supplementary Table 2). The H3K27M-high EMT genes had significant enrichment in 8/9 gene sets (pvalue<0.1), In contrast, we calculated the enrichment of 26 randomly selected genes in these 9 gene sets and they were not significant (Supplementary Table 2). The enriched epithelial gene sets include “GO Epithelium Development” (pvalue<3.4^{-12}, hypergeometric test), “GO Epithelial Cell Differentiation”, (pvalue<3.587^{-04}), and “GO Neural Tube Formation” (pvalue<0.001). In the developing brain, some of the cells of the neural tube, a pseudostratified epithelium, undergo an EMT in order to migrate[21]. *RHOB*, which plays a role in epithelial cell maintenance in the neural tube[47], is more highly expressed in H3K27M tumors and belongs to 3/9 epithelial gene sets. Additionally, *SFRP1* and *SFRP2*, which are crucial in neural tube formation[48], are more highly expressed in H3K27M tumors and belong to 6/9 epithelial gene sets.

Importantly, we noted that *SNAI1*, a transcription factor and key regulator of the EMT transcriptional program, is significantly overexpressed in H3K27M tumors (LFC=0.6; Figure 1C). High expression of *SNAI1* is a marker of the beginning of the induction of EMT or EMT-like cellular transitions. If the transition is successful, this is followed by high expression of post-EMT markers *TWIST1[49]*, fibronectin (*FN1*)[50], N-cadherin (*CDH2*)[51] and cadherin-11 (*CDH11*)[52]. Using a Mann-Whitney nonparametric significance test, we found significantly reduced expression of all of these genes in H3K27M tumors (*TWIST1* LFC=-1.2, *FN1* LFC=-0.2, *CDH2* LFC=-0.2, *CDH11* LFC=-0.3; Figure 1C). *TWIST1*, *CDH2* and *CDH11* are also underexpressed in the H3K27M cohort by the *limma* analysis.

Because SNAI1 induces EMT-like processes in the developing brain by directly recruiting PRC2 methyltransferase activity for H3K27-trimethylation, a process blocked by the H3K27M mutation, we hypothesized that the occurrence of the H3K27M mutation may promote tumorigenesis by stalling EMT during early neuroepithelial differentiation. To further investigate this hypothesis, we performed comparative RNA-sequencing expression outlier analysis developed by the Treehouse Childhood Cancer Initiative, which identifies genes with outlier expression in individual samples as compared to a background cohort of highly correlated and disease-matched samples (pan-disease analysis, see Methods) [34]. We identified genes with outlier expression only in nonK27M pHGG samples (but not H3K27M pHGG samples) as compared to a background glioma cohort, and noted that four of the top ten enriched pathways were related to EMT, including “TGF-Beta regulation of the extracellular matrix” (adjusted pvalue 4.01^{-09}) and “Extracellular matrix organization” (adjusted pvalue 2.98^{-05}) (Supplementary Figure 1, Supplemental Table 2).

Finally, because EMT is associated with invasiveness in gliomas, and diffuse midline glioma are by nature more invasive than hemispheric glioma, we performed an additional analysis restricted to diffuse intrinsic pontine glioma (DIPG) to elucidate the role of the H3K27M mutation in the observed EMT-related transcriptional profiles. The goal of this analysis was to remove any potential histological or location signal that may be influencing the EMT-related gene expression. We used 10 H3 wild-type DIPG samples and 47 H3K27M DIPG samples from Treehouse cancer compendium v11.

Limma differential expression analysis revealed 48 genes with higher expression in H3K27M DIPG compared to nonK27M DIPG (Supplementary Table 2). We again computed statistical overlap of these genes with 9 gene sets relating to epithelial cells and early brain development, and found significant overlap with 4 of the 9 gene sets (pvalue<0.1, Supplementary Table 2).

The enrichment of 48 randomly selected genes in these 9 gene sets were not significant (Supplementary Table 2).

Overall, our multiple analyses of the pHGG RNA-seq cohort suggest that H3K27M pHGG tumors are characterized by a transcriptional profile typically expressed by cells before undergoing an EMT-like transitional process, while nonK27M pHGG tumors are characterized by post-EMT gene expression.

Figure 1. The EMT pathway is differentially expressed in H3K27M gliomas as compared to nonK27M gliomas. A) Differential expression analysis of a cohort of H3K27M and nonK27M pHGG revealed significant enrichment of Hallmark Epithelial Mesenchymal Transition in genes overexpressed in H3K27M gliomas. B) Heatmap of differentially expressed EMT genes between H3K27M and nonK27M pHGG. C) *SNAI1* is overexpressed in H3K27M glioma, while *TWIST1*, *FN1*, *CDH2* and *CDH11* are underexpressed in H3K27M glioma as compared to nonK27M gliomas (Mann-Whitney significance test; * pvalue < 0.05, ** pvalue < 0.01, *** pvalue < 0.001).

B. H3K27M-mediated gliomagenesis is associated with pre-EMT cell types.

Consistent with our differential expression analysis, a review of the literature revealed that H3K27M-associated gliomagenesis has been experimentally recapitulated only in cell types which are poised to undergo an EMT differentiation event (Figure 2A). For example, a combination of H3K27M, *p53* loss, and *PDGFRA* constitutive activation in human neural progenitor cells (NPCs) induced low grade gliomas when injected into the pons of neonatal mice[16]. These gliomas expressed markers of pre-EMT neuroepithelial cells. Another study found that H3K27M and *Trp53* loss was sufficient for gliomagenesis in the NPCs of embryonic mice in the forebrain and hindbrain[17]. Strikingly, when introduced post-natally, H3K27M and

p53 loss in pre-EMT NPCs was not sufficient for gliomagenesis, although post-natal induction of H3K27M, *Trp53* loss and *PDGFRA* amplification in pre-EMT NPCs resulted in glioma formation[53,54]. Additionally, no tumorigenesis was observed upon introduction of H3K27M, *p53* loss and *PDGFRA* constitutive activation in mature astrocytes, a post-EMT cell type[16]. These observations indicate that experimental H3K27M-mediated gliomagenesis occurs in a pre-EMT cell type.

Based on our gene expression analysis and review of the literature, we hypothesized that H3K27M gliomas arise in pre-EMT cell types and retain the EMT-related transcriptional profile of the cell type in which the mutation arises. In order to compare the expression of the EMT-related genes of interest between H3K27M tumors and normal developing brain cells, we examined total and single cell RNA-seq data from a human embryonic stem cell-derived cerebral cortex organoid time course experiment (Figure 2B)[35]. These organoid cultures mimic the early weeks of human prenatal cortical development and generate relevant cell types, uniquely allowing us to investigate early time-points in development which are not available in existing human fetal brain datasets. After induction of neural epithelium by week 1, at week 2 radial glia cells and Cajal-Retzius neurons are present in addition to some remaining neuroepithelial cells. By week 5, the organoids contain populations of radial glia, intermediate progenitors and deep-layer neurons.

When we investigated EMT-related gene expression in cerebral organoids during gestational weeks 1-6, we noted the presence of two EMT-related transcriptional transitions (Figure 2A, lower panel). The first transition starts as *SNAI1* expression peaks in neural stem cells (week 1), coincident with low expression of post-EMT markers *TWIST1*, *CDH2, CDH11* and *FN1*. As differentiation from neural epithelial cells to early radial glia occurs, *SNAI1* expression

decreases while post-EMT marker expression increases. In the second transition, as radial glia cells differentiate intermediate progenitor cells, *SNAI1* expression increases once again.

To further characterize the EMT-like transcriptional profiles represented in cerebral organoids, we utilized single cell RNA-seq data from the cerebral organoids at gestational weeks 3 and 6[35]. These sample collection times effectively covered all relevant cell type diversity, as gestation week 3 organoids contain substantial populations of neural epithelial cells, early radial glia cells and Cajal-Retzius neurons, while week 6 organoids are composed of late radial glia cells, intermediate progenitors, and immature neurons. We scored the EMT status of each cell using a gene signature representing EMT completion and a previously published scoring method based on aggregate expression of the gene set as compared to a control gene set (Figure 2C, Supplementary Table 3, see Methods)[18,55–58]. Neural epithelial and early radial glia cells show significantly lower EMT scores than post-EMT intermediate progenitors, late radial glia and neurons (Mann-Whitney test, pvalue<0.0001). This shows that our assay contains distinct populations of pre- and post-EMT cerebral cells, and is consistent with the levels of *SNAI1*, *CDH2*, *CDH11, FN1* and *TWIST1* in the bulk weeks 1-6 organoid data. This dataset enables us to investigate transcriptional similarities between H3K27M-mutant gliomas and normal pre-EMT cell types during neural development.

We then examined the expression of genes overexpressed in H3K27M gliomas in the single cell organoid RNA-seq dataset, to see which normal cell type is most similar to H3K27M glioma cells. Of the 1180 H3K27M-overexpressed genes, 152 genes passed the single cell RNA-seq expression filter (Supplementary Table 3, see Methods). Hierarchical clustering of the expression profiles of these genes in normal cell types during neural development revealed highest expression in pre-EMT neural epithelium and early radial glia (Figure 2D). We then ranked this gene signature based on each gene’s expression in each cell type (see Methods).

We found that this signature is ranked most highly in pre-EMT neural epithelium and in early radial glia (pvalue<0.05, Figure 2E).

Overall, these results suggest that the differential EMT-related gene expression observed in our tumor cohort is consistent with identifiable stages in cyclic EMT-like transcriptional programs in the normal developing brain, and that H3K27M tumor cells resemble normal developing brain cells at a point where they are expressing a pre-EMT transcriptional profile.

Figure 2. H3K27M-specific EMT transcriptional signature is similar to pre-EMT neural stem cell expression in cerebral organoids. A) *In vitro* and *in vivo* experimental H3K27M-associated gliomagenesis occurs exclusively in pre-EMT cell types (upper panel). These cell types are represented in our cerebral organoid assay, and a time course of these organoid cultures represents 2 EMT events in early brain development (lower panel). B) Experimental workflow for total RNA-seq and single cell RNA-seq from a human embryonic stem cell derived cerebral cortex organoid time course experiment. C) Single cells from cerebral organoids were scored for EMT completion. Pre-EMT neural epithelium and early radial glia were least enriched for the EMT score, while post-EMT intermediate progenitors, late radial glia and neurons were the most enriched. D) A signature of genes differentially expressed in H3K27M gliomas and expressed in cerebral organoids shows highest expression in pre-EMT neural epithelium and early radial glia. E) EMT-related genes highly expressed in H3K27M-mutant gliomas are also highly expressed in neural epithelium and early radial glia. (Mann-Whitney significance test; * pvalue < 0.05, ** pvalue < 0.01, **** pvalue < 0.0001)

C. Single-cell profiling of H3K27M gliomas reveals groups of cells with different EMT-related transcriptional profiles.

We utilized recently published single cell RNA-seq data from 6 H3K27M and 2 H3 wild type (H3WT) gliomas to directly investigate the EMT-related transcriptional profiles of single cell populations within each tumor type[18]. One of the H3K27M tumors harbors the mutation in the *HIST1H3B* gene (referenced as H3.1K27M), while the remaining 5 H3K27M tumors harbor the mutation in the *H3F3A* gene (referenced as H3.3K27M).

We performed hierarchical clustering of 3057 tumor cells using 207 genes from the EMT master list which passed expression filters (see Methods, Supplementary Table 4)[59]. Nine EMT-related clusters were discovered and named A-I (Figure 3A, Supplementary Table 4). Cluster gene signatures were identified by assigning each cluster the genes with maximum mean expression in that cell cluster across the dataset (Supplementary Table 4).

We assigned cluster function based on manual review of genes in each signature, and observed several populations of cells whose presence in this dataset has already been noted[18]. Cluster I is composed predominantly of non-malignant immune cells, indicated by comparatively high expression of immune markers such as *CD68[60]*. Cluster H resembles oligodendrocytic cells, with highest expression of *PADI2, PMP22* and *RHOA*, and cluster G resembles oligodendrocyte precursor cells with the highest expression of *PDGFRA[61–64]*. The presence of each of these cell types has already been noted in H3K27M gliomas[18].

However, the remaining clusters are defined by genes associated with EMT. We again scored the EMT status of each cell with a gene signature representing EMT completion (Figure 3A, see Methods)[18,55–58]. Clusters D, E and F scored the lowest overall, while clusters A, B and C scored the highest overall. Cluster relationships are shown with Uniform Manifold Approximation

and Projection (UMAP) in Figure 3B, and expression patterns of selected genes relating to transcriptional stages of EMT-like transitions are shown in the lower panel of Figure 3B. Of the genes identified in the bulk RNA sequencing analysis (Figure 1C), only *FN1*, *CDH2* and *CDH11* were expressed in the glioma single cell RNA-seq data, so we also visualized *VIM* as a post-EMT marker and *SFRP1* as a pre-EMT marker.

In keeping with our previous analysis, we noted that cluster A, which is composed mainly of H3WT glioma cells, strongly resembles post-EMT cells and most highly expresses canonical post-EMT markers including *CDH2*, *CDH6* and *VIM[65,66]*. This is consistent with our observation that nonK27M gliomas transcriptionally resemble a post-EMT state as compared to H3K27M in the bulk RNA-seq pHGG cohort.

Interestingly, within the clusters composed predominantly of H3K27M cells, we observed multiple EMT-related transcriptional profiles. Cluster B, composed of H3K27M cells, had highest expression of post-EMT markers including *CDH11* and *FN1*, potentially indicating a subclonal population of cells which differentiated through alternative means. Thus, we defined Clusters A and B “post-EMT”.

In contrast, H3K27M-expressing clusters E and F cells exhibit comparatively the highest expression of several genes known for their expression in pre-EMT cell types, including *CADM1*, *PTEN, CTNNB1* and *SFRP1[48,67–70]*. Therefore, we defined Clusters E and F “pre-EMT”. In contrast, Cluster C has comparatively the highest expression of only 10 genes and has no clear expression profile of any stage of EMT, so we defined Cluster C “EMT-ambiguous”.

Cluster D was defined “EMT-intermediate”, because it displays high expression of genes normally expressed while the EMT process is taking place, without a clear bias towards

epithelial or mesenchymal gene expression, including *SMAD2* and *VCAN,* which are activated during the EMT process rather than before or after[71,72].

Figure 3. Single cell RNA sequencing of H3K27M and nonK27M gliomas reveals multiple EMT stages within tumors. A) Expression heatmap showing hierarchical clustering of 3,057 cells from 6 H3K27M and 2 nonK27M high-grade gliomas, with a master list of EMT genes. Ten clusters (A-J) were assigned gene signatures based on maximum mean gene expression in each cluster, and clusters were classified based on manual review of each gene signature. Histone H3 mutation status and EMT score are shown at the bottom of the heatmap (ODC=oligodendrocyte, OPC=oligodendrocyte precursor). B) UMAP dimensionality reduction projection of the same expression data as the heatmap and labeled by cluster, Histone H3 mutation status and EMT score. Expression of selected pre-EMT and post-EMT genes shown in the bottom panel.

D. Histone H3.1K27M glioma cells express a different EMT-related transcriptional profile than H3.3K27M glioma cells.

Further examination revealed that cluster D mainly consists of cells from the H3.1K27M mutant tumor. H3.1 and H3.3K27M characterize two functionally different subtypes of H3K27M gliomas; H3.1K27M gliomas are comparatively rare but have a slightly better prognosis[40,73]. Normally, histone H3.3 is preferentially located at active chromatin[74–76]. This leads to distinct patterns of epigenetic reprogramming in each histone variant, where loss of the H3.3K27me3 mark is directly correlated with areas of H3.3 genomic enrichment, while H3.1K27me3 loss is higher at intergenic regions[76,77]. Because the H3K27M mutation is known to induce dose-dependent inhibition of PRC2 methyltransferase, this suggests that the localized distribution of histone H3.3

may result in higher local inhibition of PRC2 and loss of H3K27me3 at H3.3K27M sites[53,76]. Because precise control of gene transcription via active chromatin is necessary for EMT-like developmental cell state transitions, a H3.3K27M mutation would be particularly damaging to proper regulation of these processes. Indeed, functional analysis of enhancer regions in H3.3K27M-expressing NPCs revealed enrichment of regions positively regulating EMT-related genes, indicating that H3.3 active chromatin regions are directly involved in transcriptional control of EMT-related genes[76]. This suggests that EMT-poised H3.3K27M cells will be unable to properly complete the transition due to lack of transcriptional control.

Accordingly, we observed EMT-intermediate or E/M hybrid expression genes in glioma single-cell cluster D, which has a substantial number of H3.1K27M glioma cells. We hypothesized that H3.1K27M cells may be more differentiated than H3.3K27M cells.

In order to investigate this hypothesis further, we subset the single cell glioma RNA-seq data to 2458 cells with H3.1K27M or H3.3K27M mutation and performed Wilcoxon rank-sum test to identify genes overexpressed in each variant group (Supplementary Table 4; Supplementary Figure 2). Consistent with our previous observations, GSEA of Gene Ontology (GO) gene sets (Figure 4B, Supplementary Table 4) revealed enrichment of epithelial gene sets in H3.3K27M compared to H3.1K27M (GO Adhesion pathways, GO Neurogenesis, GO Embryo Development) and mesenchymal gene sets in H3.1K27M compared to H3.3K27M (GO EMT pathway, GO Mesenchymal Cell Differentiation and GO Mesenchyme Development). Additionally, scoring of all cells for EMT completeness shows that H3.1K27M cells score significantly higher overall than H3.3K27M cells, while nonK27M cells score significantly higher than either mutant cell type (Supplementary Figure 3). However, because the H3.1K27M cells come from a single tumor, we performed additional analysis to investigate this observation.

We cultured DIPG primary cell lines isolated in a previous study to investigate the expression of EMT markers in H3.3K27M, H3.1K27M and nonK27M glioma cells[78]. Morphologically, we observed that when cultured in serum-free conditions, the H3.1K27M cell lines preferentially grow attached to the flask (4 of 5 cell lines), while the H3.3K27M cells preferentially grow as neurospheres (8 of 9 cell lines) (Figure 4C). Because differentiation out of the neurosphere state is accompanied by attachment and increased expression of N-cadherin, this morphological trend is consistent with our hypothesis that H3.1K27M cells exist in a more differentiated state than H3.3K27M cells[79].

We analyzed RNA-seq data from 3 DIPG cell lines to compare the expression of EMT genes (SU-DIPG-IV is H3.1K27M mutant; SU-DIPG-VI and JHH-DIPG1 are H3.3K27M mutant). We used 4 replicate samples from each SU-DIPG-IV and SU-DIPG-VI and 3 replicate samples from JHH-DIPG1. Each sample was scored using a gene signature of EMT completion (see Methods), and the H3.1K27M samples scored significantly higher than the H3.3K27M samples (Figure 4D, pvalue<0.05).

We then performed RT-PCR to quantify the expression of *FN1* and *CDH2*, canonical post-EMT genes which were previously identified as differentially expressed by Mann-Whitney test in the bulk glioma RNA sequencing analysis (Figure 4E, full-length gel in Supplementary Figure 4). We attempted to quantify E-cadherin/*CDH1* as it is a canonical pre-EMT marker, but the levels were so low as to be undetectable by RT-PCR in these cell lines (RNA-seq <1.0 log2(TPM+1)). We compared 9 H3.3K27M cell lines (SU-DIPG-VI, XIII, XVII, XIX, 24, 25, 27, 35 and 43) with 5 H3.1K27M cell lines (SU-DIPG-IV, XXI, 33, 36 and 38) and included 5 H3 wild-type lines (SU-DIPG-48, pcGBM2R, KNS42, SJG2 and normal human astrocytes hTERT) and a negative RT-PCR control (NC). Overall, the H3 wild-type and H3.1K27M cell lines appear to more highly express both post-EMT markers, in keeping with the bulk and single-cell RNA-seq analyses.

Our computational and *in vitro* observations are consistent with a recent study indicating that H3.1K27M tumor cells are overall more differentiated than H3.3K27M tumor cells[76]. Our results are also consistent with previous studies on EMT in pediatric gliomas which first found a mesenchymal subtype of DIPG and subsequently discovered that H3.1K27M mutant gliomas express genes associated with a more mesenchymal subtype of glioblastoma[73,80].

Overall, these data suggest that the histone H3K27M mutation is associated with a preferentially early or pre-EMT cell state as compared to nonK27M cells, but that H3.1K27M cells may represent a somewhat later or intermediate-EMT cell state as compared to H3.3K27M cells.

Figure 4. H3.1K27M glioma cells express a different EMT-related transcriptional profile than H3.3K27M glioma cells. A) UMAP dimensionality reduction of 2458 histone mutant glioma single cells. B) Gene set enrichment analysis of genes overexpressed in H3.3K27M versus H3.1K27M (top graph) or H3.1K27M versus H3.3K27M (lower graph) by Wilcoxon rank-sum test using glioma single cell RNA-seq data. C) Representative images of H3.1K27M and H3.3K27M glioma derived cell cultures. Scale bar 400 um. D) Total RNA sequencing datasets from glioma cell lines were scored for EMT completeness (4 samples from SU-DIPG-IV, 4 samples from SU-DIPG-VI and 3 samples from JHH-DIPG1). Scoring is shown in a heatmap and a boxplot. (Mann-Whitney significance test; * pvalue < 0.05) E) RT-PCR of *FN1* and *CDH2* expression in glioma primary cell cultures (all numbered lines are SU-DIPG).

Discussion

H3K27M diffuse midline gliomas are aggressive tumors generally occurring in early childhood in the hindbrain or midline. These tumors have poor prognosis and do not respond to standard chemotherapies for adult gliomas[81]. In contrast to most adult cancers, pediatric cancers,

including pediatric gliomas, are thought to occur due to a developmental stall relating to epigenetic dysregulation of normal cellular differentiation pathways[15,40,82]. H3K27M diffuse midline glioma cells lose EZH2-deposited H3K27me3 epigenetic transcriptional control markers, which are known to have crucial roles in cell differentiation and development in the brain[6]. In particular, normal H3K27me3 deposition controls neural cell differentiation through multiple EMT processes [22]. Research has implicated the EMT in pediatric gliomas[80,83,84], particularly those with a more invasive phenotype. Histone deacetylase inhibitor treatment of *in vitro* pHGG cells reversed mesenchymal phenotypes, in keeping with a model in which the interplay between H3K27 acetylation and methylation controls EMT-related transcriptional states[38]. We hypothesized that loss of H3K27me3 in H3K27M mutant gliomas may lead to a stall in EMT processes in normal brain development.

In this study, we observed that various canonical EMT-inducing genes are significantly overexpressed in H3K27M mutant pHGGs, compared to nonK27M pHGGs, while many canonical mesenchymal markers are underexpressed in H3K27M pHGGs as compared to the nonK27M tumors. In particular, we noted higher expression of the pre-EMT transcription factor *SNAI1* in H3K27M-mutant gliomas. Because *SNAI1* relies on PRC2 and H3K27me3 to facilitate EMT through gene expression regulation, this may indicate an arrest in the EMT process. The existence of a hybrid epithelial/mesenchymal phenotype is well-established: the result of a partial EMT is the expression of both epithelial and mesenchymal genes[85]. Studies have shown that a hybrid E/M phenotype may indicate a worse prognosis than mesenchymal-only states in solid tumors[85–87].

We hypothesized that if H3K27M mutation prevents full EMT, neural stem cells harboring H3K27M may be forced to retain a proliferative, stem cell phenotype, eventually leading to tumorigenic development. Accordingly, we observed from extensive literature review that

experimental induction of H3K27M-associated gliomas has occurred exclusively in pre-EMT cell types, and that two consecutive EMT-like transcriptional transitions occur early in normal brain development.

Single cell RNA-seq from H3K27M and nonK27M tumors confirmed a post-EMT expression signature in the nonK27M cells, and also revealed subsets of H3K27M cells with different EMT-related transcriptional profiles. Specifically, we observed an intermediate EMT signature in the H3.1K27M cells as compared to the H3.3K27M cells. This was also observed in bulk RNA-seq and *in vitro* RT-PCR analysis. We hypothesize that because the H3.1K27M mutation is not concentrated at active chromatin, it has less repressive power as specific developmental processes such as EMT are activated over time. If a subset of H3.1K27M cells are able to differentiate, this may explain why H3.1K27M gliomas have a slightly better prognosis.

To conclude, we mined 3 publicly available RNA-seq datasets from pediatric gliomas and cerebral organoids to generate a hypothesis for the gliomagenesis of H3K27M gliomas. We propose that the H3K27M mutation is tumorigenic when the mutational hit occurs in a cell poised to undergo an EMT-like cell state transition, due to the dependence of EMT-associated transcriptional activity on the correct timing of the H3K27me3 mark (Figure 5). More work is needed to characterize the observed difference in the EMT-associated transcriptional profiles between the H3.1 and H3.3K27M variants. Additionally, a limitation of our study is that it is difficult to isolate the role of the H3K27M mutation from other factors such as histology and tumor location. Future studies will focus on EMT-related transcriptional programs in cellular models with inducible H3K27M expression to further characterize the molecular interplay between the H3K27M mutation and EMT in developing brain cells.

Taken together, our results hold important implications for better understanding the developmental origin and timing of these aggressive and untreatable cancers. Further, the

presence of an epigenetically-driven differentiation stall may imply that a pharmacological methylation agent or a pro-differentiation therapy may aid in future treatment of H3K27M mutant tumors[88].

Figure 5. Proposed model for EMT stall in H3K27M cells. We propose that H3K27M cells retain high levels of SNAI1 expression but remain stalled in a pre-EMT state due to inability of PCR2 to tri-methylate H3K27.

Potential Implications

Our study holds implications for other diseases, because H3K27M mutation is not exclusive to diffuse midline gliomas. It can also be found in a fraction of pediatric ependymomas and medulloblastomas[89]. Interestingly, ependymomas located in the posterior fossa typically do not harbor the H3K27M mutation, but exhibit the K27M-associated H3K27 hypomethylation phenotype. Thus, the proposed differentiation stall and an associated EMT transcriptional signature as a result of H3K27me3 loss may also apply to these cancers. Beyond the SNAI1-H3K27me3 axis, EMT is also regulated by other epigenetic marks[90]. Given the epigenetically dysfunctional nature of many pediatric cancers[15], EMT arrest could conceivably play a role in the oncogenesis of these tumors as well.

Methods

Glioma bulk RNA sequencing data

Gene expression data from 78 pediatric high grade glioma samples were downloaded from the Treehouse Childhood Cancer Initiative public compendium v8 (Tumor Compendium v8 Public)[36]. All samples in the compendium have been uniformly processed using the UC Santa

Cruz TOIL RNA-seq pipeline (v3.3.4)[91]. This dataset (n=58581 genes) is in transcripts per million (TPM) and normalized by log2(TPM+1). We divided the dataset into 33 H3K27M mutant samples and 45 nonK27M samples, and performed differential expression analysis of all genes between the two groups using R library *limma* v3.34.9 in R v3.3.4. We performed gene set enrichment analysis (GSEA) of the resulting 1905 differentially expressed genes (pvalue<0.1) with Molecular Signatures Database (MSigDB) v7.0 on the GSEA/MSigDB web site v6.4 (Supplementary Table 2). Since the epithelial-mesenchymal transition (EMT) pathway was in the top 5 most significantly enriched pathways in H3K27M over expressed genes, we created a non-redundant master list of EMT genes (n=1226) by merging 15 EMT related MSigDB pathways and by identifying EMT-related genes through manual literature curation (Supplementary Table 2).

We performed pan-disease outlier analysis on all the pHGG samples using Treehouse CARE (see Availability of source code and requirements section) against the Treehouse Cancer Compendium v10. Pan-disease outlier analysis identifies genes with outlier expression in each sample of interest as compared to a background cohort of tumors identified as most similar (in this analysis, the background cohort was 37 pediatric gliomas, 19 young adult gliomas, 18 pediatric glioblastomas and 4 young adult glioblastomas)[34]. We identified a list of genes with outlier expression in the nonK27M pHGG samples that did not also have outlier expression in the H3K27M pHGG samples, and performed gene set enrichment analysis using Enrichr in the GSEApy package (gseapy-v0.9.17)[92] against BioPlanet_2019 library with p-value cutoff 0.05 (outlier genes and enriched pathways in Supplementary Table 2). We used the EnrichmentMap app in Cytoscape to visualize functionally similar clusters of enriched pathways[93].

Cerebral organoid RNA sequencing data (bulk and single cell)

Gene expression data (TPM) from 6 weekly timepoints of human cerebral organoid growth were downloaded from accession GSE106245[35]. Organoid weeks 0-5 were converted to gestational weeks 1-6 and duplicate gene measurements were averaged. For Figure 2A, expression of each gene was normalized between 0-1. Single cell RNA sequencing data from weeks 2 and 5 (gestational weeks 3 and 6) cerebral organoids were downloaded from accession GSE106245[35]. Expression data were filtered to remove genes with expression in fewer than 10% of cells. Cell types were assigned using a list of marker genes (Supplementary Table 3).

Glioma single cell RNA sequencing data

Smart-seq2 RSEM TPM single cell RNA sequencing data from 3,057 glioma cells were downloaded from accession GSE102130[18]. Data were log2-normalized and filtered to remove genes with expression in fewer than 20% of cells. The cells per tumor remaining after filtering are as follows: MUV1: 146, MUV5: 708, MUV10: 286, BCH836: 527, BCH869: 492, BCH1126: 299, MGH66: 442, MGH101: 92, MGH104: 65. Hierarchical clustering of all cells was performed using the Python *scipy.cluster.hierarchy* function (scipy v1.4.1) after subsetting to a non-redundant master list of EMT genes (n=1226, Supplementary Table 2). Of these genes, 629 passed the expression filter and were included in the hierarchical clustering. The clustering results were plotted using the *scipy.cluster.hierarchy.dendrogram* function with threshold set to 3.5. Gene signatures for each cluster were assigned by identifying the cluster in which each gene has maximum mean expression, and assigning that gene to that cluster. For UMAP visualizations, Leiden clustering was performed on the single cell data using the *scanpy.tl.leiden*

function (scanpy v1.4.5.post1) with resolution set to 0.5 and top 10 principle components used as input.

DIPG Cell Lines

The patient-derived DIPG cell lines (SU-DIPG-IV, SU-DIPG-VI, SU-DIPG-XIII, SU-DIPG-XVII, SU-DIPG-XIX, SU-DIPG-XXI, SU-DIPG-24, SU-DIPG-25, SU-DIPG-27, SU-DIPG-33, SU-DIPG-35, SU-DIPG-36, SU-DIPG-38, SU-DIPG-48) were kindly provided by Dr. Michelle Monje (Stanford University School of Medicine, Stanford CA)[38]. SU-DIPG-IV, SU-DIPG-XXI, SU-DIPG-33, SU-DIPG-36, and SU-DIPG-38 cells harbor a H3.1K27M mutation while SU-DIPG-VI, SU-DIPG-XIII, SU-DIPG-XVII, SU-DIPG-XIX, SU-DIPG-24, SU-DIPG-25, SU-DIPG-27, SU-DIPG-35, SU-DIPG-43 cells harbor a H3.3K27M mutation. SU-DIPG-48 and Glioblastoma cell line SU-pcGBM-2 are H3WT. Glioblastoma H3WT cell lines; KNS-42 (RRID:CVCL_0378), SJ-GBM2 (RRID:CVCL_M141), and one normal astrocyte cell line NHA hTERT were kindly provided by Prof. Sameer Agnihotri (UPMC Children's Hospital of Pittsburgh, Pittsburgh PA). The Universal Mycoplasma Detection Kit (AACC) was used for testing SU-DIPG-XIII, XVII, XIX, and VI latest on January 10, 2020. All cells were cultured in tumor stem medium containing 50X B-27 Supplement Minus Vitamin A (Invitrogen), H-EGF at 20ng/mL (Shenandoah Biotechnology), H-FGF-basic-154 at 20ng/mL (Shenandoah Biotechnology), H-PDGF-AA at 10ng/mL (Shenandoah Biotechnology), H-PDGF-BB at 10ng/mL (Shenandoah Biotechnology), and 0.2% Heparin Solution at 2ug/mL (STEMCELL Technologies). All experiments used cells collected within 5 passages after thawing. The cells were passaged by the treatment of TrypLE (Gibco) and DNAse I (Worthington) rocking at 37°C for 5-15 minutes then HBSS (Corning) was added to deactivate TrypLE. The cells were transferred to new Nunc EasYFlask Cell Culture Flasks (ThermoFisher Scientific) and grown in

tumor stem medium as previously described. The bulk RNA sequencing data from lines SU-DIPG-VI, SU-DIPG-IV and JHH-DIPG1 were obtained with permission from Dr. Michelle Monje from dbGap accession phs000900.v1.p1.

RNA Extraction and RT-PCR

Total RNA was extracted from cell pellets using the Quick-RNA Miniprep Kit (Zymo Research). cDNA was synthesized from 1 ug of total RNA using Oligo(dT)20 primers and the SuperScript III First Strand Synthesis System (Invitrogen). PCR was performed using KAPA HiFi HotStart ReadyMixPCR Kit (KAPA Biosystems), 50 ng of template DNA and the appropriate primers and 27 PCR cycles and annealing temperature 64C. *CDH2* primer sequences: forward: ggcttaatggtgattttgctcag reverse: tccataccacaaacatcagcac. *FN1* primer sequences: forward: cttgaaccaacctacggatgac reverse: tcccatcatcataacacgttgc. Primer oligos were purchased from Integrated DNA Technologies.

Data Analysis

All statistical comparisons are performed with a two-sided Mann-Whitney test, with measurements taken from distinct samples without assumption of normality, and Benjamini Hochberg multiple testing correction was applied. Single cell and bulk tumor samples were scored for EMT activity using a manually curated set of mesenchymal genes and a previously published scoring method based on aggregate expression of the gene set as compared to a control gene set (Supplementary Table 3)[18,56,94].

Availability of source code and requirements

Code for figures and data analysis: github.com/lauren-sanders/EMT-paper/ Apache-2.0 License

Code for outlier analysis: github.com/UCSC-Treehouse/CARE/ Apache-2.0 License

Operating system: Platform independent

Programming languages: Python, R

Availability of supporting data and materials

All data used in the manuscript is available at the following websites or accession numbers: Publicly available: 1) bulk glioma RNA-seq: treehousegenomics.soe.ucsc.edu/public-data, 2) cerebral organoid RNA-seq: GSE106245, 3) glioma single-cell RNAseq: GSE102130. Data available with permission for the glioma cell line RNA-seq data dbGap phs000900.v1.p1. All supporting data and materials are available in the GigaScience GigaDB database[95].

Declarations

List of abbreviations

DIPG: diffuse intrinsic pontine glioma; E/M: epithelial/mesenchymal; EMT: epithelial-mesenchymal transition; GO: gene ontology; GSEA: gene set enrichment analysis; H3WT: histone 3 wild-type; MSigDB: molecular signatures database; NPC: neural progenitor cells; ODC: oligodendrocyte cells; OPC: oligodendrocyte precursor cells; pHGG: pediatric high-grade gliomas; PNOC: Pacific Pediatric Neuro-Oncology Consortium; TPM: transcripts per million; UMAP: Uniform Manifold Approximation and Projection; WHO: World Health Organization.

Ethics Statement

The protocols for the PNOC-003 trial, Dr. Michelle Monje's studies, Dr. Mariella Filbin's studies, The Cancer Genome Atlas, the Children's Brain Tumor Tissue Consortium, the International Cancer Genome Consortium, and the University of Michigan Clinical Sequencing Exploratory Research have been previously described[18,37–42]. The UCSC Treehouse Childhood Cancer Initiative protocol was approved by the UCSC Institutional Review Board (No. HS2648)[34].

Funding and Acknowledgements

This study was funded by American Association for Cancer Research NextGen Grant for Transformative Cancer Research Award (OMV), St Baldrick's Foundation Consortium Award and Emily Beazley Kures for Kids Fund Hero Award (DH, OMV, SS), Alex's Lemonade Stand Foundation for Childhood Cancer Research, Unravel Pediatric Cancer, Team G Childhood Cancer Foundation, and Live for Others Foundation, The Schmidt Futures Foundation (DH), CIRM Shared Stem Cell Facilities (CL1-00506) award to UCSC. AC is supported by the T32GM133391 Training Program in Molecular, Cell, and Developmental Biology. DH is a Howard Hughes Medical Institute Investigator. OMV holds the Colligan Presidential Chair in Pediatric Genomics. We gratefully acknowledge Dr. Michelle Monje and Prof. Sameer Agnihotri who provided cell lines used in this study.

Author Contributions

Analysis and manuscript authorship: LMS and AC

Single cell organoid cell type gene ranking: LS

Experimental work: AC, AB, MC

Treehouse cancer compendium and manuscript review: HCB, ETK, JP, KL, AGL and IB

Funding, scientific oversight and manuscript review: DH, SRS and OMV

Competing Interests

The authors declare no potential conflicts of interest.

References

1. Juratli TA, Qin N, Cahill DP, Filbin MG. Molecular pathogenesis and therapeutic implications in pediatric high-grade gliomas. Pharmacol Ther. 2018;182: 70–79.
2. Chan K-M, Fang D, Gan H, Hashizume R, Yu C, Schroeder M, et al. The histone H3.3K27M mutation in pediatric glioma reprograms H3K27 methylation and gene expression. Genes Dev. 2013;27: 985–990.
3. Johung TB, Monje M. Diffuse Intrinsic Pontine Glioma: New Pathophysiological Insights and Emerging Therapeutic Targets. Curr Neuropharmacol. 2017;15: 88–97.
4. Jones C, Baker SJ. Unique genetic and epigenetic mechanisms driving paediatric diffuse high-grade glioma. Nat Rev Cancer. 2014;14. doi:10.1038/nrc3811
5. Louis DN, Perry A, Reifenberger G, von Deimling A, Figarella-Branger D, Cavenee WK, et al. The 2016 World Health Organization Classification of Tumors of the Central Nervous System: a summary. Acta Neuropathol. 2016;131: 803–820.
6. de Vries NA, Hulsman D, Akhtar W, de Jong J, Miles DC, Blom M, et al. Prolonged Ezh2 Depletion in Glioblastoma Causes a Robust Switch in Cell Fate Resulting in Tumor Progression. Cell Rep. 2015;10: 383–397.
7. Mohammad F, Weissmann S, Leblanc B, Pandey DP, Højfeldt JW, Comet I, et al. EZH2 is a potential therapeutic target for H3K27M-mutant pediatric gliomas. Nat Med. 2017;23: 483–492.
8. Margueron R, Reinberg D. The Polycomb complex PRC2 and its mark in life. Nature. 2011;469: 343–349.
9. Mohn F, Weber M, Rebhan M, Roloff TC, Richter J, Stadler MB, et al. Lineage-specific polycomb targets and de novo DNA methylation define restriction and potential of neuronal progenitors. Mol Cell. 2008;30: 755–766.
10. Roidl D, Hacker C. Histone methylation during neural development. Cell Tissue Res. 2014;356: 539–552.
11. Sher F, Boddeke E, Olah M, Copray S. Dynamic changes in Ezh2 gene occupancy underlie

its involvement in neural stem cell self-renewal and differentiation towards oligodendrocytes. PLoS One. 2012;7: e40399.

12. Sher F, Rössler R, Brouwer N, Balasubramaniyan V, Boddeke E, Copray S. Differentiation of neural stem cells into oligodendrocytes: involvement of the polycomb group protein Ezh2. Stem Cells. 2008;26: 2875–2883.
13. Akizu N, Martínez-Balbás MA. EZH2 orchestrates apicobasal polarity and neuroepithelial cell renewal. Neurogenesis (Austin). 2016;3: e1250034.
14. Zemke M, Draganova K, Klug A, Schöler A, Zurkirchen L, Gay MH-P, et al. Loss of Ezh2 promotes a midbrain-to-forebrain identity switch by direct gene derepression and Wnt-dependent regulation. BMC Biol. 2015;13: 103.
15. Filbin M, Monje M. Developmental origins and emerging therapeutic opportunities for childhood cancer. Nat Med. 2019;25: 367–376.
16. Funato K, Major T, Lewis PW, Allis CD, Tabar V. Use of human embryonic stem cells to model pediatric gliomas with H3.3K27M histone mutation. Science. 2014;346: 1529–1533.
17. Pathania M, De Jay N, Maestro N, Harutyunyan AS, Nitarska J, Pahlavan P, et al. H3.3K27M Cooperates with Trp53 Loss and PDGFRA Gain in Mouse Embryonic Neural Progenitor Cells to Induce Invasive High-Grade Gliomas. Cancer Cell. 2017;32: 684–700.e9.
18. Filbin MG, Tirosh I, Hovestadt V, Shaw ML, Escalante LE, Mathewson ND, et al. Developmental and oncogenic programs in H3K27M gliomas dissected by single-cell RNA-seq. Science. 2018;360: 331–335.
19. Viebahn C. Epithelio-Mesenchymal Transformation during Formation of the Mesoderm in the Mammalian Embryo. Acta Anal. 1995. Available: https://www.karger.com/Article/PDF/147753
20. Duband J-L. Diversity in the molecular and cellular strategies of epithelium-to-mesenchyme transitions: Insights from the neural crest. Cell Adh Migr. 2010;4: 458–482.
21. Kalcheim C. Epithelial-Mesenchymal Transitions during Neural Crest and Somite Development. J Clin Med Res. 2015;5. doi:10.3390/jcm5010001
22. Zou S, Zhang D, Xu Z, Wen X, Zhang Y. JMJD3 promotes the epithelial-mesenchymal transition and migration of glioma cells via the CXCL12/CXCR4 axis. Oncol Lett. 2019;18: 5930–5940.
23. Bolós V, Peinado H, Pérez-Moreno MA, Fraga MF, Esteller M, Cano A. The transcription factor Slug represses E-cadherin expression and induces epithelial to mesenchymal transitions: a comparison with Snail and E47 repressors. J Cell Sci. 2003;116: 499–511.
24. Cano A, Pérez-Moreno MA, Rodrigo I, Locascio A, Blanco MJ, del Barrio MG, et al. The transcription factor snail controls epithelial-mesenchymal transitions by repressing E-cadherin expression. Nat Cell Biol. 2000;2: 76–83.

25. Lin Y, Dong C, Zhou BP. Epigenetic regulation of EMT: the Snail story. Curr Pharm Des. 2014;20: 1698–1705.

26. Galvagni F, Lentucci C, Neri F, Dettori D, De Clemente C, Orlandini M, et al. Snai1 promotes ESC exit from the pluripotency by direct repression of self-renewal genes. Stem Cells. 2015;33: 742–750.

27. Murray SA, Gridley T. Snail family genes are required for left-right asymmetry determination, but not neural crest formation, in mice. Proc Natl Acad Sci U S A. 2006;103: 10300–10304.

28. Carver EA, Jiang R, Lan Y, Oram KF, Gridley T. The mouse snail gene encodes a key regulator of the epithelial-mesenchymal transition. Mol Cell Biol. 2001;21: 8184–8188.

29. Motta FJN, Valera ET, Lucio-Eterovic AKB, Queiroz RGP, Neder L, Scrideli CA, et al. Differential expression of E-cadherin gene in human neuroepithelial tumors. Genet Mol Res. 2008;7: 295–304.

30. Howng S-L, Wu C-H, Cheng T-S, Sy W-D, Lin P-CK, Wang C, et al. Differential expression of Wnt genes, beta-catenin and E-cadherin in human brain tumors. Cancer Lett. 2002;183: 95–101.

31. Itoh Y, Moriyama Y, Hasegawa T, Endo TA, Toyoda T, Gotoh Y. Scratch regulates neuronal migration onset via an epithelial-mesenchymal transition-like mechanism. Nat Neurosci. 2013;16: 416–425.

32. Ohayon D, Garcès A, Joly W, Soukkarieh C, Takagi T, Sabourin J-C, et al. Onset of Spinal Cord Astrocyte Precursor Emigration from the Ventricular Zone Involves the Zeb1 Transcription Factor. Cell Rep. 2016;17: 1473–1481.

33. Hirabayashi Y, Suzki N, Tsuboi M, Endo TA, Toyoda T, Shinga J, et al. Polycomb limits the neurogenic competence of neural precursor cells to promote astrogenic fate transition. Neuron. 2009;63: 600–613.

34. Vaske OM, Bjork I, Salama SR, Beale H, Tayi Shah A, Sanders L, et al. Comparative Tumor RNA Sequencing Analysis for Difficult-to-Treat Pediatric and Young Adult Patients With Cancer. JAMA Netw Open. 2019;2: e1913968.

35. Field AR, Jacobs FMJ, Fiddes IT, Phillips APR, Reyes-Ortiz AM, LaMontagne E, et al. Structurally Conserved Primate LncRNAs Are Transiently Expressed during Human Cortical Differentiation and Influence Cell-Type-Specific Genes. Stem Cell Reports. 2019;12: 245–257.

36. Treehouse Public Data. [cited 21 Apr 2020]. Available: https://treehousegenomics.soe.ucsc.edu/public-data/

37. Mueller S, Jain P, Liang WS, Kilburn L, Kline C, Gupta N, et al. A pilot precision medicine trial for children with diffuse intrinsic pontine glioma - PNOC003: a report from the Pacific Pediatric Neuro-Oncology Consortium. Int J Cancer. 2019. doi:10.1002/ijc.32258

38. Grasso CS, Tang Y, Truffaux N, Berlow NE, Liu L, Debily M-A, et al. Functionally defined therapeutic targets in diffuse intrinsic pontine glioma. Nat Med. 2015;21: 555–559.

39. Ceccarelli M, Barthel FP, Malta TM, Sabedot TS, Salama SR, Murray BA, et al. Molecular Profiling Reveals Biologically Discrete Subsets and Pathways of Progression in Diffuse Glioma. Cell. 2016;164: 550–563.

40. Mackay A, Burford A, Carvalho D, Izquierdo E, Fazal-Salom J, Taylor KR, et al. Integrated Molecular Meta-Analysis of 1,000 Pediatric High-Grade and Diffuse Intrinsic Pontine Glioma. Cancer Cell. 2017;32: 520–537.e5.

41. Robinson DR, Wu Y-M, Lonigro RJ, Vats P, Cobain E, Everett J, et al. Integrative clinical genomics of metastatic cancer. Nature. 2017;548: 297–303.

42. Sturm D, Orr BA, Toprak UH, Hovestadt V, Jones DTW, Capper D, et al. New Brain Tumor Entities Emerge from Molecular Classification of CNS-PNETs. Cell. 2016;164: 1060–1072.

43. Ritchie ME, Phipson B, Wu D, Hu Y, Law CW, Shi W, et al. limma powers differential expression analyses for RNA-sequencing and microarray studies. Nucleic Acids Res. 2015;43: e47.

44. Subramanian A, Tamayo P, Mootha VK, Mukherjee S, Ebert BL, Gillette MA, et al. Gene set enrichment analysis: a knowledge-based approach for interpreting genome-wide expression profiles. Proc Natl Acad Sci U S A. 2005;102: 15545–15550.

45. Koncar RF, Dey BR, Stanton A-CJ, Agrawal N, Wassell ML, McCarl LH, et al. Identification of Novel RAS Signaling Therapeutic Vulnerabilities in Diffuse Intrinsic Pontine Gliomas. Cancer Res. 2019;79: 4026–4041.

46. Liberzon A, Birger C, Thorvaldsdóttir H, Ghandi M, Mesirov JP, Tamayo P. The Molecular Signatures Database (MSigDB) hallmark gene set collection. Cell Syst. 2015;1: 417–425.

47. Kerosuo L, Bronner-Fraser M. What is bad in cancer is good in the embryo: importance of EMT in neural crest development. Semin Cell Dev Biol. 2012;23: 320–332.

48. Chung M-T, Lai H-C, Sytwu H-K, Yan M-D, Shih Y-L, Chang C-C, et al. SFRP1 and SFRP2 suppress the transformation and invasion abilities of cervical cancer cells through Wnt signal pathway. Gynecol Oncol. 2009;112: 646–653.

49. Tran DD, Corsa CAS, Biswas H, Aft RL, Longmore GD. Temporal and spatial cooperation of Snail1 and Twist1 during epithelial-mesenchymal transition predicts for human breast cancer recurrence. Mol Cancer Res. 2011;9: 1644–1657.

50. Stanisavljevic J, Porta-de-la-Riva M, Batlle R, de Herreros AG, Baulida J. The p65 subunit of NF-κB and PARP1 assist Snail1 in activating fibronectin transcription. J Cell Sci. 2011;124: 4161–4171.

51. Javaid S, Zhang J, Anderssen E, Black JC, Wittner BS, Tajima K, et al. Dynamic chromatin modification sustains epithelial-mesenchymal transition following inducible expression of Snail-1. Cell Rep. 2013;5: 1679–1689.

52. Tanaka S, Kobayashi W, Haraguchi M, Ishihata K, Nakamura N, Ozawa M. Snail1 expression in human colon cancer DLD-1 cells confers invasive properties without N-cadherin expression. Biochem Biophys Rep. 2016;8: 120–126.

53. Lewis PW, Müller MM, Koletsky MS, Cordero F, Lin S, Banaszynski LA, et al. Inhibition of PRC2 activity by a gain-of-function H3 mutation found in pediatric glioblastoma. Science. 2013;340: 857–861.

54. Larson JD, Kasper LH, Paugh BS, Jin H, Wu G, Kwon C-H, et al. Histone H3.3 K27M Accelerates Spontaneous Brainstem Glioma and Drives Restricted Changes in Bivalent Gene Expression. Cancer Cell. 2019;35: 140–155.e7.

55. Tirosh I, Venteicher AS, Hebert C, Escalante LE, Patel AP, Yizhak K, et al. Single-cell RNA-seq supports a developmental hierarchy in human oligodendroglioma. Nature. 2016;539: 309–313.

56. Neftel C, Laffy J, Filbin MG, Hara T, Shore ME, Rahme GJ, et al. An Integrative Model of Cellular States, Plasticity, and Genetics for Glioblastoma. Cell. 2019. doi:10.1016/j.cell.2019.06.024

57. Tan TZ, Miow QH, Miki Y, Noda T, Mori S, Huang RY-J, et al. Epithelial-mesenchymal transition spectrum quantification and its efficacy in deciphering survival and drug responses of cancer patients. EMBO Mol Med. 2014;6: 1279–1293.

58. Mak MP, Tong P, Diao L, Cardnell RJ, Gibbons DL, William WN, et al. A Patient-Derived, Pan-Cancer EMT Signature Identifies Global Molecular Alterations and Immune Target Enrichment Following Epithelial-to-Mesenchymal Transition. Clin Cancer Res. 2016;22: 609–620.

59. Virtanen P, Gommers R, Oliphant TE, Haberland M, Reddy T, Cournapeau D, et al. SciPy 1.0: fundamental algorithms for scientific computing in Python. Nat Methods. 2020;17: 261–272.

60. Holness CL, Simmons DL. Molecular cloning of CD68, a human macrophage marker related to lysosomal glycoproteins. Blood. 1993;81: 1607–1613.

61. Richardson WD, Pringle N, Mosley MJ, Westermark B, Dubois-Dalcq M. A role for platelet-derived growth factor in normal gliogenesis in the central nervous system. Cell. 1988;53: 309–319.

62. Li J, Parker B, Martyn C, Natarajan C, Guo J. The PMP22 gene and its related diseases. Mol Neurobiol. 2013;47: 673–698.

63. Ackerman SD, Garcia C, Piao X, Gutmann DH, Monk KR. The adhesion GPCR Gpr56 regulates oligodendrocyte development via interactions with Gα12/13 and RhoA. Nat Commun. 2015;6: 6122.

64. Falcão AM, Meijer M, Scaglione A, Rinwa P, Agirre E, Liang J, et al. PAD2-Mediated Citrullination Contributes to Efficient Oligodendrocyte Differentiation and Myelination. Cell

Rep. 2019;27: 1090–1102.e10.

65. Zeisberg M, Neilson EG. Biomarkers for epithelial-mesenchymal transitions. J Clin Invest. 2009;119: 1429–1437.

66. Sancisi V, Gandolfi G, Ragazzi M, Nicoli D, Tamagnini I, Piana S, et al. Cadherin 6 is a new RUNX2 target in TGF-β signalling pathway. PLoS One. 2013;8: e75489.

67. Vallath S, Sage EK, Kolluri KK, Lourenco SN, Teixeira VS, Chimalapati S, et al. CADM1 inhibits squamous cell carcinoma progression by reducing STAT3 activity. Sci Rep. 2016;6: 24006.

68. Sakurai-Yageta M, Masuda M, Tsuboi Y, Ito A, Murakami Y. Tumor suppressor CADM1 is involved in epithelial cell structure. Biochem Biophys Res Commun. 2009;390: 977–982.

69. Kim J, Kang HS, Lee Y-J, Lee H-J, Yun J, Shin JH, et al. EGR1-dependent PTEN upregulation by 2-benzoyloxycinnamaldehyde attenuates cell invasion and EMT in colon cancer. Cancer Lett. 2014;349: 35–44.

70. Sun Y, Shen S, Liu X, Tang H, Wang Z, Yu Z, et al. MiR-429 inhibits cells growth and invasion and regulates EMT-related marker genes by targeting Onecut2 in colorectal carcinoma. Mol Cell Biochem. 2014;390: 19–30.

71. Xu J, Lamouille S, Derynck R. TGF-beta-induced epithelial to mesenchymal transition. Cell Res. 2009;19: 156–172.

72. Lv Q-L, Huang Y-T, Wang G-H, Liu Y-L, Huang J, Qu Q, et al. Overexpression of RACK1 Promotes Metastasis by Enhancing Epithelial-Mesenchymal Transition and Predicts Poor Prognosis in Human Glioma. Int J Environ Res Public Health. 2016;13. doi:10.3390/ijerph13101021

73. Castel D, Philippe C, Calmon R, Le Dret L, Truffaux N, Boddaert N, et al. Histone H3F3A and HIST1H3B K27M mutations define two subgroups of diffuse intrinsic pontine gliomas with different prognosis and phenotypes. Acta Neuropathol. 2015;130: 815–827.

74. Szenker E, Ray-Gallet D, Almouzni G. The double face of the histone variant H3.3. Cell Res. 2011;21: 421–434.

75. Goldberg AD, Banaszynski LA, Noh K-M, Lewis PW, Elsaesser SJ, Stadler S, et al. Distinct factors control histone variant H3.3 localization at specific genomic regions. Cell. 2010;140: 678–691.

76. Nagaraja S, Quezada MA, Gillespie SM, Arzt M, Lennon JJ, Woo PJ, et al. Histone Variant and Cell Context Determine H3K27M Reprogramming of the Enhancer Landscape and Oncogenic State. Mol Cell. 2019. doi:10.1016/j.molcel.2019.08.030

77. Castel D, Philippe C, Kergrohen T, Sill M, Merlevede J, Barret E, et al. Transcriptomic and epigenetic profiling of “diffuse midline gliomas, H3 K27M-mutant” discriminate two subgroups based on the type of histone H3 mutated and not supratentorial or infratentorial location. Acta Neuropathol Commun. 2018;6: 117.

78. Lin GL, Monje M. A Protocol for Rapid Post-mortem Cell Culture of Diffuse Intrinsic Pontine Glioma (DIPG). J Vis Exp. 2017. doi:10.3791/55360

79. Kim MY, Kaduwal S, Yang DH, Choi KY. Bone morphogenetic protein 4 stimulates attachment of neurospheres and astrogenesis of neural stem cells in neurospheres via phosphatidylinositol 3 kinase-mediated upregulation of N-cadherin. Neuroscience. 2010;170: 8–15.

80. Puget S, Philippe C, Bax DA, Job B, Varlet P, Junier M-P, et al. Mesenchymal transition and PDGFRA amplification/mutation are key distinct oncogenic events in pediatric diffuse intrinsic pontine gliomas. PLoS One. 2012;7: e30313.

81. Jones C, Karajannis MA, Jones DTW, Kieran MW, Monje M, Baker SJ, et al. Pediatric high-grade glioma: biologically and clinically in need of new thinking. Neuro Oncol. 2017;19: 153–161.

82. Hargrave D, Bartels U, Bouffet E. Diffuse brainstem glioma in children: critical review of clinical trials. Lancet Oncol. 2006;7: 241–248.

83. Meel MH, Schaper SA, Kaspers GJL, Hulleman E. Signaling pathways and mesenchymal transition in pediatric high-grade glioma. Cell Mol Life Sci. 2018;75: 871–887.

84. Tam WL, Weinberg RA. The epigenetics of epithelial-mesenchymal plasticity in cancer. Nat Med. 2013;19: 1438–1449.

85. Christiansen JJ, Rajasekaran AK. Reassessing epithelial to mesenchymal transition as a prerequisite for carcinoma invasion and metastasis. Cancer Res. 2006;66: 8319–8326.

86. Grosse-Wilde A, Fouquier d'Hérouël A, McIntosh E, Ertaylan G, Skupin A, Kuestner RE, et al. Stemness of the hybrid Epithelial/Mesenchymal State in Breast Cancer and Its Association with Poor Survival. PLoS One. 2015;10: e0126522.

87. Jolly MK, Mani SA, Levine H. Hybrid epithelial/mesenchymal phenotype(s): The "fittest" for metastasis? Biochim Biophys Acta Rev Cancer. 2018;1870: 151–157.

88. Pan M-R, Hsu M-C, Chen L-T, Hung W-C. Orchestration of H3K27 methylation: mechanisms and therapeutic implication. Cell Mol Life Sci. 2018;75: 209–223.

89. Gröbner SN, Worst BC, Weischenfeldt J, Buchhalter I, Kleinheinz K, Rudneva VA, et al. The landscape of genomic alterations across childhood cancers. Nature. 2018;555: 321–327.

90. Sun L, Fang J. Epigenetic regulation of epithelial-mesenchymal transition. Cell Mol Life Sci. 2016;73: 4493–4515.

91. Vivian J, Rao AA, Nothaft FA, Ketchum C, Armstrong J, Novak A, et al. Toil enables reproducible, open source, big biomedical data analyses. Nat Biotechnol. 2017;35: 314–316.

92. Fang Z. GSEApy.

93. Merico D, Isserlin R, Stueker O, Emili A, Bader GD. Enrichment map: a network-based method for gene-set enrichment visualization and interpretation. PLoS One. 2010;5: e13984.

94. Tirosh I, Izar B, Prakadan SM, Wadsworth MH 2nd, Treacy D, Trombetta JJ, et al. Dissecting the multicellular ecosystem of metastatic melanoma by single-cell RNA-seq. Science. 2016;352: 189–196.

95. Sanders LM, Cheney A, Seninge L, van den Bout A, Chen M, Beale HC, et al. Supporting data for “Identification of a differentiation stall in epithelial mesenchymal transition in histone H3 mutant diffuse midline glioma” GigaScience Database 2020. doi:10.5524/100818.

Figure 1

Click here to access/download;Figure;Figure1_Revision1.pdf

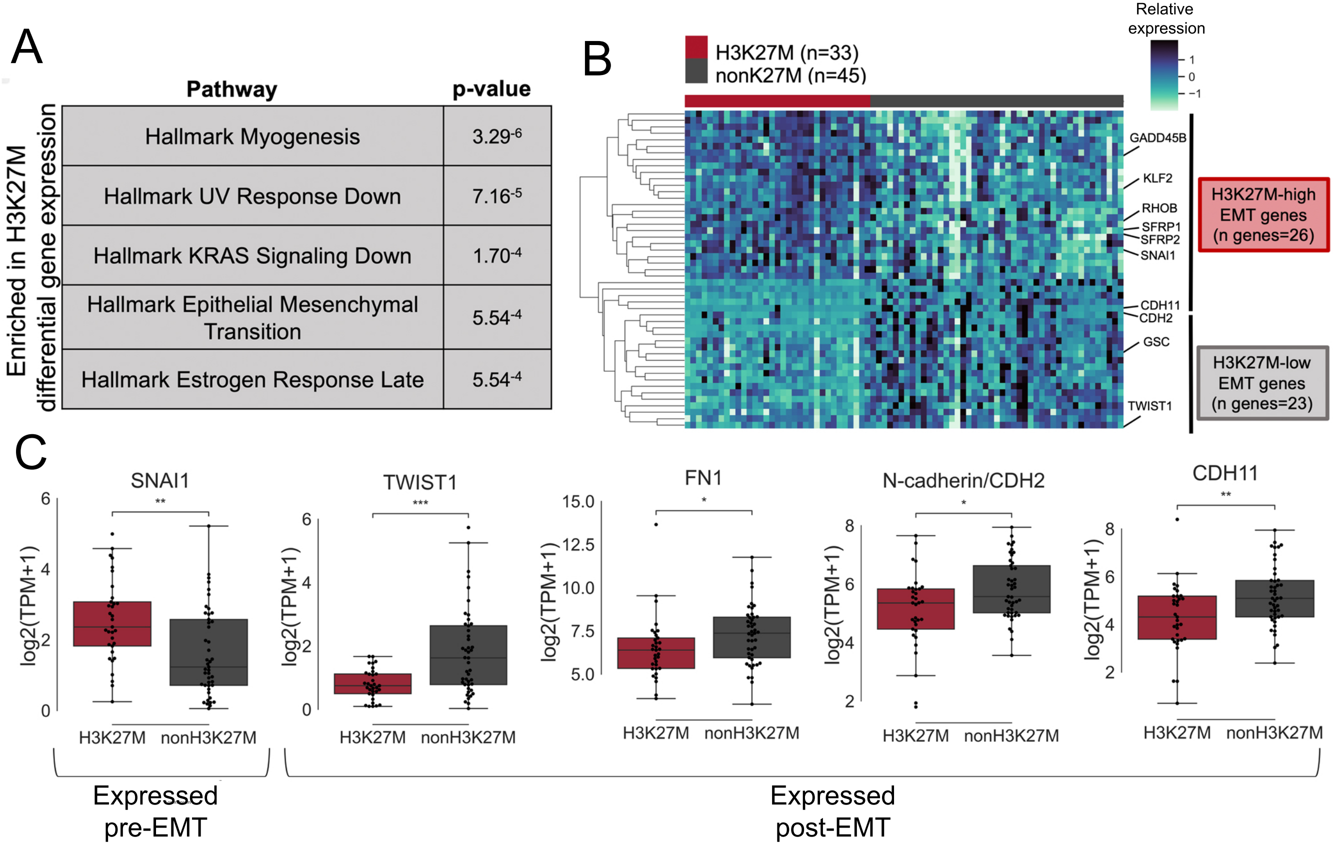

Figure 2

Click here to access/download;Figure;Figure2-small_Revision1.pdf

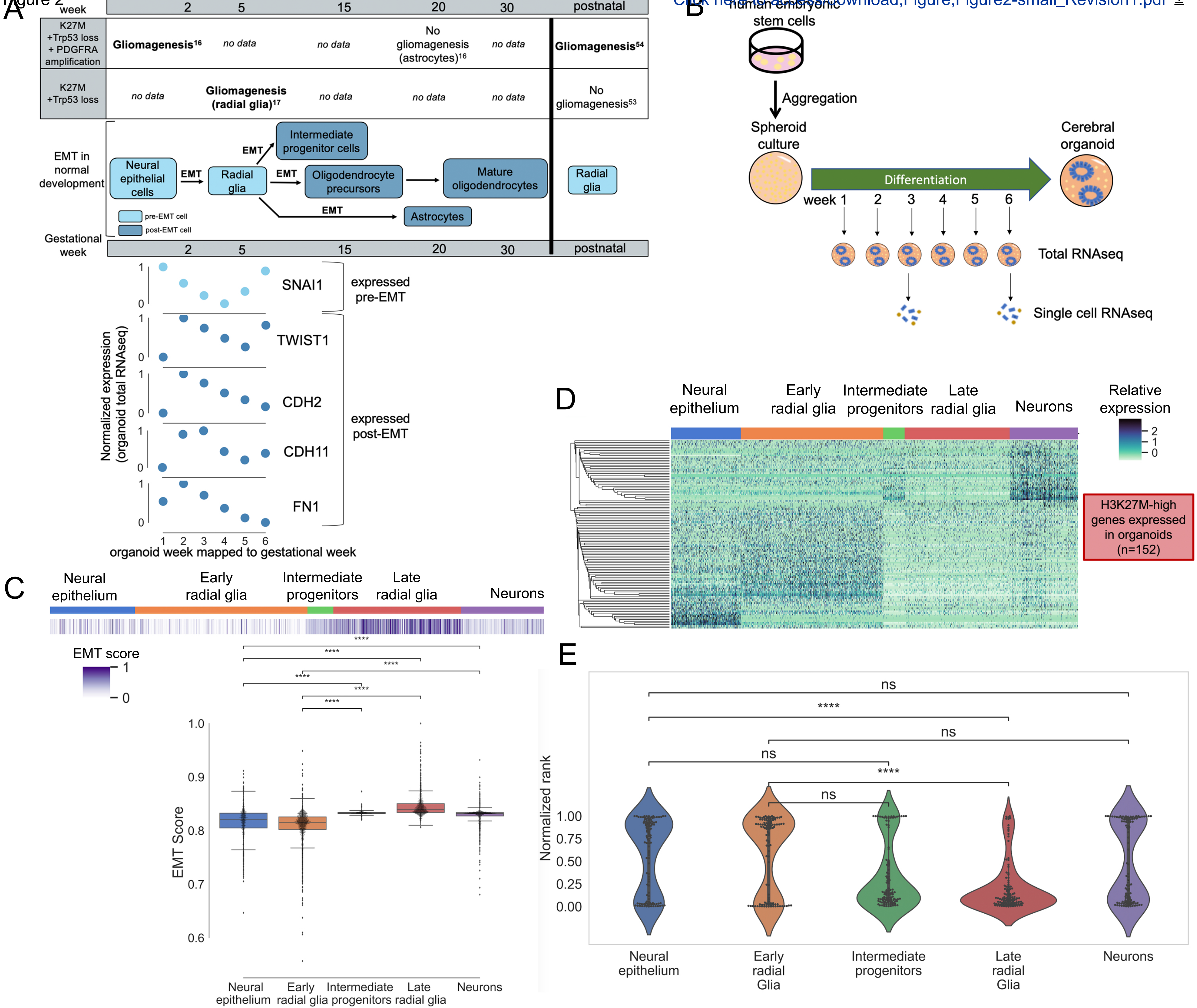

Figure 3

Click here to access/download;Figure;Figure3_Revision1.pdf

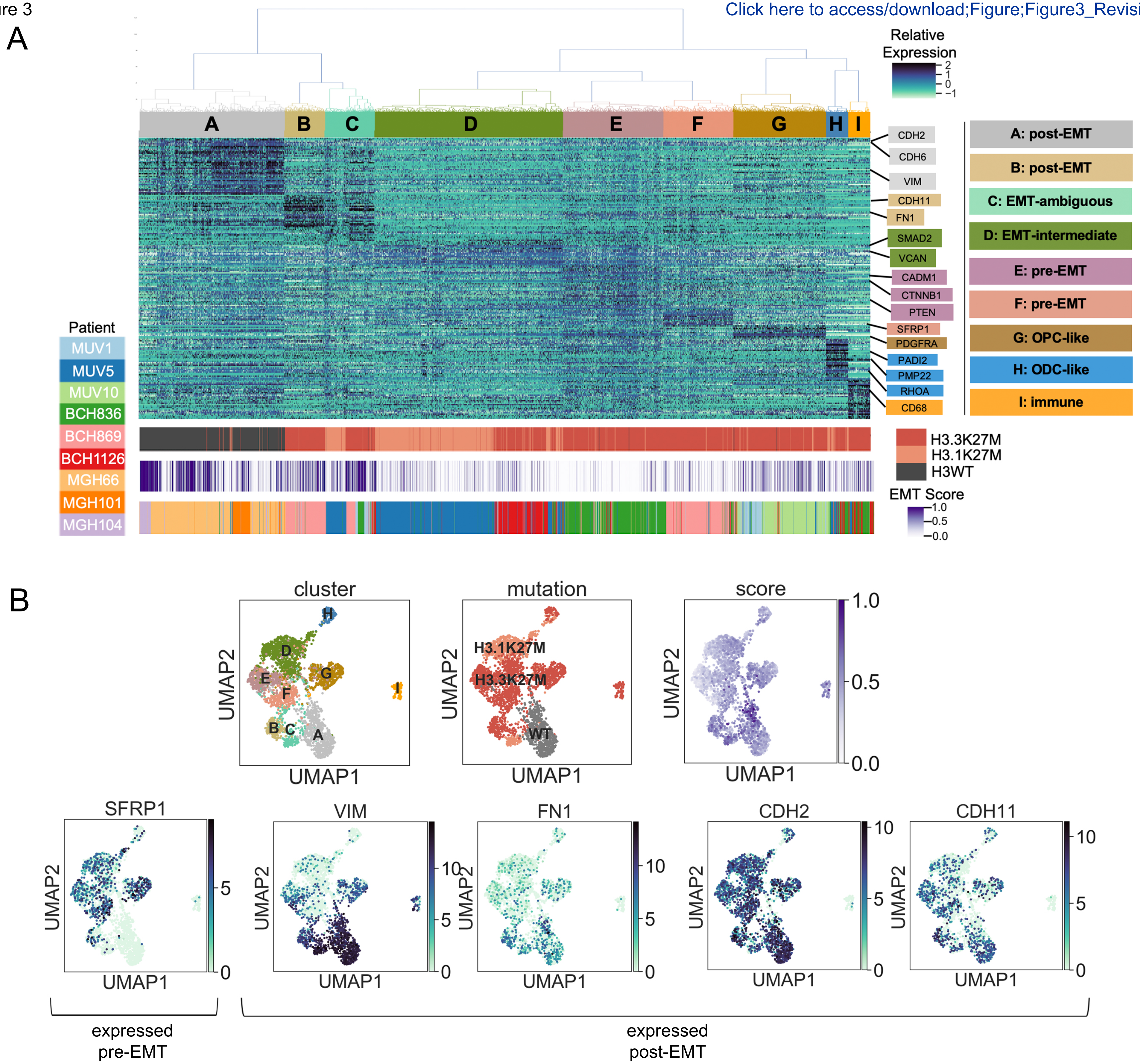

Figure 4

Click here to access/download;Figure;Figure4.pdf

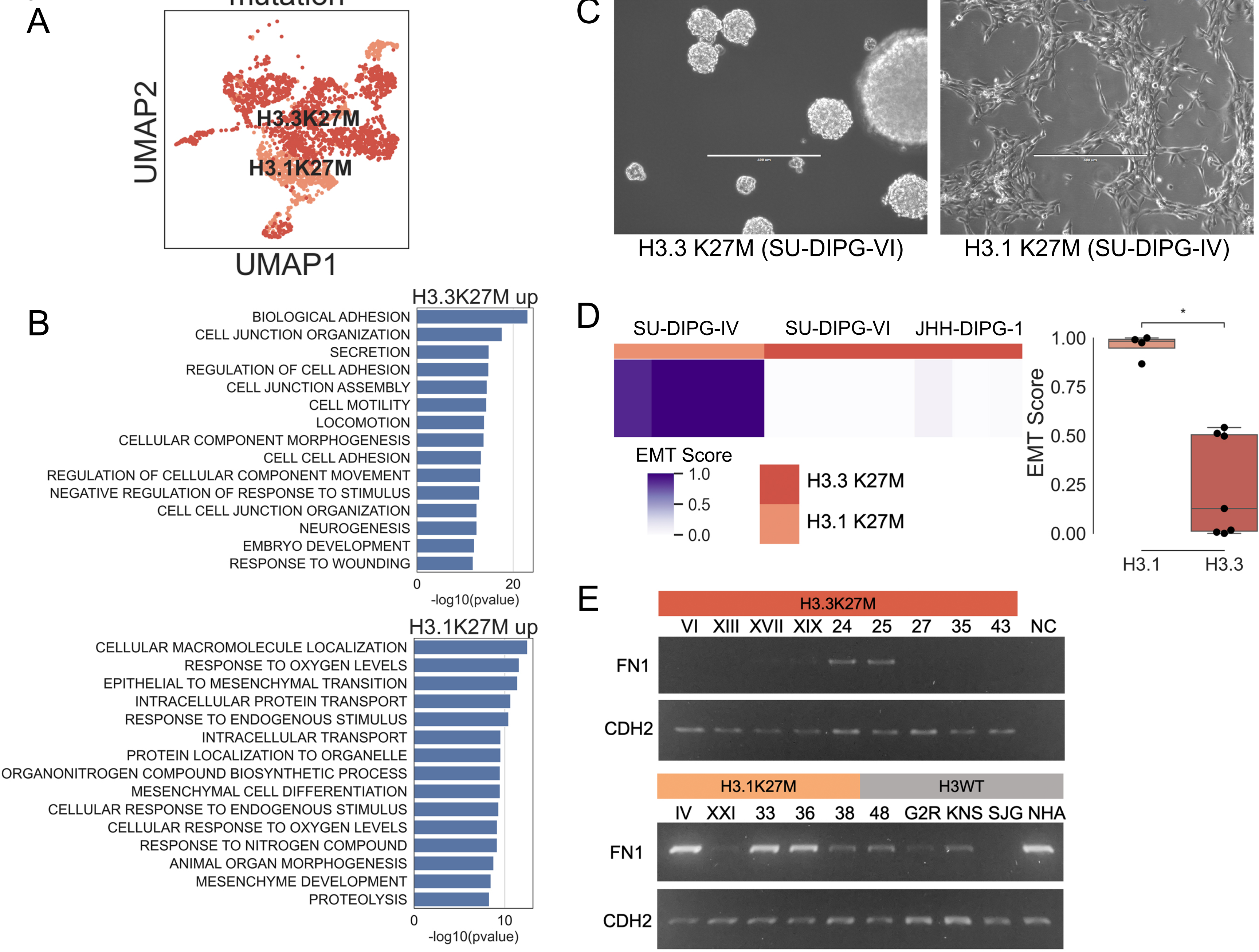

Figure 5

Click here to access/download;Figure;Figure5.pdf

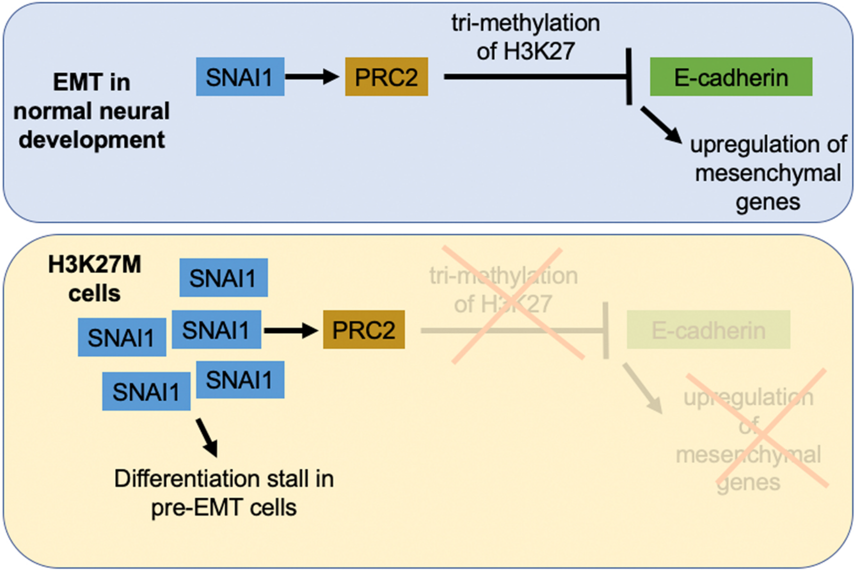

Click here to access/download;Figure;Reviewer-Requested-Figure-1.png

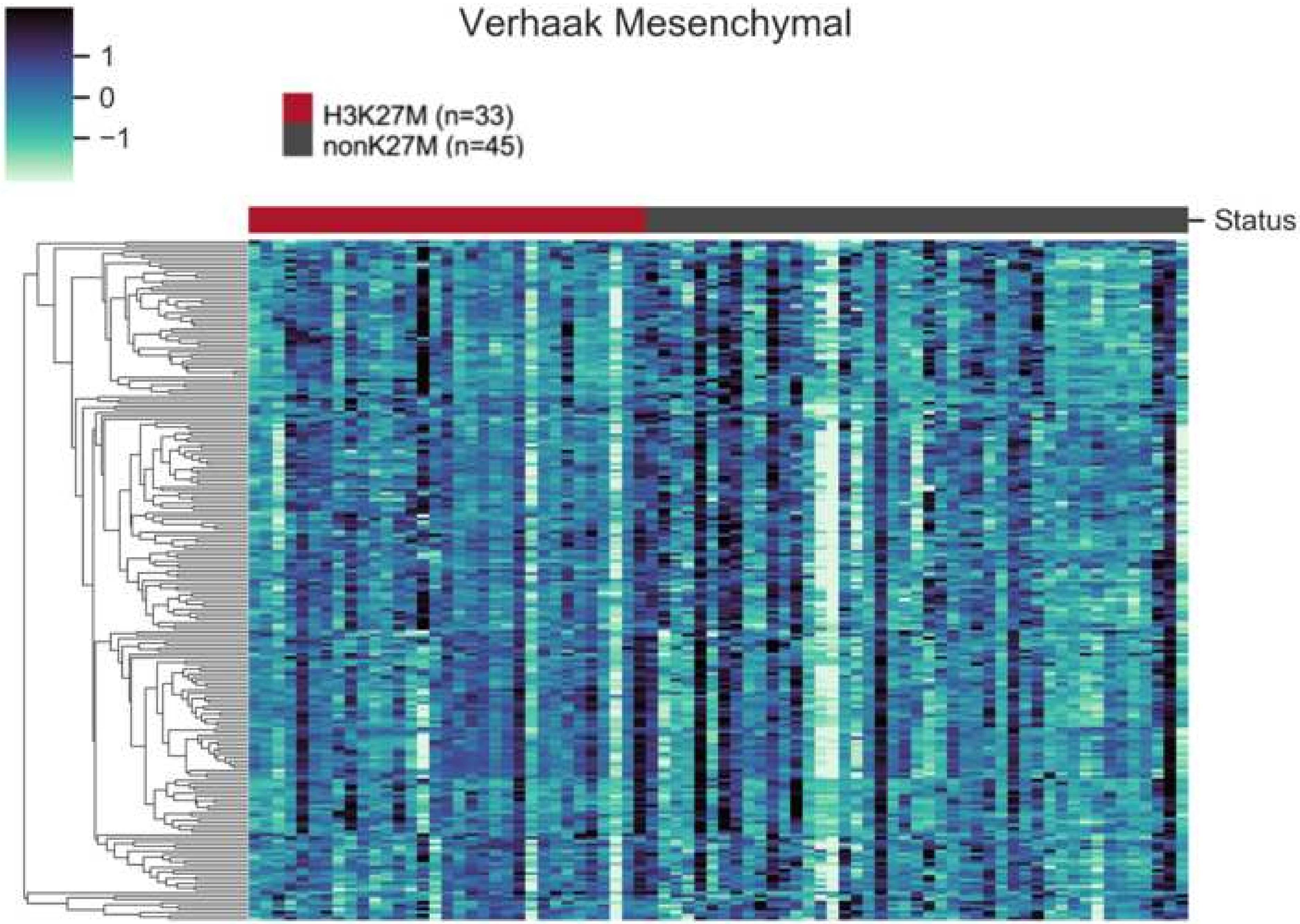

Supplementary Table 1

Click here to access/download
Supplementary Material
Supplementary Table 1.xlsx

Supplementary Table 2

Click here to access/download
Supplementary Material
Supplementary Table 2 Revision1.xlsx

Supplementary Table 3

Click here to access/download
Supplementary Material
Supplementary Table 3.xlsx

Supplementary Table 4

Click here to access/download
Supplementary Material
Supplementary Table 4 Revision1.xlsx

Supplementary Figures

Click here to access/download
Supplementary Material
Supplementary Figures Revision1.pdf
