# Supplementary material for: Identification of a differentiation stall in epithelial mesenchymal transition in histone H3–mutant diffuse midline glioma
Source: Gigascience. 2020 Dec 15;9(12):giaa136. doi: 10.1093/gigascience/giaa136 (PMC7736793; doi:10.1093/gigascience/giaa136)
Supplement: giaa136_Supplemental_Files [file giaa136_supplemental_files.zip › Supplementary Figures Revision1.pdf]

Supplementary Figures

Enriched in H3WT outlier gene expression

Pathway	p-value
Systemic Lupus Erythematosus	2.39^{-21}
Interleukin ECM Regulation	9.97^{-17}
TNF-Alpha Signaling	1.49^{-11}
TGF-Beta ECM Regulation	4.01^{-09}
RNA Polymerase I	4.13^{-09}
Disease Pathway	6.12^{-09}
T-cell Signaling	4.79^{-07}
TWEAK Signaling	1.64^{-06}
Extracellular Matrix	2.98^{-05}
Calcineurin Signaling	3.21^{-05}

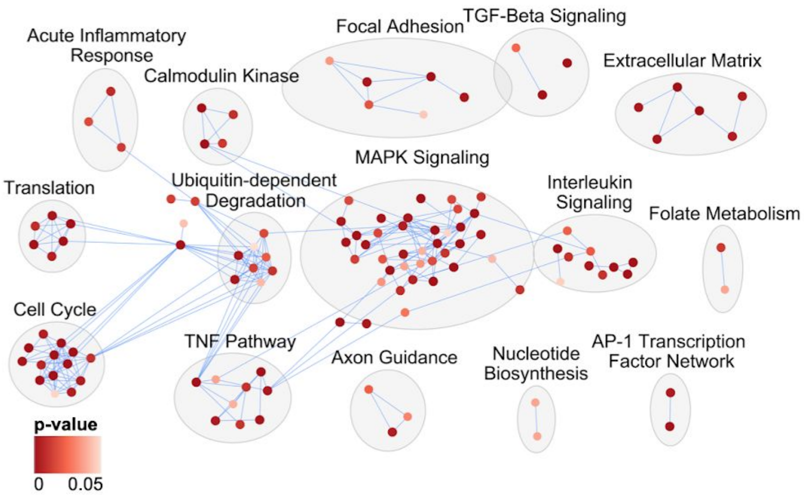

Supplementary Figure 1. Comparative gene expression analysis shows enrichment of post-EMT and mesenchymal pathways in genes with outlier expression in H3WT pHGG tumors. Left: Top 10 pathways from Enrichr gene set enrichment analysis of genes with outlier expression in only nonK27M pHGG samples as compared to a background cohort of similar tumors. Right: Cytoscape EnrichmentMap graph showing clusters of functionally similar pathways enriched for genes with outlier expression in nonK27M pHGG (node p-value < 0.05, edge p-value < 0.38).

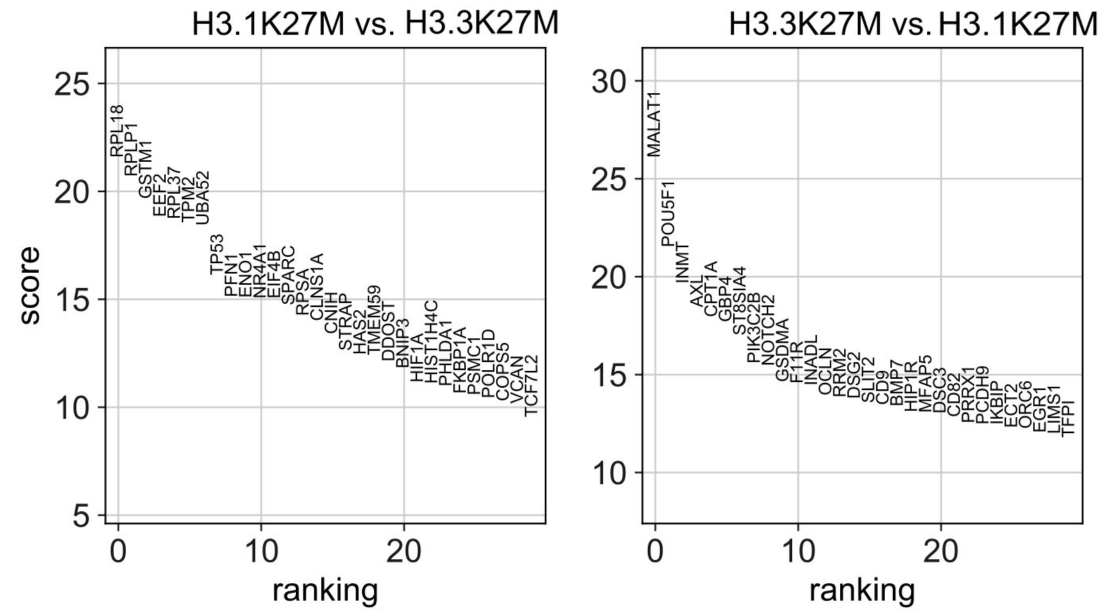

Supplementary Figure 2. Top 30 differentially expressed genes by Wilcoxon rank-sum test comparing H3.3K27M mutant glioma cells with H3.1K27M mutant glioma cells.

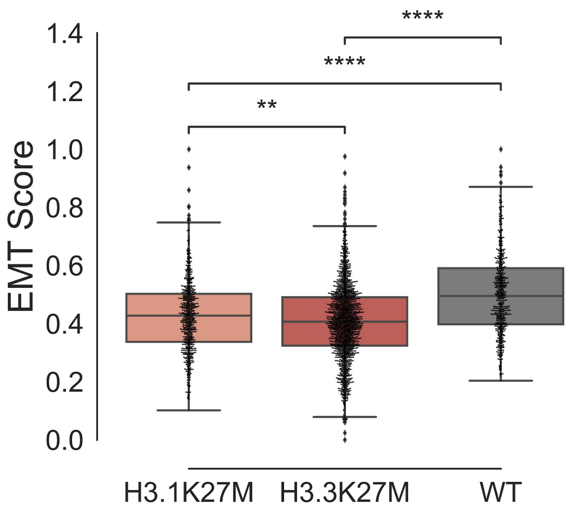

Supplementary Figure 3. Continuum of EMT completeness scores in glioma single cell RNA-seq data.

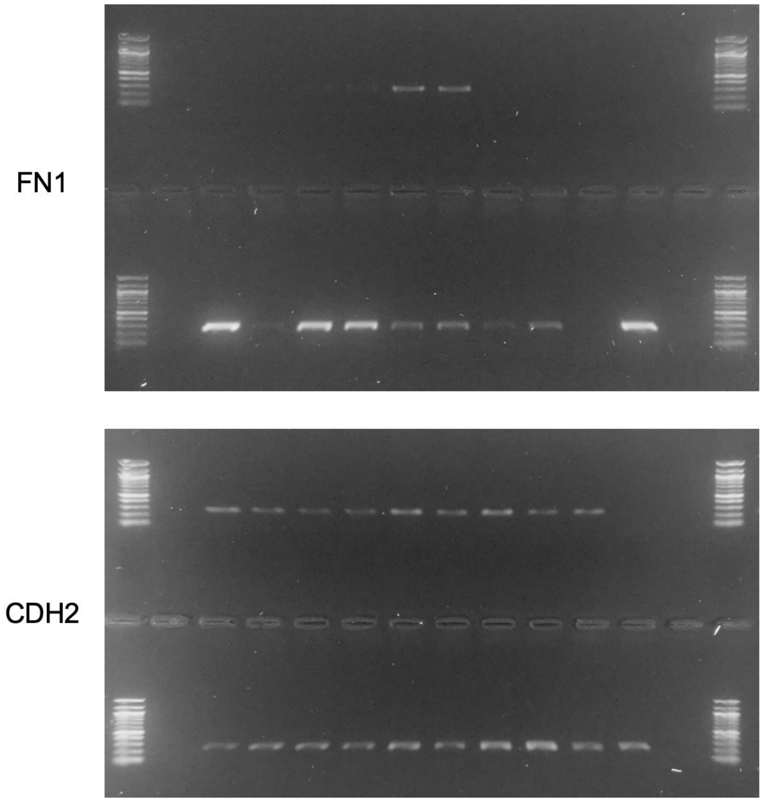

Supplementary Figure 4. Full-length RT-PCR gel images for FN1 and CDH2 quantification. Top row of each gel: SU-DIPG-VI, 13, 17, 19, 24, 25, 27, 35, 43, negative RT-PCR control. Bottom row of each gel: SU-DIPG-IV, 21, 33, 36, 38, 48, pcGBM2R, KNS42, SJG2 and normal human astrocytes hTERT.

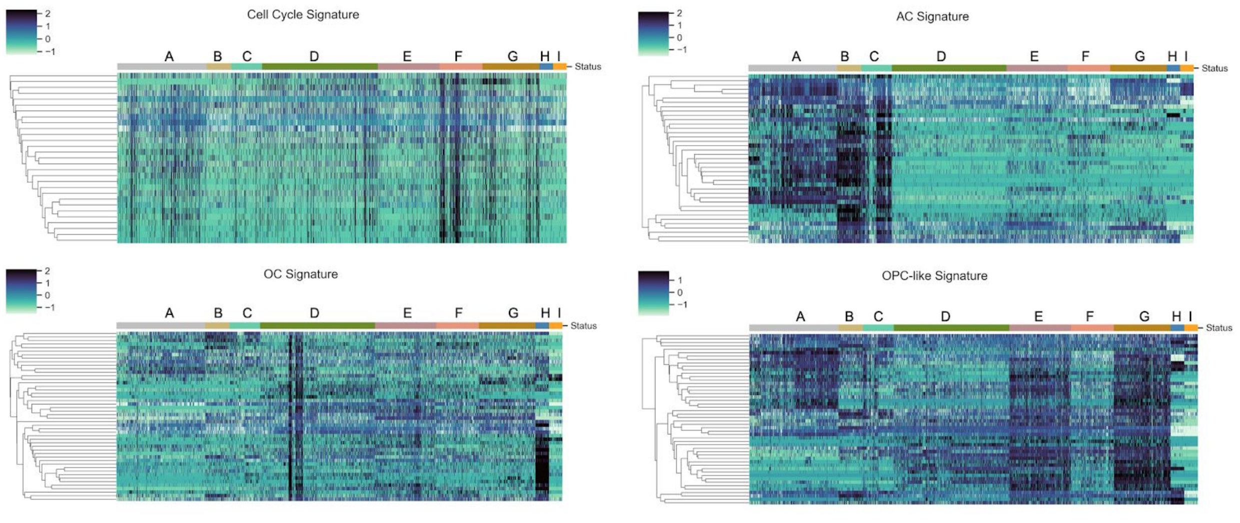

Supplementary Figure 5. Expression of the four signatures identified in Filbin et al., *Science* 2018 in the single cell glioma EMT clusters. (AC=astrocytic, OC=oligodendrocytic, OPC=oligodendrocyte precursor cell).
