# Supplementary material for: Identification of a differentiation stall in epithelial mesenchymal transition in histone H3–mutant diffuse midline glioma
Source: Gigascience. 2020 Dec 15;9(12):giaa136. doi: 10.1093/gigascience/giaa136 (PMC7736793; doi:10.1093/gigascience/giaa136)
Supplement: giaa136_Response_to_Reviewer_Comments_Original_Submission [file giaa136_response_to_reviewer_comments_original_submission.pdf]

Author's Response To Reviewer Comments

Close

Dr. Hongling Zhou
Editor
GigaScience
www.gigasciencejournal.com

Lauren M. Sanders
Postdoctoral Fellow
Molecular, Cell and Developmental Biology
UC Santa Cruz

August 17, 2020

Dear Dr Zhou,

Thank you very much for reviewing our manuscript "Identification of a differentiation stall in epithelial mesenchymal transition in histone H3 mutant diffuse midline glioma".

We thank the reviewers for their time and consideration, and are pleased that both reviewers found our manuscript interesting. We are grateful to the reviewers for their insightful feedback, which has strengthened the manuscript.

Below, we provide point-by-point answers to the reviewers' specific comments. We also include a marked copy of the revised manuscript highlighting the changes along with a clean copy. All page numbers refer to the marked copy.

Again, thank you very much for the opportunity to have our work considered by Giga Science. We look forward to hearing from you soon!

Sincerely,

Lauren M. Sanders

Reviewer #1:
Well written manuscript exploring the gene expression profile differences between H3K27M mutated and non-mutated paediatric high grade glioma. Results suggest that H3K27M mutant pHGG have a pre-EMT gene expression phenotype in comparison to non-mutant tumours. Some queries below.

Differential expression analysis:
1. Could it be stated somewhere in the manuscript that some of the samples used for differential expression analysis are not strictly paediatric and were rather aged <30.
Response:
We have included this statement on page 7.

2. Were the p-values adjusted for multiple testing?
Response:
Yes, a Benjamini Hochberg multiple testing correction was applied; we have included this in the Methods on page 29.

Pan-disease outlier analysis
3. It was unclear which types of cancers were included in the background tumours for this analysis.
Response:
The background cohort for the pan-disease outlier analysis consisted of 37 pediatric gliomas, 19 young

adult gliomas, 18 pediatric glioblastomas and 4 young adult glioblastomas. We have included this information in the Methods (page 26).

RT-PCR
4. please provide annealing temperatures for each primer pair
Response:
The annealing temperature for both primer pairs was 64C. We have included this in the Methods on page 29.

Figure 2A
5. It's not super clear that the previous finding of gliomagenesis in the postnatal period was identified in a pre-EMT cell type, and the finding of no-gliomagenesis occurred in a post-EMT cell type. Could it be made clearer in the figure what cell types these previous experiments were performed in?
Response:
To clarify, Lewis et al., Science 2013 (ref. 62) found that expression of H3.3K27M with p53 loss in neonatal progenitor cells was insufficient for gliomagenesis. Subsequently, Larson et al. Cancer Cell 2019 (ref. 63) found that induction of H3.3K27M, p53 loss and mutant PDGFRA in neonatal progenitor cells did result in spontaneous gliomagenesis. Both experiments involved pre-EMT progenitor cells. We have clarified this in the text on page 12.

EMT gene signature and score
6. Could you please provide more information on how this was developed and calculated.
Response:
The EMT completion gene signature was developed through literature review and contains canonical post-EMT gene markers. Supplementary Table 3 shows the genes in this signature and literature sources for the role of each gene in EMT. The single cell transcriptional score is calculated using a previously published method (Tirosh et al., Science 2016, Filbin et al., Science 2018, and Neftel et al., Cell 2019). A control gene set 100 times the length of the gene set of interest is produced by binning all genes into 30 bins of aggregate expression levels. For each gene in the gene set of interest, 100 genes are randomly selected from that gene’s expression bin. This creates a control gene set with a comparable expression distribution, so that its expression provides a background gene set that is 100 times larger than the gene set of interest. For each cell, the average expression of the gene set of interest is compared to the average expression of the control gene set to create a raw score for that cell. The scores are then normalized across the dataset. The code for our implementation of this method is publicly available at: github.com/lauren-sanders/EMT-paper/

Figure 3A
7. Would it be possible to include which of the 8 individual gliomas each cell is from as an additional coloured bar? It would be interesting to see if the cells clustered by patient as well as mutation.
Response:
We have included this bar in Figure 3A.

8. Additionally could you provide the number of cells sequenced and analysed per glioma for this figure.
Response:
The number of cells per glioma is below and we have added this information to the Methods (page 27).
MUV1: 146
MUV5: 708
MUV10: 286
BCH836: 527
BCH869: 492
BCH1126: 299
MGH66: 442
MGH101: 92
MGH104: 65

Line 92
9. I think there may be a word missing in this line: "resulting in H3K27 trimethylation of key epithelial genes such as concurrently upregulating mesenchymal genes"
Response:
Thank you; we have reworded the sentence.

Reviewer #2:
The authors searched a subset of publicly available pediatric high-grade gliomas datasets to compare differentially expressed genes between H3K27M mutant diffuse gliomas and H3K27M wild-type gliomas. They identified an early EMT signature overexerted in the former compared to the latter.

MAJOR COMMENTS
1. Diffuse midline glioma whether H3K27M mutant are different from hemispheric glioma especially in their invasive phenotype. As such, comparing these entity is proved to show a difference in EMT as invasion and EMT are so linked. Therefore, the authors can not infer that the difference shown here is due to the H3K27M mutation per se. To support this statement, they should either perform KO or KD experiments in H3K27M mutant cells or KI experiments in H3K27M wild-type cells. Alternatively, separating in the data set diffuse gliomas from the more circumscribed gliomas would be helpful. Ideally a significant data set from diffuse gliomas without H3K27M mutation may be a better control. If these experiments can not be performed, the statement should be then softened.

Response:
We thank the reviewer for raising a very important point and providing some excellent recommendations for additional analysis. In order to address this issue, we have repeated our differential expression analysis with a cohort of DIPG samples containing no hemispheric glioma samples, in order to clarify the role of H3K27M mutation after removing possible histological or location factors. The results of this analysis are consistent with the original findings. We describe this analysis on page 10 of the manuscript, and we also provide a caveat in the Discussion (page 24) and suggest future studies that could more clearly describe the role of H3K27M mutation alone in the observed EMT-related transcriptional signature. We provide the description of the additional DIPG-specific analysis, as well as the caveat in the Discussion, below:

Additional DIPG-specific Analysis: (page 10)
Finally, because EMT is associated with invasiveness in gliomas, and diffuse midline glioma are by nature more invasive than hemispheric glioma, we performed an additional analysis restricted to diffuse intrinsic pontine glioma (DIPG) to elucidate the role of the H3K27M mutation in the observed EMT-related transcriptional profiles. The goal of this analysis was to remove any potential histological or location signal that may be influencing the EMT-related gene expression. We used 10 H3 wild-type DIPG samples and 47 H3K27M DIPG samples from Treehouse cancer compendium v11.
Limma differential expression analysis revealed 48 genes with higher expression in H3K27M DIPG compared to nonK27M DIPG (Supplementary Table 2). We again computed statistical overlap of these genes with 9 gene sets relating to epithelial cells and early brain development, and found significant overlap with 4 of the 9 gene sets (pvalue<0.1, Supplementary Table 2). The enrichment of 48 randomly selected genes in these 9 gene sets were not significant (Supplementary Table 2).

Caveat in the Discussion (page 24):
"Additionally, a limitation of our study is that it is difficult to isolate the role of the H3K27M mutation from other factors such as histology and tumor location. Future studies will focus on EMT-related transcriptional programs in cellular models with inducible H3K27M expression to further characterize the molecular interplay between the H3K27M mutation and EMT in developing brain cells."

2. A comparison with adult type gliomas of the mesenchymal subtype would be interesting as well to tease out the real role of H3K27M mutation in this phenotype.

Response:
We examined the expression of the adult glioma mesenchymal subtype gene signature (Verhaak et al., Cancer Cell 2010) in our pHGG cohort (see Reviewer Requested Figure 1). The H3K27M and nonK27M pHGG samples display similar expression of the adult mesenchymal signature, such that the expression of these genes does not separate them into different subtypes. This may be explained by the numerous differences between adult glioblastomas and pediatric diffuse midline gliomas. Adult glioblastomas have a higher mutation rate than pediatric gliomas (Schwartzentruber et al., Nature 2012), and the vast majority are supratentorial (Jones and Baker, Nat Rev Cancer 2014), while by definition DMGs occur along the midline. Additionally, treatments that have shown efficacy in adult GBM are notably ineffective in DMGs (Jones et al., Neuro Oncol 2016).

Additionally, the adult GBM Mesenchymal subtype is predominantly characterized by mutations or deletions of NF1, a gene which is rarely altered in pediatric glioma. Moreover, this subtype expresses a

combination of mesenchymal and astrocytic markers linked to dedifferentiated tumors. While dedifferentiation is well characterized in adult tumors since they arise in fully developed tissues, pediatric gliomas arise during embryonic development and are thought to represent a differentiation stall rather than dedifferentiation events (Filbin and Monje, Nat Med 2019).

Also, while the EMT is associated with proliferation and invasion in some adult cancers (Marcucci et al., Nat Rev Drug Discov 2016), here we refer to the EMT as a developmental process which must occur multiple times during normal neuronal development. This process is controlled by the H3K27me3 epigenetic mark, and we hypothesize that lack of H3K27me3 may prevent full completion of EMT at one or multiple timepoints during early brain development. Here, we follow up on previous work in the field which shows that early neural or glial precursor cells are in a susceptible transcriptional state and undergo a differentiation stall upon the occurrence of H3K27M. Because embryonic neural progenitor/stem cells undergo EMT-like processes during the formation of the neural plate and neural tube, we hypothesized that early neural or glial precursors poised to undergo an EMT as part of normal development are in a susceptible transcriptional state for gliomagenesis. Importantly, here we discuss EMT as a normal developmental process implicated in the early stages of H3K27M gliomagenesis, separate and distinct from the EMT as a process utilized by malignant cells undergoing metastasis, which may also occur later in pHGG tumor growth, as noted in Puget et al., PLoS One 2012.

We have clarified this throughout the manuscript by removing the use of the words “epithelial” or “mesenchymal” when referring to the EMT-like pHGG phenotypes that we observe.

3. The concept of EMT in gliomas can not be extrapolated form the data acquired in epithelial tumors. Most glioma gene expression profiles are mesenchymal and not epithelial at all while the so called mesenchymal subtype is even more mesenchymal (Iser et al, Med Res Rev 2017). The term is therefore misleading, should be used with caution and the EMT datasets from epithelial cancers should not be applied. The genes defining mesenchymal subset of GBM may have been more appropriate.

Response:
Thank you to the reviewer for an excellent point. To address this comment, we have removed genes derived from EMT datasets from epithelial cancers, and retain only genes from datasets annotated by EMT as a cellular developmental process. This change is reflected in Supplementary Table 2 “EMT master gene list” and Figures 1B and 3A, as well as in the text on page 8. As discussed in point 2, the genes defining the mesenchymal subtype of adult GBM may be less relevant for studying EMT as a developmental process in the brain.

MINOR COMMENTS
- Differential expression analysis of pediatric gliomas with and without H3K27M mutation reveals deregulation of genes involved in epithelial-mesenchymal transition.

4. The authors should provide a reference that indicate that EMT is orchestrated by H3K27me3 marks (I found one reference in glioma Zhou et al, Oncol Lett 2019, the rest is in epithelial cancers).
Response:
Thank you to the reviewer for identifying an excellent reference. We have included this reference on page 4.

5. The limitation of GSEA datasets is that in most of the cases they are not oriented by over-expression or repression of the gene in the described pathway.
Response:
In order to overcome this limitation, we performed differential expression analysis first, then used gene set enrichment analysis to investigate gene sets enriched in overexpressed genes in the H3K27M glioma cohort.

6. The authors claim that over expressed genes in H3K27M mutant genes are associated with epithelial-like states and normally unregulated prior EMT while those under expressed are associated with the mesenchymal state, this is in contradiction with the adult literature in gliomas and also previous publications (Meel et al, Cell Mol Life Sci 2018 or Puget et al, PLoS ONE 2012). They should provide statistically meaningful data and not only exemples.

Response:
In order to provide statistically meaningful analysis of the genes over-expressed in H3K27M mutant gliomas, we performed additional gene set enrichment analysis using the EMT genes over-expressed in H3K27M mutant glioma. Our results are consistent with the rest of our original findings, and show significant enrichment in gene sets related to early development, neurodevelopment, epithelial proliferation and epithelium development. We have included these results in the manuscript on page 9.

With respect to the publications mentioned by the reviewer, Meel et al 2018 primarily reviews the data on EMT in adult gliomas; there is limited data on the expression of EMT genes in DIPGs. They also acknowledge differences in the role of EMT genes between adult gliomas and DIPGs. In figure 4 of their paper, the authors show that in 6/8 pro-EMT genes examined, their expression in DIPGs is lower than or equal to the expression in adult GBM. Those results concur with our data in H3K27M gliomas. In addition, Meel et al specifically calls for more research in EMT in pediatric gliomas, which is the focus of our study.

Puget 2012 identifies a subset of DIPG samples (whose H3K27M status is unknown) that is enriched for the mesenchymal adult GBM subtype (mentioned above). They also examine expression of a list of 53 transcription factors associated with EMT in adult HGGs. It appears from figure 3A that some portion of these genes has relatively low expression in this mesenchymal subset of DIPGs, which is consistent with our analysis as well. While Puget was crucial in discovering a role for EMT in DIPGs, our study provides a novel contribution by focusing on the role of H3K27M and the concomitant loss of H3K27me3 in the expression of EMT-related genes.

We discuss both of these previous studies in our manuscript (Meel et al 2018 referenced on page 22, Puget 2012 referenced on pages 20 and 21 as discussed in points 12 and 14).

- H3K27M-mediated gliomagenesis is associated with pre-EMT cell types.
7. If I understood the experiment correctly, the authors show that H3K27M mutant gliomas have a pre-EMT signature and that this signature is present in the developing brain in cells in pre-EMT stage. This is a rather tautologic demonstration. EMT and stem cell state are really correlated in these gliomas where the bulk of the tumor cells are stem-like cells (Filbin et al, Science 2018). This could well be another hypothesis. This organoid experiment does not clarify it in my view.
Response:
To clarify, the organoid experiment serves to identify specific cell stages in the developing brain which are transcriptionally similar to H3K27M glioma. We employed the organoid data in order to investigate the hypothesis that H3K27M gliomas are similar to pre-EMT developing cells, and this analysis shows a transcriptional similarity between H3K27M gliomas and pre-EMT brain cell stages. We have clarified this experiment in the text on page 14.

- Single-cell profiling of H3K27M gliomas reveals groups of cells at different stages of EMT.
8. Number of samples are really low and lower than in the the reference paper where this data was extracted from, why?
Response:
The reference paper analyzed 6 K27M gliomas and 3 IDH-WT / H3-WT GBMs, all of which were publicly available at GSE102130 and we have included all of these data in our analysis. Although the reference paper does reference additional IDH-mutant astrocytoma and IDH-mutant oligodendroglioma samples, these data were not publicly released and would not have been relevant for our analysis.

9. How do the A, F, G, H clusters compare with the stemless signature used by Filbin et al?
Response:
We show in Supplementary Figure 5 the expression of the 4 signatures identified by Filbin et al., Science 2018 (cell cycle, astrocytic, oligodendrocytic, and OPC-like) in the clusters identified in the EMT analysis. Consistent with our analysis, the mature astrocytic signature is enriched in “post-EMT” clusters A and B. The mature oligodendrocytic signature is enriched in cluster H, consistent with our finding that cluster H is ODC-like. The oligodendrocyte precursor stem-like signature is enriched in cluster E, consistent with our finding that cluster E is less differentiated and “pre-EMT”, and and in “OPC-like” cluster G.

10. The occurence of tumor cells in different state of EMT is in contradiction with the claim that H3K27M tumors are in a differentiation stall, isn't?
Response:
The single cell RNA-seq analysis refines the hypothesis that H3K27M tumors undergo a differentiation

stall during developmental EMT events. While both H3.3K27M and H3.1K27M cells appear to be less mature than H3WT cells, the two mutations result in slightly different EMT-related transcriptional signatures, with H3.3K27M cells expressing a more primitive signature than H3.1K27M cells.

11. The mixing of different H3K27M tumors is not appropriate, especially with the many differences already shown between H3.1 and H3.3K27M tumors (Nagaraja, Castel for exemple).
Response:
There are indeed many known differences between H3.1K27M and H3.3K27M tumors as noted by the reviewer. However, for our study, we started out by hypothesizing that both H3.1K27M and H3.3K27M mutant gliomas would be different from the H3WT tumors because they both have loss of H3K27 trimethylation. Since we included both mutation types and wild-type cells in our unsupervised single cell clustering, we were able to identify different transcriptional signatures which would not have been apparent if we had kept the mutation types separate.

- Histone H3.1K27M glioma cells may represent a more advanced stage of EMT than H3.3K27M glioma cells.
12. EMT has been observed in DIPG (the prototypic H3K27M diffuse midline glioma) since 2012 by Puget et al. Albeit the histone H3 mutation was not known. The mesenchymal subtype described in this paper resembles the H3.1K27M subgroup mentioned here. This may be discussed later. Moreover, the H3.1K27M DIPG gene expression profile has been already shown to be enriched with the mesenchymal GBM signature, this old data support their finding and should not be omitted in the discussion.
Response:
Thank you to the reviewer for the reminder of two very relevant studies. We have included a reference of the EMT-related findings of Puget et al (PLoS One 2012) and Castel et al (Acta Neuropathol 2015) on page 21. The new text is below:
"Our computational and in vitro observations are consistent with a recent study indicating that H3.1K27M tumor cells are overall more differentiated than H3.3K27M tumor cells (Nagaraja et al., Mol Cell 2019). Our results are also consistent with previous studies on EMT in pediatric gliomas which first found a mesenchymal subtype of DIPG and subsequently discovered that H3.1K27M mutant gliomas express genes associated with a more mesenchymal subtype of glioblastoma(Puget et al., PLoS One 2012, Castel et al., Acta Neuropathol 2015)."

13. There is also data out there showing a different distribution of H3K27me3 marks in DIPG with respect to the histone H3 variant mutated at K27 (Castel et al, Acta Neuropathol Comm 2018) and this data is not in line with the statement that in H3.1K27M induced H3K27me3 loss is diffuse. Please comment or amend.
Response:
We have amended our description of the trimethylation loss in the two mutation types on page 19 to be consistent with the findings of Castel et al, Acta Neuropathol Comm 2018, and we have included a reference to this paper. The amended text is below:
"Normally, histone H3.3 is preferentially located at active chromatin (Szenker et al., Cell Res 2011, Goldberg et al., Cell 2010, Nagaraja et al., Mol Cell 2019). This leads to distinct patterns of epigenetic reprogramming in each histone variant, where loss of the H3.3K27me3 mark is directly correlated with areas of H3.3 genomic enrichment, while H3.1K27me3 loss is higher at intergenic regions (Nagaraja et al., Mol Cell 2019, Castel et al., Acta Neuropathol Commun 2018)."

14. The single cell data comparing H3.1 and H3.2K27M cells confirms the previous finding of Castel et al (Acta Neuropathol 2015). Why did the authors not consider these findings?
Response:
Thank you to the reviewer for the suggestion and reference. As noted in point 12, we have included a discussion of the findings of Castel et al (Acta Neuropathol 2015) on page 21.

Discussion
15. In Grasso's paper (Nature Med 2015), it was shown that HDACi treatment was reverting the mesenchymal phenotype of the tumors cells in vitro. The authors should comment this information as well since it is supporting the interplay between H3K27Ac/H3K27me3 and mesenchymal drift.
Response:
Thank you to the reviewer for the suggestion. We now include a discussion of the findings of Grasso et al., Nature Med 2015 on page 23. The new text is below:
"Histone deacetylase inhibitor treatment of in vitro pHGG cells reversed mesenchymal phenotypes, in keeping with a model in which the interplay between H3K27 acetylation and methylation controls EMT-related transcriptional states(Grasso et al., Nature Med 2015)."

Close
