# Supplementary material for: Identification of a differentiation stall in epithelial mesenchymal transition in histone H3–mutant diffuse midline glioma
Source: Gigascience. 2020 Dec 15;9(12):giaa136. doi: 10.1093/gigascience/giaa136 (PMC7736793; doi:10.1093/gigascience/giaa136)
Supplement: giaa136_Reviewer_1_Report_Original_Submission — Alexandra Sexton Oates -- 5/20/2020 Reviewed [file giaa136_reviewer_1_report_original_submission.pdf]

Reviewer Report

Title: Identification of a differentiation stall in epithelial mesenchymal transition in histone H3 mutant diffuse midline glioma

Version: Original Submission **Date:** 5/20/2020

Reviewer name: Alexandra Sexton Oates

Reviewer Comments to Author:

Well written manuscript exploring the gene expression profile differences between H3K27M mutated and non-mutated paediatric high grade glioma. Results suggest that H3K27M mutant pHGG have a pre-EMT gene expression phenotype in comparison to non-mutant tumours. Some queries below.
Differential expression analysis:
- Could it be stated somewhere in the manuscript that some of the samples used for differential expression analysis are not strictly paediatric and were rather aged <30.
- Were the p-values adjusted for multiple testing?
Pan-disease outlier analysis
- It was unclear which types of cancers were included in the background tumours for this analysis
RT-PCR
- please provide annealing temperatures for each primer pair
Figure 2A
- It's not super clear that the previous finding of gliomagenesis in the postnatal period was identified in a pre-EMT cell type, and the finding of no-gliomagenesis occurred in a post-EMT cell type. Could it be made clearer in the figure what cell types these previous experiments were performed in?
EMT gene signature and score
- Could you please provide more information on how this was developed and calculated.
Figure 3A
- Would it be possible to include which of the 8 individual gliomas each cell is from as an additional coloured bar? It would be interesting to see if the cells clustered by patient as well as mutation.
- Additionally could you provide the number of cells sequenced and analysed per glioma for this figure.
Line 92
- I think there may be a word missing in this line: "resulting in H3K27 trimethylation of key epithelial genes such as concurrently upregulating mesenchymal genes"

Methods

Are the methods appropriate to the aims of the study, are they well described, and are necessary controls included? Choose an item.

Conclusions

Are the conclusions adequately supported by the data shown? Choose an item.

Reporting Standards

Does the manuscript adhere to the journal's guidelines on [minimum standards of reporting?]() Choose an item.

Choose an item.

Statistics

Are you able to assess all statistics in the manuscript, including the appropriateness of statistical tests used? Choose an item.

Quality of Written English

Please indicate the quality of language in the manuscript: Choose an item.

Declaration of Competing Interests

Please complete a declaration of competing interests, considering the following questions:

- Have you in the past five years received reimbursements, fees, funding, or salary from an organisation that may in any way gain or lose financially from the publication of this manuscript, either now or in the future?
- Do you hold any stocks or shares in an organisation that may in any way gain or lose financially from the publication of this manuscript, either now or in the future?
- Do you hold or are you currently applying for any patents relating to the content of the manuscript?
- Have you received reimbursements, fees, funding, or salary from an organization that holds or has applied for patents relating to the content of the manuscript?
- Do you have any other financial competing interests?
- Do you have any non-financial competing interests in relation to this paper?

If you can answer no to all of the above, write 'I declare that I have no competing interests' below. If your reply is yes to any, please give details below.

I declare that I have no competing interests.

I agree to the open peer review policy of the journal. I understand that my name will be included on my report to the authors and, if the manuscript is accepted for publication, my named report including any attachments I upload will be posted on the website along with the authors' responses. I agree for my report to be made available under an Open Access Creative Commons CC-BY license (http://creativecommons.org/licenses/by/4.0/). I understand that any comments which I do not wish to be included in my named report can be included as confidential comments to the editors, which will not be published.

Choose an item.

To further support our reviewers, we have joined with Publons, where you can gain additional credit to further highlight your hard work (see: https://publons.com/journal/530/gigascience). On publication of this paper, your review will be automatically added to Publons, you can then choose whether or not to claim your Publons credit. I understand this statement.

Yes Choose an item.
