# Supplementary material for: Identification of a differentiation stall in epithelial mesenchymal transition in histone H3–mutant diffuse midline glioma
Source: Gigascience. 2020 Dec 15;9(12):giaa136. doi: 10.1093/gigascience/giaa136 (PMC7736793; doi:10.1093/gigascience/giaa136)
Supplement: giaa136_Reviewer_2_Report_Original_Submission — Jacques Grill -- 5/23/2020 Reviewed [file giaa136_reviewer_2_report_original_submission.pdf]

Reviewer Report

Title: Identification of a differentiation stall in epithelial mesenchymal transition in histone H3 mutant diffuse midline glioma

Version: Original Submission **Date:** 5/23/2020

Reviewer name: Jacques Grill

Reviewer Comments to Author:

The authors searched a subset of publicly available pediatric high-grade gliomas datasets to compare differentially expressed genes between H3K27M mutant diffuse gliomas and H3K27M wild-type gliomas. They identified an early EMT signature overexerted in the former compared to the latter.
MAJOR COMMENTS
Diffuse midline glioma whether H3K27M mutant are different from hemispheric glioma especially in their invasive phenotype. As such, comparing these entity is proved to show a difference in EMT as invasion and EMT are so linked. Therefore, the authors can not infer that the difference shown here is due to the H3K27M mutation per se. To support this statement, they should either perform KO or KD experiments in H3K27M mutant cells or KI experiments in H3K27M wild-type cells. Alternatively, separating in the data set diffuse gliomas from the more circumscribed gliomas would be helpful. Ideally a significant data set from diffuse gliomas without H3K27M mutation may be a better control. If these experiments can not be performed, the statement should be then softened.
A comparison with adult type gliomas of the mesenchymal subtype would be interesting as well to tease out the real role of H3K27M mutation in this phenotype.
The concept of EMT in gliomas can not be extrapolated form the data acquired in epithelial tumors. Most glioma gene expression profiles are mesenchymal and not epithelial at all while the so called mesenchymal subtype is even more mesenchymal (Iser et al, Med Res Rev 2017). The term is therefore misleading, should be used with caution and the EMT datasets from epithelial cancers should not be applied. The genes defining mesenchymal subset of GBM may have been more appropriate.
MINOR COMMENTS
- Differential expression analysis of pediatric gliomas with and without H3K27M mutation reveals deregulation of genes involved in epithelial-mesenchymal transition.
The authors should provide a reference that indicate that EMT is orchestrated by H3K27me3 marks (I found one reference in glioma Zhou et al, Oncol Lett 2019, the rest is in epithelial cancers).
The limitation of GSEA datasets is that in most of the cases they are not oriented by over-expression or repression of the gene in the described pathway.
The authors claim that over expressed genes in H3K27M mutant genes are associated with epithelial-like states and normally unregulated prior EMT while those under expressed are associated with the mesenchymal state, this is in contradiction with the adult literature in gliomas and also previous publications (Meel et al, Cell Mol Life Sci 2018 or Puget et al, PLoS ONE 2012). They should provided statistically meningful data and not only exemples.
- H3K27M-mediated gliomagenesis is associated with pre-EMT cell types.

If I understood the experiment correctly, the authors show that H3K27M mutant gliomas have a pre-EMT signature and that this signature is present in the developing brain in cells in pre-EMT stage. This is rather tautologic demonstration. EMT and stem cell state are really correlated in these gliomas where the bulk of the tumor cells are stem-like cells (Filbin et al, Science 2018). This could well be another hypothesis. This organoid experiment does not clarify it in my view.
- Single-cell profiling of H3K27M gliomas reveals groups of cells at different stages of EMT.
Number of samples are really low and lower than in the the reference paper where this data was extracted from, why?
How do the A, F, G, H clusters compare with the stemless signature used by Filbin et al?
The occurence of tumor cells in different state of EMT is in contradiction with the claim that H3K27M tumors are in a differentiation stall, isn't?
The mixing of different H3K27M tumors is not appropriate, especially with the many differences already shown between H3.1 and H3.3K27M tumors (Nagaraja, Castel for exemple).
- Histone H3.1K27M glioma cells may represent a more advanced stage of EMT than H3.3K27M glioma cells.
EMT has been observed in DIPG (the prototypic H3K27M diffuse midline glioma) since 2012 by Puget et al. Albeit the histone H3 mutation was not known. The mesenchymal subtype described in this paper ressembles the H3.1K27M subgroup mentioned here. This may be discussed later. Moreover, the H3.1K27M DIPG gene expression profile has been already shown to be enriched with the mesenchymal GBM signature, this old data support their finding and should not be omitted in the discussion. There is also data out there showing a different distribution of H3K27me3 marks in DIPG with respect to the histone H3 variant mutated at K27 (Castel et al, Acta Neuropathol Comm 2018) and this data is not in line with the statement that in H3.1K27M induced H3K27me3 loss is diffuse. Please comment or amend.
The single cell data comparing H3.1 and H3.2K27M cells confirms the previous finding of Castel et al (Acta Neuropathol 2015). Why did the authors not consider these findings?
Discussion
In Grasso's paper (Nature Med 2015), it was shown that HDACi treatment was reverting the mesenchymal phenotype of the tumors cells in vitro. The authors should comment this information as well since it is supporting the interplay between H3K27Ac/H3K27me3 and mesenchymal drift.

Methods

Are the methods appropriate to the aims of the study, are they well described, and are necessary controls included? Choose an item.

Conclusions

Are the conclusions adequately supported by the data shown? Choose an item.

Reporting Standards

Does the manuscript adhere to the journal's guidelines on minimum standards of reporting? Choose an item.

Choose an item.

Statistics

Are you able to assess all statistics in the manuscript, including the appropriateness of statistical tests used? Choose an item.

Quality of Written English

Please indicate the quality of language in the manuscript: Choose an item.

Declaration of Competing Interests

Please complete a declaration of competing interests, considering the following questions:

- Have you in the past five years received reimbursements, fees, funding, or salary from an organisation that may in any way gain or lose financially from the publication of this manuscript, either now or in the future?
- Do you hold any stocks or shares in an organisation that may in any way gain or lose financially from the publication of this manuscript, either now or in the future?
- Do you hold or are you currently applying for any patents relating to the content of the manuscript?
- Have you received reimbursements, fees, funding, or salary from an organization that holds or has applied for patents relating to the content of the manuscript?
- Do you have any other financial competing interests?
- Do you have any non-financial competing interests in relation to this paper?

If you can answer no to all of the above, write 'I declare that I have no competing interests' below. If your reply is yes to any, please give details below.

I declare that I have no competing interests.

I agree to the open peer review policy of the journal. I understand that my name will be included on my report to the authors and, if the manuscript is accepted for publication, my named report including any attachments I upload will be posted on the website along with the authors' responses. I agree for my report to be made available under an Open Access Creative Commons CC-BY license (http://creativecommons.org/licenses/by/4.0/). I understand that any comments which I do not wish to be included in my named report can be included as confidential comments to the editors, which will not be published.

Choose an item.

To further support our reviewers, we have joined with Publons, where you can gain additional credit to further highlight your hard work (see: https://publons.com/journal/530/gigascience). On publication of this paper, your review will be automatically added to Publons, you can then choose whether or not to claim your Publons credit. I understand this statement.

Yes Choose an item.
